# PQR309, a dual PI3K/mTOR inhibitor, synergizes with gemcitabine by impairing the GSK-3β and STAT3/HSP60 signaling pathways to treat nasopharyngeal carcinoma

**DOI:** 10.1038/s41419-024-06615-8

**Published:** 2024-03-30

**Authors:** Jiaxin Cao, Kangmei Zeng, Qun Chen, Ting Yang, Feiteng Lu, Chaozhuo Lin, Jianhua Zhan, Wenjuan Ma, Ting Zhou, Yan Huang, Fan Luo, Hongyun Zhao

**Affiliations:** State Key Laboratory of Oncology in South China, Guangdong Key Laboratory of Nasopharyngeal Carcinoma Diagnosis and Therapy, Guangdong Provincial Clinical Research Center for Cancer, Sun Yat-sen University Cancer Center, Guangzhou, 510060 PR China

**Keywords:** Targeted therapies, Tumour angiogenesis

## Abstract

End-stage nasopharyngeal carcinoma (NPC) has unsatisfactory survival. The limited benefit of chemotherapy and the scarcity of targeted drugs are major challenges in NPC. New approaches to treat late-stage NPC are urgently required. In this study, we explored whether the dual PI3K/mTOR inhibitor, PQR309, exerted a favorable antineoplastic effect and sensitized the response to gemcitabine in NPC. We observed that PI3K expression was positive and elevated in 14 NPC cell lines compared with that in normal nasopharygeal cell lines. Patients with NPC with higher PI3K levels displayed poorer prognosis. We subsequently showed that PQR309 alone effectively decreased the viability, invasiveness, and migratory capability of NPC cells and neoplasm development in mice xenograft models, and dose-dependently induced apoptosis. More importantly, PQR309 remarkably strengthened the anti-NPC function of gemcitabine both in vivo and in vitro. Mechanistically, PQR309 sensitized NPC to gemcitabine by increasing caspase pathway-dependent apoptosis, blocking GSK-3β and STAT3/HSP60 signaling, and ablating epithelial-mesenchyme transition. Thus, targeting PI3K/mTOR using PQR309 might represent a treatment option to promote the response to gemcitabine in NPC, and provides a theoretical foundation for the study of targeted drugs combined with chemotherapy for NPC.

## Introduction

Nasopharyngeal carcinoma (NPC) mainly occurs in East and Southeast Asia [1]. Approximately 10% of patients with NPC had already progressed to the middle or late stage at initial diagnosis, leading to a poor overall survival (OS) of 20 months [1]. Currently, a gemcitabine plus platinum regimen has been established as the normative therapy for recurrent or metastatic NPC (RM-NPC), based on the landmark GEM20110714 study [2]. Although patients with advanced NPC initially respond to chemotherapy, drug resistance invariably emerges. Recently, targeted therapies have greatly improved the therapeutic effect in patients with malignant tumors. However, National Comprehensive Cancer Network (NCCN) manuals only advise therapy using epidermal growth factor receptor targeted inhibitors for advanced NPC [3]. Therefore, novel therapeutic strategies with good efficacy in patients with RM-NPC are required.

Gemcitabine, a cytotoxic deoxycytidine nucleoside analog of fluorinated pyrimidine, is a backbone chemotherapeutic agent for NPC, with tolerable adverse effects [2, 4, 5]. Monotherapy gemcitabine, which was recommended as salvage treatment in the NCCN guidelines [3], is also available for RM-NPC, exerting an overall response rate of 28–44% [4, 5]. However, survival benefit leaves much to be desired, with the median time to progression ranging from 3.6 to 5.1 months [4, 5]. Consequently, novel effective systemic regimens that enhance the efficacy of gemcitabine for NPC are required urgently.

The phosphatidylinositol 3-kinase (PI3K)/mammalian target of rapamycin (mTOR) signaling pathway has a significant impact on multiple physiological functions, from cell differentiation to development and metabolism [6]. Moreover, previous pre-clinical studies suggested excitation of PI3K/mTOR signal path has a vital function in NPC cells [7, 8]. Studies have claimed that the PI3K/mTOR pathway correlated markedly with inferior survival in patients with advanced NPC [8–10]. Molecular targeted therapeutics to block PI3K and mTOR are being investigated intensively [11–13]. Recent studies demonstrated the limited monotherapy effectiveness of agents that inhibited PI3K or mTOR at tolerated dosages. Resistance to PI3K suppressant could occur because of persistent activation of mTOR signaling [14]. In addition, mTORC1 inhibition could induce excitation of protein kinase B (AKT) by reducing the negative regulation of other pathways that rely on mTOR [15, 16]. Therefore, PI3K/mTOR depressor is expected to damage AKT pathways, thereby reversing PI3K-independent mTOR activation, which could exhibit promising therapeutic potential [17]. Preclinical studies reported several dual PI3K/mTOR inhibitors that effectively inhibited NPC cell proliferation, including BEZ235 [18], PF-04691502 [19], GSK2126458, and PKI-587 [20]. The association of dual PI3K/mTOR inhibitors with chemotherapy was tested in the combination of BEZ235 and cisplatin [18], as well as PF-04691502 plus cisplatin or paclitaxel [19]. The results showed that the combinations synergistically inhibited NPC in vitro and in vivo [18, 19]. However, whether these PI3K/mTOR inhibitors affect the antitumor activity of gemcitabine remains unknown, thus warranting detailed investigation.

PQR309 is a compound showing highly potent PI3K inhibition and moderately potent mTOR inhibition, with some preference for the PI3K alpha subunit (PI3Kα or PI3K p110α) to avoid the feedback of PI3K re-activation and mTOR2-mediated AKT re-activation [21]. PQR309 was identified as a candidate therapy in oncology, including lymphoma and glioblastoma [22–24]. PQR309 is currently under investigation as a single agent or in combined regimens for several types of cancer in clinical trials, showing favorable efficacy and safety [23, 24]. However, whether PQR309 is effective in patients with NPC and could act synergistically with gemcitabine has not been studied.

Herein, the antitumor role of the PQR309 was notarized in NPC cells. PQR309 was verified to suppress cancer development in vivo and in vitro. Most importantly, this study confirmed the synergistic antitumor activity of PQR309 combined with gemcitabine in NPC. Together, these data indicated that PQR309, alone or in combination with gemcitabine, is a potential therapeutic method in NPC.

## Results

### Gene analysis of *PIK3CA* in NPC cells, and PI3K p110α and p110β protein levels in NPC cells and patient samples

**Fig. 1:**
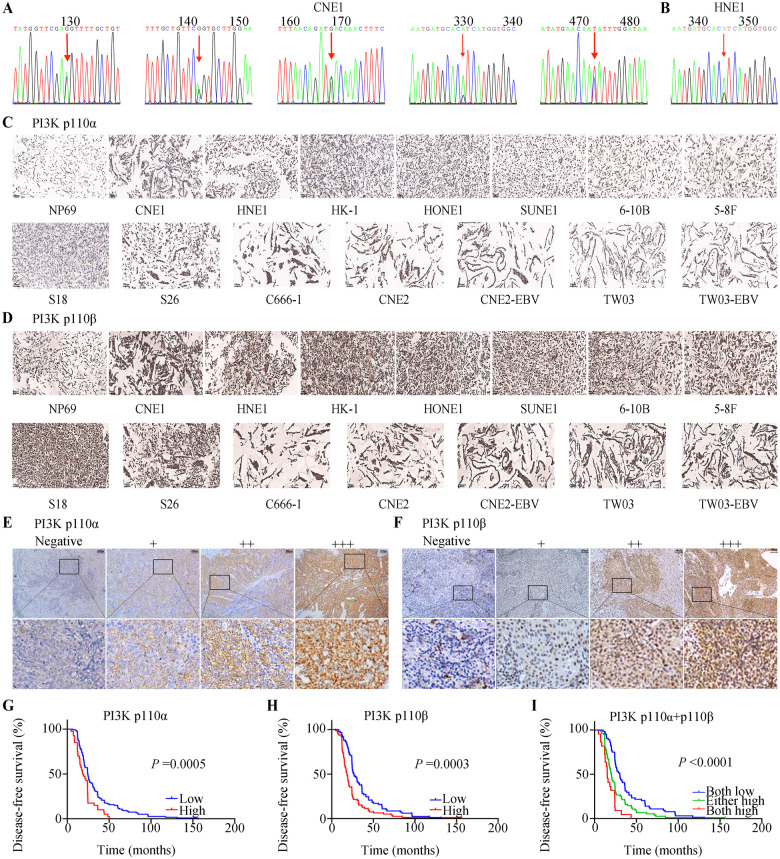
Gene analysis of *PIK3CA* in NPC cells, and PI3K p110α and p110β protein levels in NPC cells and patient samples. **A**, **B** *PIK3CA* mutation frequency was high in CNE1 and HNE1 cells. **C**, **D** Positive p110α and p110β expression in NP69 and 14 NPC cell lines (CNE1, HNE1, HK-1, HONE-1, SUNE1, 6-10B, 5-8 F, S18, S26, C666-1, CNE2, CNE2-EBV, TW03, and TW03-EBV) (×20). Scale bars: 50 μm. **E**, **F** IHC staining demonstrating negative and different intensities of positive expression of PI3K p110α and PI3K p110β (×10). Scale bars: 100 μm. **G** There was a significant difference in DFS between NPC cases with PI3K p110α with a score lower than eight and those with PI3K p110α with a score greater than eight (*P* = 0.0005). **H** There was a significant difference in DFS between NPC cases with PI3K p110β with a score lower than six and those with PI3K p110β with a score higher than six (*P* = 0.0003). **I** There was a significant difference in DFS between NOC cases with lower PI3K p110α and PI3K p110β expression levels and those with higher PI3K p110α and PI3K p110β expression levels (*P* < 0.0001). NPC Nasopharyngeal carcinoma, IHC Immunohistochemistry, DFS Disease-free survival.

PIK3CA is known to activate the PI3K pathway [25, 26], which is associated with somatic mutations in breast, colorectal, gastric, and some brain cancers [27]. Besides, *PIK3CA* was reported to be associated with NPC distant metastasis [28]. We conducted Sanger sequencing to better investigate *PIK3CA* mutations in 12 human NPC cell lines. Remarkably, our study discovered high frequency of *PIK3CA* mutations in miscellaneous NPC cells (CNE1, HNE1, HONE1, SUNE1, 6–10B, 5–8 F, S18, S26, CNE2, TW03). In detail, CNE1 exhibited amino acid variation in Exon 21 p.His1047 Arg, Exon 13 p.Tyr644His, and Exon 12 p.Gly613Ser, p.Arg617Gln and p.Asp626Asn; the amino acid variations of other cell lines were shown to be Exon 21 p.His 1047 Arg (Fig. 1A, B, Supplementary Fig. S1A–H and Supplementary Table S1).

*PIK3CA* encodes the p110α subunit of the PI3 kinase and activates PI3K/AKT/mTOR signaling [29]. The PI3K holoenzyme is formed from P85 and P110 [30]. Among them, p110α and p110β are the major catalytic subunit isomers [31]. Therefore, we next investigated the protein levels of p110α/p110β in 14 NPC cell lines. We observed that p110α and p110β expression was positive and elevated in the 14 NPC cell lines in comparison with that in the NP69 normal nasopharygeal epithelial cells; there was no difference in p110α and p110β expression between EBV positive and negative NPC cells (Fig. 1C, D). Our previous study summarized the results of hematoxylin-eosin (HE) staining in the 14 NPC cell lines [32]. Expression of PI3K was further tested via IHC staining in 161 NPC tissue samples from SYSUCC with clinicopathological features (Supplementary Table S2). Figure 1E, F show representative images of various IHC staining intensities of PI3K p110α and PI3K p110β. The X-tile application was employed to assess optimum cut-off values, and an IHC score of 8 for PI3K p110α and 6 for PI3K p110β were obtained as the optimum cut-off values for high/low expression (data not shown). Survival analyses revealed that patients, who had a high level of PI3K p110α or PI3K p110β had a shorter DFS than those with low PI3K p110α (median DFS: 18.9 *vs*. 24.6 months, *P* = 0.0005, Fig. 1G) or PI3K p110β expression (median DFS: 18 *vs*. 26.7months, *P* = 0.0003, Fig. 1H). Patients who had both lower PI3K p110α and lower PI3K p110β expression had prolonged DFS compared with that of patients who had either higher PI3K p110α or PI3K p110β or both (median DFS: 30.2 *vs*. 20.2 *vs*. 15.25 months, *P* < 0.001, Fig. 1I). The univariate analysis suggested that age, PI3K p110α expression, and PI3K p110β expression correlated significantly with DFS of patients with NPC (*P* < 0.05, Supplementary Table S3). Multivariate analysis showed that age, PI3K p110α expression, and PI3K p110β expression were independent significant prognostic predictors for DFS (Age: hazard ratio (HR) = 1.658, 95% confidence interval (CI): 1.043–2.635, *P* = 0.033; PI3K p110α expression: HR = 1.841, 95% CI: 1.268–2.672, *P* = 0.001; PI3K p110β expression: HR = 1.811, 95% CI: 1.317–2.489, *P* = 0.00026, Supplementary Table S3). These data indicated that suppression of PI3K p110α and/or PI3K p110β might have potential antitumor activity in the management of NPC.

### PQR309 suppresses growth, induces apoptosis, and inhibits the invasiveness and migratory capabilities of NPC cell lines

The small molecule PI3K/mTOR dual inhibitor PQR309 was applied to test its therapeutic potential in NPC cells. The influence of PQR309 on cell viability was investigated among 14 human NPC cell lines. The 50% inhibitory concentration (IC50) for PQR309 in *PIK3CA* mutant NPC cell lines ranged from 0.008617 μM to 0.6002 μM, while the IC50 values in HK-1 and C666-1 cells with wild-type *PIK3CA* were 0.01342 and 0.01495 μM, respectively. The results indicate sensitivity to PQR309 was not influenced by the presence of a mutated or wild-type gene. Notably, the IC50 values in CNE1 and HNE1 cells were relatively higher than those in other NPC cells. In addition, CNE2-EBV and TW03-EBV cells showed slightly higher IC50 values than CNE2 and TW03 cells (Supplementary Fig. S2A–N, Supplementary Table S4). Considering that PQR309 is a pan-PI3K inhibitor, we selected isoform-specific PI3K inhibitors (BYL719 for PI3K p110α, GSK2636771 for PI3K p110β) both of which have entered clinical assessment to further compare their anti-tumor activities. We assessed the ability of the two isoform-specific PI3K inhibitors to suppress proliferation and viability in a panel of 12 NPC cell lines (Supplementary Table S5). Overall, the IC50 values for PQR309 in all NPC cell lines were lower than those for BYL719 and GSK2636771.

**Fig. 2:**
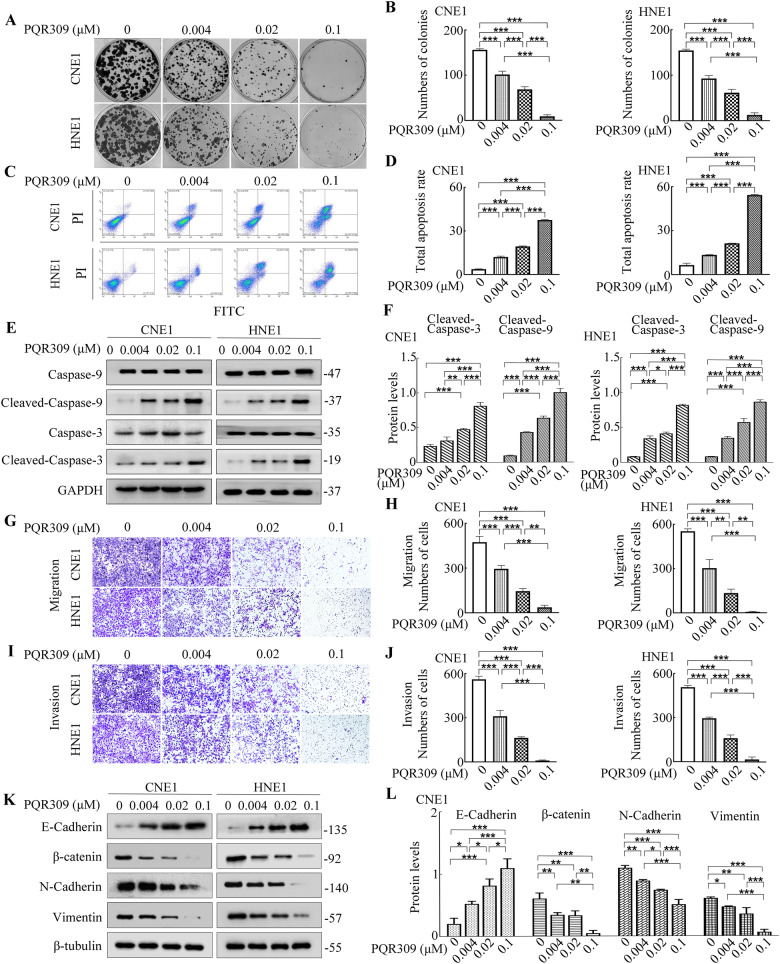
PQR309 suppresses the growth, invasion, and migration and enhances apoptosis in NPC cells. **A**, **B** PQR309 inhibits colony formation of NPC cells in a dose-dependent way. **C**, **D** Annexin V/PI analysis of CNE1 and HNE1 cells treated with different concentrations of PQR309. **E**, **F** Western blotting was applied to detect cleaved caspase-3 and capase-9 levels (apoptotic indexes). **G**, **H** Invasion assays of CNE1 and HNE1 cells treated with different concentrations of PQR309. **I**, **J** Migration assays of CNE1 and HNE1 cells treated with different concentrations of PQR309. **K**, **L** Western blotting was applied to detect EMT markers in CNE1 and HNE1 cells treated with different concentrations of PQR309. All *P < 0.05, **P < 0.01, ***P < 0.001. The error bars represent the standard deviations of three independent experiments. NPC Nasopharyngeal carcinoma, EMT Epithelial-mesenchyme transition.

Using CNE1 and HNE1 cell lines which exhibited relatively higher PI3K p110α and PI3K p110β expressions (Supplementary Fig. S2O), we observed that treatment with PQR309 resulted in markedly fewer colonies and this effect was dose-dependent (Fig. 2A, B). When treated with PQR309, apoptosis was observed to increase significantly in a dose-dependent pattern for CNE1 and HNE1 cells (Fig. 2C, D). Western blotting showed that PQR309 induced apoptosis through excitation of apoptosis-promoting caspase-3 and -9 (Fig. 2E, F). Exposure to increasing doses of PQR309 resulted in a marked dose-dependent decline in the invasive and migrate capabilities of CNE1 and HNE1 (Fig. 2G–J). Epithelial‑mesenchymal transition (EMT) exerts a vital function in the regulation of malignant cell ability of invasive and migrate. To determine PQR309’s impact on the levels of proteins involved in EMT, we treated CNE1 and HNE1 cells with increasing doses of PQR309 and evaluated the levels of mesenchymal markers (β-catenin, vimentin, N-cadherin) and an epithelial marker (E-cadherin). E-cadherin was upregulated, while β-catenin, vimentin, and N-cadherin were downregulated by treatment with increasing doses of PQR309 (Fig. 2K, L and Supplementary Fig. S2P). Collectively, these findings indicated that PQR309 had favorable antitumor effects in vitro. To investigate whether the expressions of PI3K subunits are related to the sensitivity to PQR309, we transfected CNE1 and HNE1 cells with siRNA to silence the expression of *PI3K p110α* or *p110β*. We confirmed the downregulation of these two proteins using western blotting (Supplementary Fig. S2Q, R). Furthermore, we observed that the sensitivity to PQR309 remained unchanged despite siRNA targeting *PI3K p110α* or *p110β*. This was evident from the comparable half-maximal IC50 values of PQR309 in NPC cell lines, whether or not they had undergone genetic downregulation of *PI3K p110α* or *p110β* (Supplementary Fig. S2S, T).

### PQR309 suppresses in vivo NPC tumor growth

**Fig. 3:**
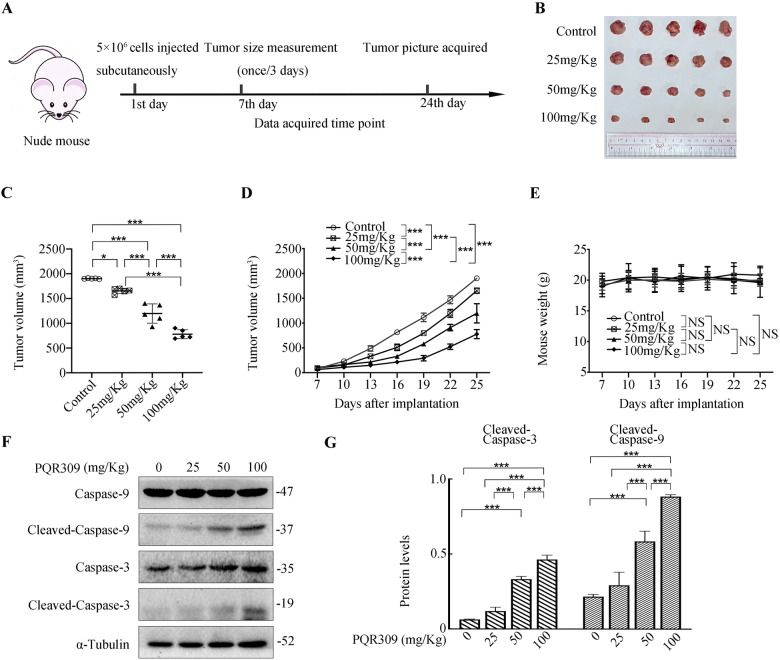
PQR309 inhibits NPC progression in vivo. **A**–**C** Tumor Images and volumes taken at the end of the experiment. **D**, **E** Nude mice with HNE1 xenograft tumors were administered with different concentrations of PQR309 (*n* = 5). **F**, **G** Western blotting analysis of the levels of cleaved caspase-3 and capase-9 from HNE1 xenograft tumors treated with different concentrations of PQR309. All *P < 0.05, **P < 0.01, ***P < 0.001. The error bars represent the standard deviations of three independent experiments.

Next, we examined PQR309’s anti-tumor functions in an NPC tumor xenograft model using HNE1 cells (Fig. 3A). Mice were injected of 5 × 10^6^ human HNE1 NPC cells into their right flank and randomized after 7 days (when tumor size had reached a volume of 100 mm^3^) to receive PQR309 orally daily (25/50/100 mg/kg). Therapy with PQR309 led to a considerable diminution in tumor size: the growth of the tumor was suppressed dose-dependently (Fig. 3B–D). No significant adverse effects, including a loss of body weight, were found among the treated mice (Fig. 3E). Moreover, western blotting of tumor protein lysates showed that caspase-3 and -9 cleavage was augmented dose-dependently in the xenograft tumor tissues (Fig. 3F, G), which agreed with the results obtained in vitro. Thus, the in vivo studies demonstrated that PQR309 also effectively inhibited tumors progression.

### PQR309 combined with gemcitabine exhibits synergistic activities in NPC cell lines

**Fig. 4:**
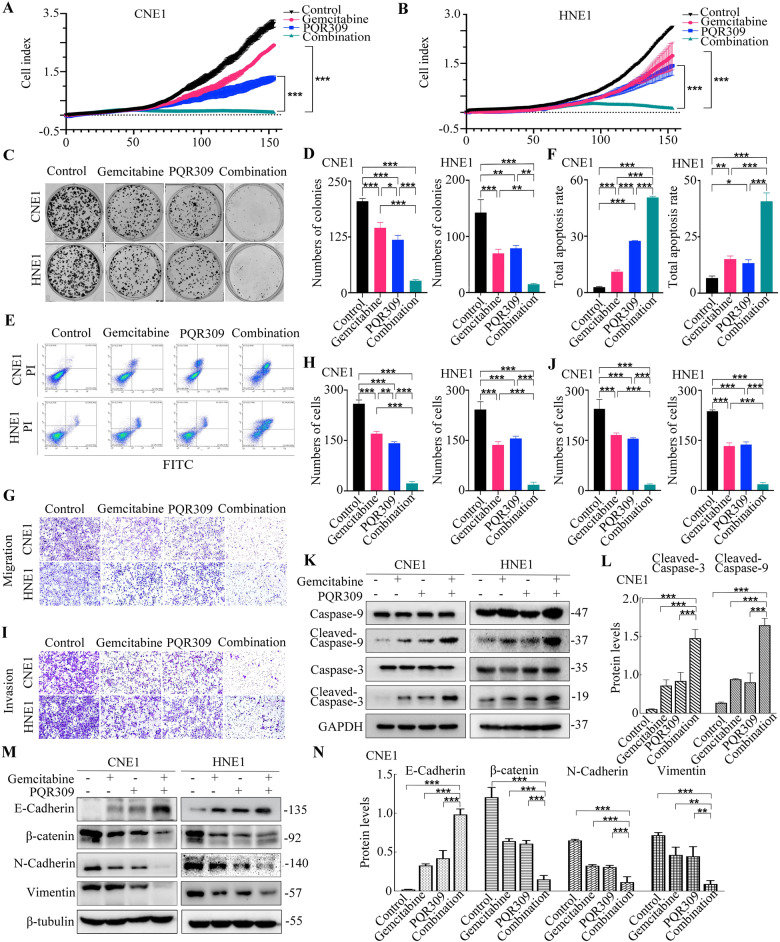
Gemcitabine and PQR309 inhibit the invasiveness and migratory capabilities and EMT of NPC cells. **A**, **B** Gemcitabine and/or PQR309 were administered to CNE1 and HNE1 cells for various times. **C**, **D** Gemcitabine combined with PQR309 significantly inhibited the formation of NPC cell colonies. **E**, **F** Annexin V/PI assessment of CNE1 and HNE1 cells in four groups. **G**–**J** Invasion and migration assays of CNE1 and HNE1 cells in the four groups. **K**, **L** Western blotting was applied to detect cleaved caspase-3 and cleaved caspase-9 (apoptotic markers). **M**, **N** Western blotting was applied to detect EMT markers in CNE1 and HNE1 cells. All *P < 0.05, **P < 0.01, ***P < 0.001. The error bars represent the standard deviations of three independent experiments. NPC nasopharyngeal carcinoma, RTCA Real-Time Cell Analysis, EMT Epithelial-mesenchyme transition.

Next, we assessed whether combination treatment with PQR309 and gemcitabine, which is the standard chemotherapeutic drug, could further reduce the survival of NPC cells, and whether such a combination worked synergistically. The results of Compusyn identification manifested that the combination of PQR309 plus gemcitabine exerted a synergistic cytotoxic effect on CNE1 and HNE1 cells, with combination indices of less than 0.9 at the 50% effective dose (ED50), ED75, and ED90 (Supplementary Table S6). We also investigated whether the isoform-specific PI3K inhibitors could have a synergistic effect with gemcitabine. We found that only the PI3K p110α inhibitor BYL719 exhibited synergy with gemcitabine, with combination indices of less than 0.9 at the ED50, ED75, and ED90 (Supplementary Table S6). Next, RTCA identified that CNE1 and HNE1 cell proliferation was reduced significantly when they were treated with either PQR309 or gemcitabine. Moreover, enhanced antiproliferative effects were exerted by the combination treatment of PQR309 and gemcitabine on both CNE1 and HNE1 cell lines compared with either drug alone (Fig. 4A, B). Combined therapy with PQR309 and gemcitabine significantly reduced the colony numbers in contrast to treatment by either drug alone (Fig. 4C, D).

Next, the effects of the drug combination on apoptosis were evaluated. Analysis of apoptosis using the Annexin-V/PI assay showed significantly higher apoptosis activity in both CNE1 and HNE1 cells under combination treatment with PQR309 and gemcitabine compared with that under either compound alone (Fig. 4E, F). To verify the impact of PQR309 and gemcitabine on migratory behavior, Transwell assays were performed in CNE1 and HNE1 cells. Both CNE1 and HNE1 cells showed a significant additional reduction in migration and invasion activity after exposure to PQR309 plus gemcitabine compared with that after exposure to either drug alone (Fig. 4G–J). Western blotting analysis for cleaved-caspase-3 and -9 confirmed that the induction of apoptosis by PQR309 plus gemcitabine in CNE1 and HNE1 cells proceeded in a caspase-dependent fashion, as observed by increased caspase-3 cleavage and caspase-9 cleavage in the PQR309+gemcitabine group (Fig. 4K, L and Supplementary Fig. S2U). To determine the ability of PQR309 plus gemcitabine to reverse EMT, the levels of several EMT indicators were determined by western blotting. PQR309 plus gemcitabine additionally reduced EMT via downregulating N-cadherin, vimentin, β-catenin (mesenchymal markers), and upregulating E-cadherin (epithelial symbol) compared with treatment using either single drug (Fig. 4M, N and Supplementary Fig. S2V).

Importantly, we also assessed whether the combination of PQR309 with gemcitabine could exhibit synergistic anti-tumor effects in HK-1 and C666-1 cells with wild-type *PIK3CA*. This combined therapy significantly increased apoptosis, anti-colony-forming effects, and notably inhibited NPC cell migration and invasion. These findings are consistent with the synergy observed between PQR309 and gemcitabine in CNE1 and HNE1 cells with mutant *PIK3CA* (Supplementary Fig. S3A–H). These results indicate that PQR309 enhances the anti-tumor efficacy of gemcitabine in NPC, regardless of whether the cell lines harbor *PIK3CA* mutations or not.

### PQR309 combined with gemcitabine inhibits the progression of malignancy in vivo

**Fig. 5:**
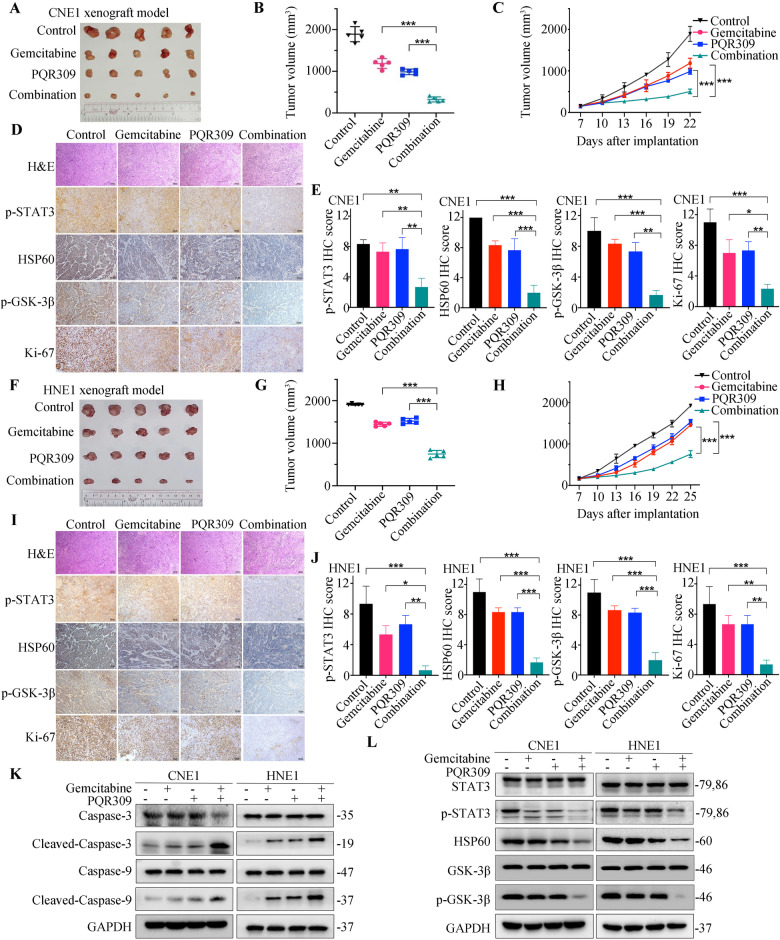
Gemcitabine and PQR309 suppress NPC development in vivo. **A**, **B**, **F**, **G** Tumor Images and volumes at the end of the experiment. **C**, **H** Nude mice with CNE1 and HNE1 xenograft tumors were treated in four groups (*n* = 5). **D**, **E**, **I**, **J** HE and IHC analysis were performed in xenograft tumor models. **K**, **L** Western blotting of cleaved caspase-3, cleaved caspase-9, STAT3, p-STAT3, HSP60, p-GSK-3β, and GSK-3β from two xenograft tumors. All *P < 0.05, **P < 0.01, ***P < 0.001. The error bars represent the standard deviations of three independent experiments. NPC nasopharyngeal carcinoma, HE hematoxylin and eosin, IHC immunohistochemistry, HSP60 heat shock protein 60.

To assess activity of the drug combination in vivo, BALB/c mice with CNE1 or HNE1 subcutaneous xenografts were assigned to four groups receiving PQR309 and/or gemcitabine. Treatments with either gemcitabine or PQR309 alone suppressed subcutaneous tumor growth moderately, whereas combination therapy with PQR309 and gemcitabine suppressed cancer growth compared with either monotherapy. A highly significant inhibition of xenograft growth under treatment by the drug combination was observed (Fig. 5A–C, F–H). During the experiment, the mice’s internal organs displayed no obvious pathological changes according to hematoxylin and eosin (H&E) staining (Supplementary Fig. S4). We further compared the efficacy and toxicity of PQR309 or BYL719 in combination with gemcitabine or alone. Tumor-bearing mice were treated with PQR309, BYL719, and gemcitabine either individually, or in combination. As expected, compared to the control treated mice, tumor growth was effectively inhibited in mice treated with PQR309, BYL719, or gemcitabine alone. However, the combination of PQR309 and gemcitabine led to optimal tumor regression and outperformed both the control group and monotherapy (Supplementary Fig. S5A–C). Importantly, whether gemcitabine was combined with PQR309 or BYL719, the combination therapy was well-tolerated, as evidenced by the fact that the body weight of all mice under the various treatments showed no significant changes (Supplementary Fig. S5D). Additionally, we harvested various organs, including the brain, heart, lung, stomach, gut, liver, kidney, and spleen, and conducted H&E staining to pathologically assess the toxicity profile. We found no obvious structural abnormalities (Supplementary Fig. S5E). Collectively, our data suggest that the pan-PI3K inhibitor PQR309, whether used as monotherapy or in combination with gemcitabine, can optimize anti-tumor efficacy without leading to increased in vivo toxicity when compared to the isoform-specific PI3K inhibitor BYL719.

Moreover, the percentages of p-STAT3, HSP60, p-GSK-3β, and Ki67 (a proliferation marker)-positive cells decreased under PQR309 or gemcitabine treatment, and were further decreased after the combined treatment (Fig. 5D, E, I, J). Western blotting showed elevated levels of cleaved-caspase-3 and capase-9 in the PQR309 plus gemcitabine group in comparison with those in control and single agent-treated groups (Fig. 5K and Supplementary Fig. S2W, X). Furthermore, combined treatment with PQR309 and gemcitabine reduced p-STAT3, HSP60, p-GSK-3β protein levels in the xenograft tumor tissues, according to western blotting analysis (Fig. 5L and Supplementary Fig. S2Y, Z). Taken together, combined treatment with PQR309 and gemcitabine could synergistically inhibit growth, reduce proliferation, and induce the apoptosis of NPC tumors in nude mice.

### PQR309 combined with gemcitabine inhibits phosphorylation of GSK-3β, and STAT3/HSP60 signaling in NPC

**Fig. 6:**
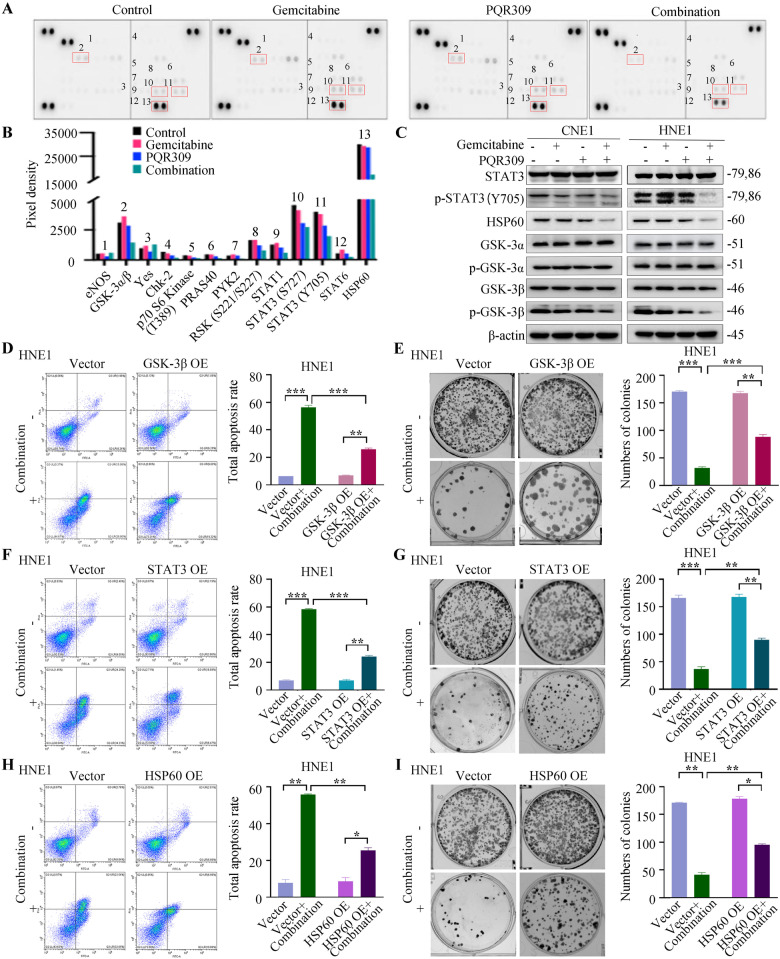
Gemcitabine and PQR309 exert a more potent antineoplastic effect by inhibiting the GSK-3β and STAT3/HSP60 signaling pathway. **A**, **B** Protein lysates from the four groups of HNE1 cells were tested using a phospho-kinase array kit. **C** Western blot analysis of STAT3, GSK-3β, and HSP60 in CNE1 and HNE1 cells. **D**, **F**, **H** Apoptotic assay results of HNE1 cells transfected with the *GSK-3β, STAT3*, and *HSP60* overexpression vectors. **E**, **G**, **I** Quantification of the indicated cells (scramble control, *GSK-3β, STAT3*, and *HSP60* overexpression vectors) after crystal violet staining. Data are shown as the mean ± SD. All *P < 0.05, **P < 0.01, ***P < 0.001. The error bars represent the standard deviations of three independent experiments. HSP60, Heat Shock Protein 60.

To explore the underlying mechanism of the synergistic antitumor effect observed with the combined treatment, we initially evaluated its impact on the PI3K/mTOR pathway. As expected, we observed that the substrate levels of the PI3K/mTOR pathway, including those of p-mTOR (S2448/S2481), p-AKT (S473), p-S6 (S235/S236), p-4E-BP1 (T37/T46), p-S6K (T389), p-FOXO1 (S256), p-FOXO3a (S318/S321), p-FOXO4 (S197), were inhibited by PQR309 alone. However, the levels of these proteins in the combination group did not further decrease compared to PQR309 alone (Supplementary Fig. S6A–E). These data suggest that the PI3K/mTOR pathway might not be involved in the synergistic antitumor mechanism mediated by PQR309 and gemcitabine. Next, the synergistic effect of PQR309 and gemcitabine in HNE1 cells on phosphorylated proteins was investigated with the aid of a human phosphor-kinase array kit (Fig. 6A, B). Decreased phosphorylation of several proteins was observed in response to combined treatment with PQR309 and gemcitabine. In addition, elevated levels of phosphorylated endothelial nitric oxide synthase (eNOS) and YES were observed in the combined treatment group. Among these proteins, we further utilized western blotting to verify the most significantly reduced phosphorylation of three proteins: STAT3, HSP60, and GSK-3α/β. Consistent with previous results, levels of p-STAT3/HSP60 were downregulated by treatment with the drug combination to a higher degree than those under either treatment alone. Meanwhile, the combined treatment dramatically decreased the level of p-GSK-3β, but did not affect GSK-3α phosphorylation (Fig. 6C and Supplementary Fig. S6F, G).

Given that the expression of p-GSK-3β was reduced by the combined therapy, we further established stable NPC cells with either *GSK-3β* overexpression or knockdown, as confirmed by western blotting (Supplementary Fig. S7A, B, E, F). Apoptosis and the colony formation ability of these *GSK-3β*-modified NPC cells were determined by flow cytometric and colony formation assays. We observed that the promotion of tumor apoptosis and the reduction in cell colony formation induced by PQR309 plus gemcitabine were partially rescued by the overexpression of *GSK-3β* (Fig. 6D, E, Supplementary Fig. S7C, D). Conversely shRNAs-mediated knockdown of *GSK-3β* significantly increased apoptotic cell percentages and inhibited NPC cell colony formation after treatment with PQR309 and gemcitabine (Supplementary Fig. S7G–J). These results indicate that PQR309 affects the efficacy of gemcitabine via the downregulation of *GSK-3β*.

Recent findings suggest that the *HSP60* gene promoter has a STAT3 binding site [33] and that functional STAT3 signaling can induce *HSP60* expression [34]. Therefore, the downregulation of p-STAT3 levels by treatment with PQR309 plus gemcitabine might affect the transcription and expression levels of HSP60. To confirm the possible mechanisms of STAT3 involvement in gemcitabine sensitivity, we established *STAT3*-overexpressing NPC cells (Supplementary Fig. S8A, B). We then determined the impact of *STAT3*-overexpression on HSP60 regulation, which significantly increased HSP60 levels in both CNE1 and HNE1 cells (Supplementary Fig. S8A, B). As presented in Fig. 6F, G and Supplementary Fig. S8C, D, the apoptosis rate was higher in the PQR309 + gemcitabine group than in the PQR309 + gemcitabine + *STAT3*-overexpressing group, and the number of colonies was larger in the PQR309 + gemcitabine + *STAT3*-overexpressing group. Furthermore, we transfected shRNAs targeting *STAT3* into CNE1 and HNE1 cells, followed by treatment with PQR309 plus gemcitabine. The effectiveness of *STAT3* knockdown was verified using western blotting, and *STAT3* shRNA significantly reduced HSP60 levels in CNE1 and HNE1 cells (Supplementary Fig. S8E, F). *STAT3* knockdown enhanced the antitumor effect in terms of apoptosis induced by the combined treatment (Supplementary Fig. S8G, H). Additionally, *STAT3* knockdown significantly potentiated the inhibitory effect induced by PQR309 plus gemcitabine on colony formation in CNE1 and HNE1 cells (Supplementary Fig. S8I, J). The aforementioned results indicate that STAT3 is involved in the synergistic effect of PQR309 and gemcitabine.

Considering the previously described importance of HSP60 in cancer cells [35] and our data showing that *STAT3* overexpression/knockdown significantly increased/reduced HSP60 levels in CNE1 and HNE1 cells, we studied the effect of HSP60 in the therapeutic efficacy of PQR309 + gemcitabine using *HSP60*-overexpressing NPC cells (Supplementary Fig. S9A, B). Annexin V/PI staining illustrated that the percentage of apoptotic cells significantly decreased, and the number of colonies was significantly larger in the PQR309 + gemcitabine + *HSP60*-overexpressing group, indicating the pro-apoptosis and anti-proliferation effect of PQR309 plus gemcitabine were reversed by *HSP60* overexpression (Fig. 6H, I and Supplementary Fig. S9C, D). To further explore the role of HSP60 in this effect, we used a lentiviral system to knock down *HSP60* and observed that *HSP60* knockdown could further enhance the pro-apoptosis and anti-proliferation abilities of PQR309 plus gemcitabine treatment (Supplementary Fig. S9E–J). This suggests the critical role of HSP60 in the synergy of PQR309 plus gemcitabine.

Collectively, these results indicate that the co-administration of PQR309 and gemcitabine exerts synergistic anti-NPC activity through the regulation of the GSK-3β, p-STAT3/HSP60 axis.

## Discussion

The prognosis of RM-NPC is unsatisfactory and such tumors are mainly treated with traditional chemotherapy. For RM-NPC, one of the standard first-line treatment approaches is gemcitabine-based chemotherapy [2]. However, acquired chemoresistance following treatment is a critical factor resulting in poor survival of patients with NPC. Hence, improvements in treatment strategies or pharmaceuticals are still required. Aberrant excitation of PI3K/mTOR signal channels are observed continually in NPC, which stimulates proliferation and enhances the survival response and drug resistance [36–40]. However, the role of PI3K/mTOR retardants (PQR309), in patients with NPC has not been studied. Our study showed that *PIK3CA* is mutated in most NPC cell lines. PI3K p110α and p110β were positively expressed in 14 NPC cell lines at higher levels than in the normal nasopharygeal epithelia cell line NP69. Moreover, higher levels of PI3K p110α and p110β isoforms were related to shorter DFS in NPC. Besides, we identified that PQR309 could inhibit NPC cell growth, invasion, and migration, and induced apoptosis. Furthermore, PQR309 could act synergistically with gemcitabine via blockade GSK-3β and STAT3/HSP60 signaling, and inhibition of EMT. Collectively, PQR309 combined with gemcitabine might be a practical clinical therapy strategy for further clinical study.

The PI3K signaling pathway plays a vital part in tumor growth, development, apoptosis, and the migration and invasion capabilities of various cancer cells [41]. Alterations of PI3K signaling are likely linked with pathological factors related to poor prognosis, including stage, distant metastasis, and tumor size [42]. p110α and p110β, as widely expressed PI3K isoforms, have become the main therapeutic targets in tumors [43]. PI3K/AKT/mTOR signaling has an indispensable regulatory effect on tumor growth, migration, survival, and angiogenesis [44]. Excitation of PI3K/AKT/mTOR signal path contributes to cancer progression or resistance to antitumor therapy [41]. Constitutive PI3K excitation is related to resistance to cytotoxic drugs, which is always associated with apoptosis inhibition [45–47]. Moreover, PI3K/Akt signaling exerts an important function in the chemoresistance of gallbladder cancer to gemcitabine [48]. In addition, excitation of PI3K/AKT/mTOR signal channel triggers gemcitabine resistance in pancreatic cancer [49]. Studies indicated that patients with NPC with PI3K mutations and PI3K signaling activation have shorter survival [38–40, 42], which is consistent with our findings. Originally, we showed that patients with NPC with higher PI3K p110α and PI3K p110β expression had inferior prognosis. Furthermore, the Cox regression model showed that PI3K p110α and PI3K p110β expression levels were independent prognosis indicators for patients with NPC. Likewise, high PI3K p110α and PI3K p110β expression was related to histological classification, metastasis, and worse prognosis in colorectal carcinoma [50], lung cancer [51], and gastric carcinoma [52]. Overall, these results suggested that the PI3K pathway provided prognostic markers for NPC, and thus might be a relevant therapeutic target.

PQR309 is an inhibitor of PI3K subtypes (p110α, p110δ, p110β, and p110γ), and mTORC1/mTORC2 [21]. Previous preclinical studies verified that PQR309 has antineoplastic efficacy in vitro and in vivo toward lymphomas [53], diffuse large B-cell lymphoma [54], glioblastoma [22] and endometrial cancer-derived models [55], which are consistent with our findings. Moreover, we found that the anti-NPC activity of PQR309 was dose-and time-dependent. Other dual PI3K/mTOR inhibitors also showed biological actions in NPC models, such as BEZ235 [18] and PF-04691502 [19]. A completed phase I trial of PQR309 showed clinical responses in 27% of patients with advanced solid tumors [23]. PQR309 showed promising clinical efficacy without dose limiting toxicity in a clinic trial of relapsed/refractory lymphoma [24]. Various clinical trials of PQR309 for lymphoma and solid tumors are ongoing [21]. Besides, PQR309 could cross the blood-brain barrier, thus might show better anti-tumor proliferation effect than other dual PI3K/mTOR inhibitors [21]. These observations indicate that PQR309 can be used as a potential targeted therapy for RM-NPC.

PQR309 was reported to induce a synergistic effect with several other drugs in various tumors [53], suggesting that the combination of PQR309 and other agents might be feasible. Verifying the promising data in vivo, we found that PQR309 synergized with gemcitabine to exert growth inhibition of mouse models bearing NPC cells. Preclinical data from various lymphoma models showed that PQR309 synergized with both targeted and chemotherapy agents [53]. Similarly, Yang et al. showed that another PI3K/mTOR inhibitor, BEZ235, synergistically sensitizes NPC cells to cisplatin [18]. The combination of BEZ235 and gemcitabine has been shown to improve antitumor efficacy in pancreatic cancer [56] and cholangiocarcinoma models [57]. To the best of our knowledge, this is the first study to suggest that PI3K/mTOR inhibitors plus gemcitabine would have a synergistic antitumor effect in patients with NPC.

Herein, PQR309 plus gemcitabine greatly enhanced the induction of apoptosis and significantly activated caspase-9 and caspase-3. Similarly, Yang et al. revealed that the antineoplastic action of PQR309 on glioblastoma was mainly effected through the induction of apoptosis [22]. PQR309-induced gene expression and protein phosphorylation changes related to apoptosis were revealed by proteomics and RNA profiling in lymphoma [53]. Levels of pro-apoptotic proteins (including Bad and cleaved-caspase-9) were increased, while the expressions of anti-apoptotic markers(e.g., MMP-9, MMP-2, and Bcl-2) were decreased in PQR309-treated glioma models [22].

To further understand the underlying mechanism, we investigated the synergistic effects of PQR309 and gemcitabine, and demonstrated that it was the result of regulating the GSK-3β and STAT3/HSP60 pathways. In this study, RTKA, a phospho-tyrosine kinase array, and western blotting demonstrated that PQR309 plus gemcitabine remarkably decreased p-STAT3, HSP60, and p-GSK-3β levels. A growing body of evidence has shown that STAT3 or GSK3β activation promotes tumor development and progression, as well as gemcitabine resistance in pancreatic ductal adenocarcinoma (PDAC) [58, 59]. Recent research has revealed that targeted inhibition of STAT3 or GSK3β in combination with gemcitabine can significantly enhance therapeutic efficacy in PDAC [59, 60]. These findings demonstrate that the expressions of both STAT3 and GSK3β may be associated with gemcitabine sensitivity, and that there might exist a complex crosslink between gemcitabine, STAT3 and GSK3β. STAT3, a major transcription factor that has crucial functions in cell viability, survival, inflammation, immunity, and the growth, invasion and metastatic potential of many malignances, is abnormally activated in various cancer types [61, 62]. In fact, evidence has shown a clear relationship between STAT3 levels and cancer chemotherapy resistance. A recent study reported that elevated STAT3 levels affected gemcitabine resistance in lung cancer [63]. Similarly, STAT3 inhibitors promoted the susceptibility of NPC cells to platinum-based chemotherapy [64]. Recent studies have found that STAT3 can promote carcinogenesis with the coexisting activation of PI3K [65]. Moreover, STAT3 signaling was inhibited when various tumor cell lines were received treatment of mTOR inhibitor rapamycin [66], which supports our observation that STAT3 phosphorylation was inhibited by the combination of PQR309 and gemcitabine. In addition, our findings of co-treatment with the dual PI3K/mTOR inhibitor PQR309 and gemcitabine resulting in p-STAT3 blockage are in line with previously shown evidence of PI3K/mTOR signaling pathway-mediated STAT3 activation [67, 68], and STAT3 inhibitors sensitizing mouse *PIK3CA*-mutant breast cancer to PI3K inhibitors [69].

The typical chaperone, HSP60, aids protein folding in the extracellular space, the cell surface, the cytosol, and the mitochondria. HSP60 is overexpressed in several cancers [70], accelerating cancer development and metastasis [71, 72]. Moreover, HSP60 mediated drug resistance in in vitro models [73] and can be used to predict the response to chemotherapy [74]. Meanwhile, HSP60 was evidenced to exert a regulatory function in patients with cisplatin-resistant ovarian cancer [75], in which high expression of HSP60 can decrease the survival rate after cisplatin chemotherapy. *HSP60* has a STAT3-binding site in its promoter and HSP60 can be regulated in a STAT3-dependent manner [33, 34]. Specifically, we found that PQR309 plus gemcitabine synergistically suppressed NPC tumor activity by downregulating STAT3-mediated HSP60 expression. In addition, HSP60 was reported to be an important regulator of apoptosis [76]. The cytosolic fraction of HSP60 might interact with Bak and Bax, because reduced HSP60 expression correlates with increased Bak and Bax abundance. HSP60 can interact with β-catenin to increase its protein level and enhance its transcriptional activity, thus promoting EMT [72]. EMT inhibition is another important mechanism of antitumor activity induced by PQR309 and/or gemcitabine in NPC. The EMT process enables cell migratory and invasive behavior, which is important for cancer invasion, metastasis, and resistance to cytotoxic therapies [77].

The multifunctional serine/threonine protein kinase GSK-3 has several isoforms, and exerts important functions in cell cycle progression and apoptosis [78]. Previously, much of the focus on the GSK-3 family has been on GSK-3β. Activation of GSK-3β/Wnt/β-catenin signal channels by oncogenes are widely acknowledged to promote EMT and metastasis in NPC [79]. Recent studies suggested that blockade of GSK-3β could suppress the viability of neuroblastomas [80], prostate cancer [81], and lung cancer [82]. Pancreatic cancer cell lines’ innate resistance to gemcitabine is related to low E-cadherin expression [83]. Moreover, acquired resistance to gemcitabine could be mediated by compensatory and treatment-induced changes to EMT [84]. Therefore, targeting EMT is an important strategy to inhibit metastasis and improve the drug response. Furthermore, a previous study indicated that high levels of p-GSK3β are more sensitive to PQR309, and the combination of PQR309 with a GSK3β inhibitor or GSK3β gene silencing had a synergistic anti-tumor effect in glioblastoma [85], suggesting that there may be a regulatory relationship between PQR309 and GSK3β, which could also support our results. Excitingly, in the current study, PQR309 reduced NPC cell proliferation by co-inhibiting EMT through the GSK-3β and STAT3/HSP60 signaling pathways. Moreover, the inhibitory effect was more obvious when combined with gemcitabine, which conformed with a previous report that PQR309 inhibits glioblastoma migration and invasion [22]. These findings illustrated the synergistic effect of PQR309 plus gemcitabine on EMT inhibition, which indicates that the drug combination could be a potential treatment to suppress metastatic NPC.

## Conclusion

**Fig. 7:**
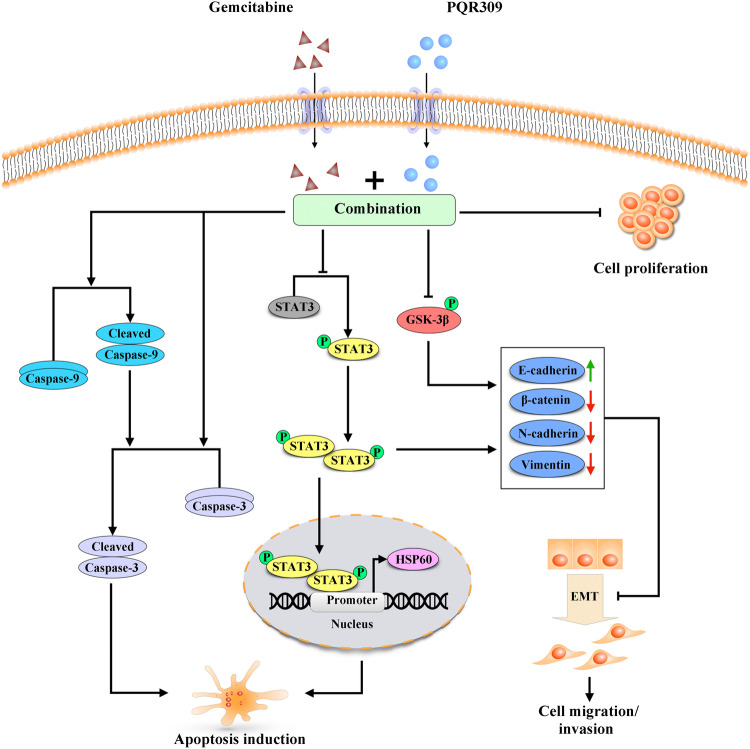
Potential mechanism of the antineoplastic role of PQR309 and gemcitabine in nasopharyngeal carcinoma. PQR309 sensitized NPC to gemcitabine by increasing caspase-dependent pathway apoptosis, blocking GSK-3β and STAT3/HSP60 signaling pathways, and inhibiting EMT. NPC Nasopharyngeal carcinoma; HSP60 Heat shock protein 60, EMT Epithelial-mesenchyme transition.

Herein, we reported that PQR309, a dual PI3K/mTOR inhibitor, suppressed the ability of NPC cells to migrate and invade, and induced their apoptosis. Our findings indicated that PQR309 significantly enhanced the anti-invasion and anti-growth activities of gemcitabine against NPC in vivo *and* in vitro. Furthermore, PQR309 sensitized NPC to gemcitabine by increasing caspase-dependent pathway apoptosis, blocking GSK-3β and STAT3/HSP60 signaling pathways, and inhibiting EMT (Fig. 7). These results suggested that PQR309 plus gemcitabine has synergistic anti-NPC activity, representing a potential treatment option for patients with NPC. The results also provide basic knowledge for subsequent translational clinical research of the targeted drug PQR309 combined other chemotherapeutic agents to treat NPC.

## Materials and methods

### Cell lines

This research applied a normal nasopharyngeal cell line NP69 and 14 human NPC cell lines, including TW03-EBV, TW03, CNE2-EBV, CNE2, C666-1, S26, S18, 5–8F, 6-10B, SUNE1, HONE-1, HK-1, HNE1, and CNE1. Prof. Kaitai Yao (Southern Medical University, Guangzhou, People’s Republic of China) donated the HNE1 cells. The other cell lines were gratefully received from Prof. Musheng Zeng (Sun Yat-Sen University Cancer Center (SYSUCC), Guangzhou, People’s Republic of China). The culture medium for cells transfected with short hairpin RNAs (shRNAs) targeting *STAT3* (encoding signal transducer and activator of transcription 3) was added with 0.5 μg/mL of puromycin. Stock solutions (10 mM) of PQR309 and gemcitabine (Selleck Chemicals, Houston, TX, USA) were made for the in vitro assays. For the in vivo studies, PQR309 was formulated in 0.5% Sodium carboxymethyl cellulose and gemcitabine was diluted in sterile water to 5 mg/mL.

### Human tissue samples

The study cohort comprised of 161 available formalin-fixed, paraffin-embedded samples from patients that had been confirmed pathologically to have NPC, from December 1, 2005 to January 30, 2019 at SYSUCC. All procedures were carried out following the tenets of the Declaration of Helsinki. Informed consent was obtained from all subjects. Included cases should be 18–80 years of age with a pathological diagnosis of NPC and without second primary cancer. Follow-up was done at intervals of three months for two years, every six months for another three years, and annually thereafter until October 2020.

### Tissue microarray (TMA) and immunohistochemistry (IHC) analysis

A TMA (2 mm) involving the aforementioned 15 cell lines was constructed in our laboratory, as described previously [32]. Tumor samples from patients and xenografts from nude mice were subjected to IHC using 3-μm-thick sections. The primary antibodies recognized human PI3K p110α (1:200), human PI3K p110β (1:200), murine proliferation marker Ki-67 (Ki-67) (1:500), murine phosphorylated (p)-STAT3 (p-STAT3) (1:500) (all from Abcam, Cambridge, MA, USA); murine heat shock protein 60 (HSP60) (1:1000) and murine p-glycogen synthase kinase-3β (p-GSK-3β) (1:100) (Cell Signaling Technology, Danvers, MA, USA). The IHC slides were judged under a microscope in a double-blind manner by two pathologists. The H-score was used as the scoring criterium for PI3K p110α, PI3K p110β, p-STAT3, HSP60, and p-GSK-3β. In terms of the expression proportion, scoring 0 indicated expression in less than 5% of cells, scoring 1 indicated expression in 5–25% of cells, scoring 2 (26–50%), scoring 3 (51–75%), scoring 4 ( > 75%). In terms of staining intensity, scoring 0 equated to no staining, scoring 1 was slightly positive (+), scoring 2 was moderately positive (++), and scoring 3 was strongly positive (+++). A final immunostaining score comprised the expression proportion score × the staining intensity score, ranging from 0 to 12.

### Cell viability assays

Viability assays were based on Cell Counting Kit-8. First, cells (2–4 × 10^3^) in 200 μL of culture medium were cultured on 96-well plates during 72 h of exposure to PQR309, gemcitabine, or PQR309 plus gemcitabine. A spectrophotometer was then utilized to record the absorbance of all wells at 450 nm. Triplicate assays were carried out; and the experiment was completed three times in total. Compusyn software (ComboSyn Inc., Paramus, NJ, USA) was used to analyze the effects of the drug combinations by calculating the combination index for each tested concentration, in which a combination index value < 0.9 indicated synergy, = 0.9 indicated additivity, and > 0.9 indicated antagonism [86].

### Colony formation assays

NPC cells (1 × 10^3^ CNE1 and HNE1 cells/well) grown on 6-well dishes were exposed to drugs at the indicated doses. After 7 to 14 days, the formed colonies underwent methanol fixation and crystal violet staining. Colony imaging was carried out under an inverted microscope, and those containing in excess of 50 cells were recorded.

### Flow cytometry detection of apoptosis

The indicated drugs were applied to CNE1 and HNE1 cells (2 × 10^6^ per well) on 6-well plates, and the cells were collected for apoptosis detection after 48 h. An Apoptosis Detection Kit was employed for staining. Apoptotic cells were evaluated by applying flow cytometry. The annexin V-positive population represented apoptotic cells.

### Invasiveness and migration assays

For these assays, Transwell chambers were employed. In the migration assay, 8 × 10^4^ CNE1 cells or 5 × 10^4^ HNE1 cells in Dulbecco’s modified Eagle’s medium (DMEM) without fetal bovine serum (FBS) were placed in the top Transwell chamber with the indicated drugs. Then, DMEM with 20% FBS was placed in the lower compartment. Cell invasiveness assays were performed with Matrigel-coated (Corning Inc., Corning, NY, USA) Transwell chambers. Cells were incubated for 18 to 36 h. Afterwards, a 10 min methanol fixation and a 15 min crystal violet staining at room temperature were performed. Cells were enumerated using ImageJ (NIH, Bethesda, MD, USA). Assays were performed three times independently.

### Real-time cell analysis (RTCA)

An RTCA S16 kit for continuous cellular proliferation detection was used (Acea biosciences, San Diego, CA, USA). First, 50 μL of DMEM was placed in the wells of the microelectrodes in the E-plate to obtain the impedance background. Second, 1 × 10^3^ CNE1 or HNE1 cells were seeded into each well before drug treatment. For each drug regimen, two wells were used for the assay. The E-plate was then left to equilibrate for 30 min at room temperature, before incubation in the RTCA workstation. The impedance was monitored at 15-minute time intervals for 120 h. The cell index value was computed to indicate cell proliferation.

### Western blotting analysis

The agents (at the indicted concentrations) were used to treat CNE1 and HNE1 for 48 h. For western blotting, primary antibodies recognizing caspase-3, cleaved-caspase-3, caspase-9, cleaved-caspase-9, E-cadherin, β-catenin, N-cadherin, Vimentin, STAT3, phosphorylated (p)-STAT3, HSP60, GSK-3α, p-GSK-3α, GSK-3β and p-GSK-3β (Cell Signaling Technology; 1:1000) were used. The controls comprised antibodies against β-actin, glyceraldehyde-3-phosphate dehydrogenase (GAPDH), α-tubulin, and β-tubulin (Cell Signaling Technology). Supplementary Table S7 details the antibodies. The secondary antibodies comprised horseradish peroxidase (HRP)-attached goat anti-mouse or anti-rabbit antibodies (1:5000 dilution; Santa Cruz Biotechnology, Santa Cruz, CA, USA).

### Cell transfection

Three shRNAs targeting *STAT3* were cloned and incorporated into vectors using a pSIH-H1-puro Lentivector Packaging Kit, following the supplier’s protocol. The sequences of the shRNAs targeting *STAT3* were:

5’-TCTCTGCAGAATTCAA-3’ (STAT3sh1)

5’-CAGGCTGGTAATTTATATAAT-3’ (STAT3sh2)

5’- GGCGTCCAGTTCACTACTA-3’ (STAT3sh3)

The sequence of the shRNA scrambled control (sc-shRNA) was 5’-CAACAAGATGAAGAGCACCAA-3’. Lentiviral vectors encoding the scrambled control or STAT3-shRNA were transfected into logarithmically growing NPC cells.

For stable knockdown of *GSK3β* and *HSP60*, shRNAs (LPP-HSH099777-LVRU6GP-200 for GSK3β and LPP-HSH009098-LVRU6GP-200 for HSP60) were used, along with the relative scramble controls (LPP-CSHCTR001-LVRU6GP-200). These reagents were obtained from GeneCopoeia (Rockville, MD, US)

To overexpress *STAT3, GSK3β* and *HSP60*, overexpression plasmids (EX-Z2835-M02 for *STAT3*, EX-Z0294-M02 for *GSK3β*, and *EX-G017*5-M02 for *HSP60*, GeneCopoeia) were transfected into cells using a Lip3000 (Invitrogen) following the manufacturer’s protocol. EX-NEG-M02 served as the negative control.

After transfection, cells were selected using culture medium supplemented with puromycin. For siRNA transfection (Ribobio and Qingke), cells in the exponential growth phase were seeded into six‐well tissue culture plates (1 × 10^5^ cells/well), and then the cells were transfected with siRNA using OPTI‐MEM I‐reduced serum medium and lipofectamine RNAimax reagent to stably silence PI3K p110α or PI3K p110β expression after 24 h. The siRNA sequences are shown in Supplementary Table S8. Protein levels of STAT3, GSK3β, HSP60, PI3K p110α and PI3K p110β were determined using western blotting.

### Human phospho-kinase array

The relative extent of protein phosphorylation after treatment with the indicated drugs in HNE1 cells was evaluated using a Human Phospho-Kinase Array Kit comprising 37 kinase phosphorylation sites and two total proteins. 5 × 10^6^ HNE1 cells were placed in 10 cm dishes, subjected to treatment with PQR309, gemcitabine, and their combination at the indicated doses, and collected to obtain protein samples after 48 h. The chemiluminescent signals were imaged and quantification was performed using Quantity One Software.

### Nude mouse tumor xenografts

The Institutional Animal Care Committee of SYSUCC granted consent for the mouse experiments. Female nude mice, aged six weeks and weighing 14–18 grams were reared in a specific pathogen-free (SPF) environment. Three xenograft studies were conducted.

The first investigated the activity of single-agent PQR309 in the HNE1 cell line. In this experiment, mice were administered either a sterile water vehicle alone or PQR309 at varying doses (25, 50, and 100 mg/kg).

The other two experiments explored the combination of PQR309 or BYL719 with gemcitabine in the CNE1 or HNE1 cell lines. In these experiments, mice received treatments as follows: a sterile water vehicle alone, PQR309 (50 mg/kg), BYL719 (50 mg/kg), gemcitabine (50 mg/kg), PQR309 (50 mg/kg) combined with gemcitabine (50 mg/kg), and BYL719 (50 mg/kg) combined with gemcitabine (50 mg/kg).

To initiate the experiments, the right-side flanks of mice were injected subcutaneously with 200 μL of phosphate-buffered saline containing 5 × 10^6^ CNE1 or HNE1 cells. Tumor measurements were conducted using electronic calipers, and tumor sizes were estimated using the formula: volume (mm^3^) = (length × width^2^) × 0.5. When the tumors grew to around 100 mm^3^, the animals were randomized into four groups. Mice received PQR309 once a day via oral gavage, or gemcitabine was administered twice a week by intraperitoneal injection. Caliper measurements were performed three times per week until the tumor volume reached 2000 mm^3^.

### Statistical analysis

SPSS Statistics 25 or GraphPad Prism 8 were utilized to carry out the statistical analyses. All quantitative results were described by the mean and standard deviation (SD). The optimal cutoff for determining low or high expression was generated via X-tile software. Associations between protein expression in NPC tissues and patients’ disease-free survival (DFS) were identified utilizing log-rank and Kaplan–Meier analyses. The univariate /multivariate determinations were carried out utilizing Cox proportional risk models. Comparisons among groups were made using one-way and two-way analysis of variance (ANOVA) and Student’s t-test. A *P*-value < 0.05 indicated statistical significance.

### Supplementary information

Supplementary file

Reproducibility checklist

Original Data File-western blots

## Data Availability

The datasets used and analyzed in this study are available from the corresponding author upon reasonable request. The authenticity of this article has been validated by uploading the key raw data onto the Research Data Deposit public platform (www.researchdata.org.cn), with the approval RDD number as RDDB2023807552.

## References

[1] Chen Y-P, Chan AT, Le Q-T, Blanchard P, Sun Y, Ma J (2019). Nasopharyngeal carcinoma. Lancet.

[2] Zhang L, Huang Y, Hong S, Yang Y, Yu G, Jia J (2016). Gemcitabine plus cisplatin versus fluorouracil plus cisplatin in recurrent or metastatic nasopharyngeal carcinoma: a multicentre, randomised, open-label, phase 3 trial. Lancet.

[3] Pfister DG, Spencer S, Adelstein D, Adkins D, Anzai Y, Brizel DM (2020). Head and Neck Cancers, Version 2.2020, NCCN Clinical Practice Guidelines in Oncology. J Natl Compr Canc Netw.

[4] Zhang L, Zhang Y, Huang PY, Xu F, Peng PJ, Guan ZZ (2008). Phase II clinical study of gemcitabine in the treatment of patients with advanced nasopharyngeal carcinoma after the failure of platinum-based chemotherapy. Cancer Chemother Pharm.

[5] Foo KF, Tan EH, Leong SS, Wee JT, Tan T, Fong KW (2002). Gemcitabine in metastatic nasopharyngeal carcinoma of the undifferentiated type. Ann Oncol.

[6] Fruman DA, Rommel C (2014). PI3K and cancer: lessons, challenges and opportunities. Nat Rev Drug Discov.

[7] Zhu JF, Huang W, Yi HM, Xiao T, Li JY, Feng J (2018). Annexin A1-suppressed autophagy promotes nasopharyngeal carcinoma cell invasion and metastasis by PI3K/AKT signaling activation. Cell Death Dis.

[8] Yu JH, Chen L, Yu JY, Luo HQ, Wang L (2019). PI3K-PKB-mTOR hyperactivation in relation to nasopharyngeal carcinoma progression and prognosis. J Cell Biochem.

[9] Chen J, Hu CF, Hou JH, Shao Q, Yan LX, Zhu XF (2010). Epstein-Barr virus-encoded latent membrane protein 1 regulates mTOR signaling pathway genes which predict poor prognosis of nasopharyngeal carcinoma. J Transl Med.

[10] Wang W, Wen Q, Xu L, Xie G, Li J, Luo J (2014). Activation of Akt/mTOR pathway is associated with poor prognosis of nasopharyngeal carcinoma. PLoS One.

[11] Dreyling M, Santoro A, Mollica L, Leppä S, Follows GA, Lenz G (2017). Phosphatidylinositol 3-kinase inhibition by copanlisib in relapsed or refractory indolent lymphoma. J Clin Oncol.

[12] Gopal AK, Kahl BS, De Vos S, Wagner-Johnston ND, Schuster SJ, Jurczak WJ (2014). PI3Kδ inhibition by idelalisib in patients with relapsed indolent lymphoma. N. Engl J Med.

[13] Yao JC, Shah MH, Ito T, Bohas CL, Wolin EM, Van Cutsem E (2011). Everolimus for advanced pancreatic neuroendocrine tumors. N. Engl J Med.

[14] Elkabets M, Vora S, Juric D, Morse N, Mino-Kenudson M, Muranen T (2013). mTORC1 inhibition is required for sensitivity to PI3K p110α inhibitors in PIK3CA-mutant breast cancer. Sci Transl Med.

[15] Zoncu R, Efeyan A, Sabatini DM (2011). mTOR: from growth signal integration to cancer, diabetes and ageing. Nat Rev Mol cell Biol.

[16] O’Reilly KE, Rojo F, She Q-B, Solit D, Mills GB, Smith D (2006). mTOR inhibition induces upstream receptor tyrosine kinase signaling and activates Akt. Cancer Res.

[17] Marone R, Cmiljanovic V, Giese B, Wymann MP (2008). Targeting phosphoinositide 3-kinase—moving towards therapy. Biochimica et Biophysica Acta (BBA)-Proteins Proteom.

[18] Yang F, Qian X-J, Qin W, Deng R, Wu X-Q, Qin J (2013). Dual phosphoinositide 3-kinase/mammalian target of rapamycin inhibitor NVP-BEZ235 has a therapeutic potential and sensitizes cisplatin in nasopharyngeal carcinoma. PLoS One.

[19] Wong CH, Loong HH, Hui CW, Lau CP, Hui EP, Ma BB (2013). Preclinical evaluation of the PI3K-mTOR dual inhibitor PF-04691502 as a novel therapeutic drug in nasopharyngeal carcinoma. Investig N. drugs.

[20] Liu T, Sun Q, Li Q, Yang H, Zhang Y, Wang R (2015). Dual PI3K/mTOR Inhibitors, GSK2126458 and PKI-587, Suppress Tumor Progression and Increase Radiosensitivity in Nasopharyngeal Carcinoma. Mol Cancer Therapeutics.

[21] Beaufils F, Cmiljanovic N, Cmiljanovic V, Bohnacker T, Melone A, Marone R (2017). 5-(4,6-Dimorpholino-1,3,5-triazin-2-yl)-4-(trifluoromethyl)pyridin-2-amine (PQR309), a Potent, Brain-Penetrant, Orally Bioavailable, Pan-Class I PI3K/mTOR Inhibitor as Clinical Candidate in Oncology. J Med Chem.

[22] Yang K, Tang XJ, Xu FF, Liu JH, Tan YQ, Gao L (2020). PI3K/mTORC1/2 inhibitor PQR309 inhibits proliferation and induces apoptosis in human glioblastoma cells. Oncol Rep.

[23] Wicki A, Brown N, Xyrafas A, Bize V, Hawle H, Berardi S (2018). First-in human, phase 1, dose-escalation pharmacokinetic and pharmacodynamic study of the oral dual PI3K and mTORC1/2 inhibitor PQR309 in patients with advanced solid tumors (SAKK 67/13). Eur J Cancer.

[24] Collins GP, Eyre TA, Schmitz-Rohmer D, Townsend W, Popat R, Giulino-Roth L (2021). A Phase II Study to Assess the Safety and Efficacy of the Dual mTORC1/2 and PI3K Inhibitor Bimiralisib (PQR309) in Relapsed, Refractory Lymphoma. Hemasphere.

[25] Madsen RR, Vanhaesebroeck B, Semple RK (2018). Cancer-Associated PIK3CA Mutations in Overgrowth Disorders. Trends Mol Med.

[26] Mjos S, Werner HMJ, Birkeland E, Holst F, Berg A, Halle MK (2017). PIK3CA exon9 mutations associate with reduced survival, and are highly concordant between matching primary tumors and metastases in endometrial cancer. Sci Rep.

[27] Samuels Y, Wang Z, Bardelli A, Silliman N, Ptak J, Szabo S (2004). High frequency of mutations of the PIK3CA gene in human cancers. Science.

[28] Zhou Z, Li P, Zhang X, Xu J, Xu J, Yu S (2022). Mutational landscape of nasopharyngeal carcinoma based on targeted next-generation sequencing: implications for predicting clinical outcomes. Mol Med.

[29] Hu H, Zhu J, Zhong Y, Geng R, Ji Y, Guan Q (2021). PIK3CA mutation confers resistance to chemotherapy in triple-negative breast cancer by inhibiting apoptosis and activating the PI3K/AKT/mTOR signaling pathway. Ann Transl Med.

[30] Zhang L, Li Y, Wang Q, Chen Z, Li X, Wu Z (2020). The PI3K subunits, P110α and P110β are potential targets for overcoming P-gp and BCRP-mediated MDR in cancer. Mol Cancer.

[31] Liu P, Cheng H, Roberts TM, Zhao JJ (2009). Targeting the phosphoinositide 3-kinase pathway in cancer. Nat Rev Drug Discov.

[32] Luo F, Cao J, Lu F, Zeng K, Ma W, Huang Y (2021). Lymphocyte activating gene 3 protein expression in nasopharyngeal carcinoma is correlated with programmed cell death-1 and programmed cell death ligand-1, tumor-infiltrating lymphocytes. Cancer Cell Int.

[33] Kim SW, Kim JB, Kim JH, Lee JK (2007). Interferon-gamma-induced expressions of heat shock protein 60 and heat shock protein 10 in C6 astroglioma cells: identification of the signal transducers and activators of transcription 3-binding site in bidirectional promoter. Neuroreport.

[34] Kleinridders A, Lauritzen HP, Ussar S, Christensen JH, Mori MA, Bross P (2013). Leptin regulation of Hsp60 impacts hypothalamic insulin signaling. J Clin Investig.

[35] Kumar R, Chaudhary AK, Woytash J, Inigo JR, Gokhale AA, Bshara W (2022). A mitochondrial unfolded protein response inhibitor suppresses prostate cancer growth in mice via HSP60. J Clin Invest.

[36] Morrison JA, Gulley ML, Pathmanathan R, Raab-Traub N (2004). Differential Signaling Pathways Are Activated in the Epstein-Barr Virus-Associated Malignancies Nasopharyngeal Carcinoma and Hodgkin Lymphoma. Cancer Res.

[37] Yip WK, Leong VCS, Abdullah MA, Yusoff S, Seow HF (2008). Overexpression of phospho-Akt correlates with phosphorylation of EGF receptor, FKHR and BAD in nasopharyngeal carcinoma. Oncol Rep.

[38] Hui AB-Y, Lo K-W, Teo PM, To K-F, Huang DP (2002). Genome wide detection of oncogene amplifications in nasopharyngeal carcinoma by array based comparative genomic hybridization. Int J Oncol.

[39] Lin D-C, Meng X, Hazawa M, Nagata Y, Varela AM, Xu L (2014). The genomic landscape of nasopharyngeal carcinoma. Nat Genet.

[40] Or YY, Hui AB, To KF, Lam CN, Lo KW (2006). PIK3CA mutations in nasopharyngeal carcinoma. Int J Cancer.

[41] Martini M, De Santis MC, Braccini L, Gulluni F, Hirsch E (2014). PI3K/AKT signaling pathway and cancer: an updated review. Ann Med.

[42] Yu JH, Chen L, Yu JY, Luo HQ, Wang L (2019). PI3K‐PKB‐mTOR hyperactivation in relation to nasopharyngeal carcinoma progression and prognosis. J Cell Biochem.

[43] Zhang B, Luk C, Valadares J, Aronis C, Foukas LC (2021). Dominant Role of PI3K p110α over p110β in Insulin and β-Adrenergic Receptor Signalling. Int J Mol Sci.

[44] Katso R, Okkenhaug K, Ahmadi K, White S, Timms J, Waterfield MD (2001). Cellular function of phosphoinositide 3-kinases: implications for development, homeostasis, and cancer. Annu Rev Cell Dev Biol.

[45] Clark AS, West K, Streicher S, Dennis PA (2002). Constitutive and inducible Akt activity promotes resistance to chemotherapy, trastuzumab, or tamoxifen in breast cancer cells. Mol cancer Therapeutics.

[46] Hu L, Hofmann J, Lu Y, Mills GB, Jaffe RB (2002). Inhibition of phosphatidylinositol 3′-kinase increases efficacy of paclitaxel in in vitro and in vivo ovarian cancer models. Cancer Res.

[47] Brognard J, Clark AS, Ni Y, Dennis PA (2001). Akt/protein kinase B is constitutively active in non-small cell lung cancer cells and promotes cellular survival and resistance to chemotherapy and radiation. Cancer Res.

[48] Ye J, Qi L, Du Z, Yu L, Chen K, Li R (2021). Calreticulin: a potential diagnostic and therapeutic biomarker in gallbladder cancer. Aging (Albany NY).

[49] Guo Y, Wu H, Xiong J, Gou S, Cui J, Peng T (2023). miR-222-3p-containing macrophage-derived extracellular vesicles confer gemcitabine resistance via TSC1-mediated mTOR/AKT/PI3K pathway in pancreatic cancer. Cell Biol Toxicol.

[50] Wen F, He S, Sun C, Li T, Wu S (2014). PIK3CA and PIK3CB expression and relationship with multidrug resistance in colorectal carcinoma. Int J Clin Exp Pathol.

[51] Lee JS, Lee HW, Lee EH, Park MI, Lee JS, Kim MS (2018). Prognostic significance of phosphoinositide 3-kinase p110α and p110β isoforms in non-small cell lung cancer. Int J Clin Exp Pathol.

[52] Kim K, Lee HW (2018). Expression of Phosphoinositide 3-Kinase p110α and p110β Subunits and PIK3CA Mutation in Patients With Advanced Gastric Carcinoma. Appl Immunohistochem Mol Morphol.

[53] Tarantelli C, Gaudio E, Arribas AJ, Kwee I, Hillmann P, Rinaldi A (2018). PQR309 Is a Novel Dual PI3K/mTOR Inhibitor with Preclinical Antitumor Activity in Lymphomas as a Single Agent and in Combination Therapy. Clin Cancer Res.

[54] Aresu L, Ferraresso S, Marconato L, Cascione L, Napoli S, Gaudio E (2019). New molecular and therapeutic insights into canine diffuse large B-cell lymphoma elucidates the role of the dog as a model for human disease. Haematologica.

[55] Hsin IL, Shen HP, Chang HY, Ko JL, Wang PH (2021). Suppression of PI3K/Akt/mTOR/c-Myc/mtp53 Positive Feedback Loop Induces Cell Cycle Arrest by Dual PI3K/mTOR Inhibitor PQR309 in Endometrial Cancer Cell Lines. Cells.

[56] Awasthi N, Yen PL, Schwarz MA, Schwarz RE (2012). The efficacy of a novel, dual PI3K/mTOR inhibitor NVP‐BEZ235 to enhance chemotherapy and antiangiogenic response in pancreatic cancer. J Cell Biochem.

[57] Jang DK, Lee YG, Chae YC, Lee JK, Paik WH, Lee SH (2020). GDC-0980 (apitolisib) treatment with gemcitabine and/or cisplatin synergistically reduces cholangiocarcinoma cell growth by suppressing the PI3K/Akt/mTOR pathway. Biochem Biophys Res Commun.

[58] Wörmann SM, Song L, Ai J, Diakopoulos KN, Kurkowski MU, Görgülü K (2016). Loss of P53 Function Activates JAK2-STAT3 Signaling to Promote Pancreatic Tumor Growth, Stroma Modification, and Gemcitabine Resistance in Mice and Is Associated With Patient Survival. Gastroenterology.

[59] Namba T, Kodama R, Moritomo S, Hoshino T, Mizushima T (2015). Zidovudine, an anti-viral drug, resensitizes gemcitabine-resistant pancreatic cancer cells to gemcitabine by inhibition of the Akt-GSK3β-Snail pathway. Cell Death Dis.

[60] Nagathihalli NS, Castellanos JA, Shi C, Beesetty Y, Reyzer ML, Caprioli R (2015). Signal Transducer and Activator of Transcription 3, Mediated Remodeling of the Tumor Microenvironment Results in Enhanced Tumor Drug Delivery in a Mouse Model of Pancreatic Cancer. Gastroenterology.

[61] Thilakasiri PS, Dmello RS, Nero TL, Parker MW, Ernst M, Chand AL (2021). Repurposing of drugs as STAT3 inhibitors for cancer therapy. Semin Cancer Biol.

[62] Chai EZ, Shanmugam MK, Arfuso F, Dharmarajan A, Wang C, Kumar AP (2016). Targeting transcription factor STAT3 for cancer prevention and therapy. Pharm Ther.

[63] Liu Z, Ma L, Sun Y, Yu W, Wang X (2021). Targeting STAT3 signaling overcomes gefitinib resistance in non-small cell lung cancer. Cell Death Dis.

[64] Pan Y, Zhou F, Zhang R, Claret FX (2020). Correction: Stat3 inhibitor stattic exhibits potent antitumor activity and induces chemo- and radio-sensitivity in nasopharyngeal carcinoma. PLoS One.

[65] Vogt PK, Hart JR (2011). PI3K and STAT3: a new alliance. Cancer Discov.

[66] Wang J, Lv X, Guo X, Dong Y, Peng P, Huang F (2021). Feedback activation of STAT3 limits the response to PI3K/AKT/mTOR inhibitors in PTEN-deficient cancer cells. Oncogenesis.

[67] Lee HJ, Zhuang G, Cao Y, Du P, Kim HJ, Settleman J (2014). Drug resistance via feedback activation of Stat3 in oncogene-addicted cancer cells. Cancer Cell.

[68] Li J, Diao B, Guo S, Huang X, Yang C, Feng Z (2017). VSIG4 inhibits proinflammatory macrophage activation by reprogramming mitochondrial pyruvate metabolism. Nat Commun.

[69] Merino VF, Cho S, Liang X, Park S, Jin K, Chen Q (2017). Inhibitors of STAT3, β-catenin, and IGF-1R sensitize mouse PIK3CA-mutant breast cancer to PI3K inhibitors. Mol Oncol.

[70] Tang Y, Yang Y, Luo J, Liu S, Zhan Y, Zang H (2021). Overexpression of HSP10 correlates with HSP60 and Mcl-1 levels and predicts poor prognosis in non-small cell lung cancer patients. Cancer Biomark.

[71] Cappello F, Conway de Macario E, Marasà L, Zummo G, Macario AJ (2008). Hsp60 expression, new locations, functions and perspectives for cancer diagnosis and therapy. Cancer Biol Ther.

[72] Tsai YP, Yang MH, Huang CH, Chang SY, Chen PM, Liu CJ (2009). Interaction between HSP60 and beta-catenin promotes metastasis. Carcinogenesis.

[73] Abu-Hadid M, Wilkes JD, Elakawi Z, Pendyala L, Perez RP (1997). Relationship between heat shock protein 60 (HSP60) mRNA expression and resistance to platinum analogues in human ovarian and bladder carcinoma cell lines. Cancer Lett.

[74] Sun B, Li G, Yu Q, Liu D, Tang X (2022). HSP60 in cancer: a promising biomarker for diagnosis and a potentially useful target for treatment. J Drug Target.

[75] Kimura E, Enns RE, Thiebaut F, Howell SB (1993). Regulation of HSP60 mRNA expression in a human ovarian carcinoma cell line. Cancer Chemother Pharm.

[76] Tapia O, Riquelme I, Leal P, Sandoval A, Aedo S, Weber H (2014). The PI3K/AKT/mTOR pathway is activated in gastric cancer with potential prognostic and predictive significance. Virchows Arch.

[77] Brabletz T, Kalluri R, Nieto MA, Weinberg RA (2018). EMT in cancer. Nat Rev Cancer.

[78] Park SA, Lee JW, Herbst RS, Koo JS (2016). GSK-3α Is a Novel Target of CREB and CREB-GSK-3α Signaling Participates in Cell Viability in Lung Cancer. PLoS One.

[79] Yang XZ, Chen XM, Zeng LS, Deng J, Ma L, Jin C (2020). Rab1A promotes cancer metastasis and radioresistance through activating GSK-3β/Wnt/β-catenin signaling in nasopharyngeal carcinoma. Aging (Albany NY).

[80] Dickey A, Schleicher S, Leahy K, Hu R, Hallahan D, Thotala DK (2011). GSK-3β inhibition promotes cell death, apoptosis, and in vivo tumor growth delay in neuroblastoma Neuro-2A cell line. J Neurooncol.

[81] Kroon J, in ‘t Veld LS, Buijs JT, Cheung H, van der Horst G, van der Pluijm G (2014). Glycogen synthase kinase-3β inhibition depletes the population of prostate cancer stem/progenitor-like cells and attenuates metastatic growth. Oncotarget.

[82] Zeng J, Liu D, Qiu Z, Huang Y, Chen B, Wang L (2014). GSK3β overexpression indicates poor prognosis and its inhibition reduces cell proliferation and survival of non-small cell lung cancer cells. PLoS One.

[83] El Amrani M, Corfiotti F, Corvaisier M, Vasseur R, Fulbert M, Skrzypczyk C (2019). Gemcitabine-induced epithelial-mesenchymal transition-like changes sustain chemoresistance of pancreatic cancer cells of mesenchymal-like phenotype. Mol Carcinog.

[84] Wang Z, Li Y, Kong D, Banerjee S, Ahmad A, Azmi AS (2009). Acquisition of epithelial-mesenchymal transition phenotype of gemcitabine-resistant pancreatic cancer cells is linked with activation of the notch signaling pathway. Cancer Res.

[85] von Achenbach C, Weller M, Kaulich K, Gramatzki D, Zacher A, Fabbro D (2020). Synergistic growth inhibition mediated by dual PI3K/mTOR pathway targeting and genetic or direct pharmacological AKT inhibition in human glioblastoma models. J Neurochem.

[86] Chou T-C (2010). Drug combination studies and their synergy quantification using the Chou-Talalay method. Cancer Res.

