# Supplementary material for: PQR309, a dual PI3K/mTOR inhibitor, synergizes with gemcitabine by impairing the GSK-3β and STAT3/HSP60 signaling pathways to treat nasopharyngeal carcinoma
Source: Cell Death Dis. 2024 Mar 30;15(3):237. doi: 10.1038/s41419-024-06615-8 (PMC10981756; doi:10.1038/s41419-024-06615-8)
Supplement: Supplementary file 1 — Supplementary file [file 41419_2024_6615_MOESM1_ESM.docx]

**Supplementary Table 1.** **Single nucleotide polymorphism detection of the *PIK3CA* gene in nasopharyngeal carcinoma cell lines.**

| **Cell line** | **Category** | **SNP ID** | **Nucleotide change** | **Amino acid change** |
| --- | --- | --- | --- | --- |
| CNE1 | Mutant | rs3908108 | c.1837G>A | p.Gly613Ser |
|  |  | rs3865687 | c.1850G>A | p.Arg617Gln |
|  |  | rs3908109 | c.1876G>A | p.Asp626Asn |
|  |  | rs17849072 | c.1930T>C | p.Tyr644His |
|  |  | rs121913279 | c.3140A>G | p.His1047Arg |
| CNE2 | Mutant | rs121913279 | c.3140A>G | p.His1047Arg |
| S18 | Mutant | rs121913279 | c.3140A>G | p.His1047Arg |
| S26 | Mutant | rs121913279 | c.3140A>G | p.His1047Arg |
| 5-8F | Mutant | rs121913279 | c.3140A>G | p.His1047Arg |
| 6-10B | Mutant | rs121913279 | c.3140A>G | p.His1047Arg |
| SUNE1 | Mutant | rs121913279 | c.3140A>G | p.His1047Arg |
| HNE1 | Mutant | rs121913279 | c.3140A>G | p.His1047Arg |
| HONE1 | Mutant | rs121913279 | c.3140A>G | p.His1047Arg |
| TW03 | Mutant | rs121913279 | c.3140A>G | p.His1047Arg |
| HK-1 | WT | NA | NA | NA |
| C666-1 | WT | NA | NA | NA |

Abbreviation: SNP, Single nucleotide polymorphism; WT, Wild-type; NA, not available.

**Supplementary Table 2. Characteristics of all included patients**

| **Characteristics** | | **Cases (n = 161)** | **Percentage (%)** |
| --- | --- | --- | --- |
| Age (years) | Median (range) | 49 (24–76) |  |
|  | ＜60 | 137 | 85.1 |
|  | ≥ 60 | 24 | 14.9 |
| Sex | Male | 120 | 74.5 |
|  | Female | 41 | 25.5 |
| Smoking status | Non-smoker | 133 | 82.6 |
|  | Smoker | 28 | 17.4 |
| EBV status | Positive | 100 | 62.1 |
|  | Negative | 61 | 37.9 |
| Family history | Yes | 21 | 13.0 |
|  | No | 140 | 87.0 |
| Pathological type | I | 11 | 6.8 |
|  | II | 37 | 23.0 |
|  | III | 113 | 70.2 |
| Tumor stage | T1 | 21 | 13.0 |
|  | T2 | 49 | 30.4 |
|  | T3 | 65 | 40.4 |
|  | T4 | 26 | 16.1 |
| Node stage | N0 | 29 | 18.0 |
|  | N1 | 55 | 34.2 |
|  | N2 | 68 | 42.2 |
|  | N3 | 9 | 5.6 |
| Metastasis stage | M0 | 161 | 100.0 |
|  | M1 | 0 | 0 |
| Disease stage | I | 8 | 5.0 |
|  | II | 32 | 19.9 |
|  | III | 87 | 54.0 |
|  | IV | 34 | 21.1 |
| Treatment | Induction chemotherapy | 36 | 22.4 |
|  | Radiotherapy | 12 | 7.5 |
|  | Radiochemotherapy | 113 | 70.2 |
| PI3K p110α | High | 38 | 23.6 |
|  | Low | 123 | 76.4 |
| PI3K p110β | High | 78 | 48.4 |
|  | Low | 83 | 51.6 |
| DFS (months) | Median (range) | 24.2 (4.6–156.3) |  |

Abbreviations: EBV, Epstein-Barr virus; PI3K, phosphatidylinositol 3-kinase; DFS, disease-free survival.

**Supplementary Table 3. Univariate and multivariate analysis of disease-free survival.**

| Variates | Univariate |  |  | Multivariate |  |  |
| --- | --- | --- | --- | --- | --- | --- |
|  | **HR** | **95% CI** | ***P* value** | **HR** | **95% CI** | ***P* value** |
| Age  (≥ 60 *vs*. < 60 y) | 1.667 | 1.052­–2.641 | **0.030** | 1.658 | 1.043–2.635 | **0.033** |
| Sex  (Male *vs*. Female) | 0.830 | 0.571–1.207 | 0.330 |  |  |  |
| Smoking status  (Nonsmoker *vs*. Smoker) | 1.125 | 0.736-1.719 | 0.586 |  |  |  |
| Family history  (No *vs*. Yes) | 0.931 | 0.586–1.479 | 0.762 |  |  |  |
| Disease stage  (I–II *vs*. III–IV) | 0.876 | 0.611–1.256 | 0.472 |  |  |  |
| Pathological type  (I–II *vs*. III) | 1.347 | 0.938–1.933 | 0.107 |  |  |  |
| EBV status  (Negative *vs*. Positive) | 1.137 | 0.820–1.575 | 0.442 |  |  |  |
| PI3K p110α expression  (High *vs*. Low) | 1.922 | 1.327–2.785 | **0.001** | 1.841 | 1.268–2.672 | **0.001** |
| PI3K p110β expression  (High *vs*. Low) | 1.804 | 1.314–2.478 | **0.00026** | 1.811 | 1.317–2.489 | **0.00026** |

Abbreviations: HR, hazard ratio; CI, confidence interval; EBV, Epstein-Barr virus.

**Supplementary Table 4. The IC50 values of PQR309 treatment in NPC cells.**

| **Cell lines** | **IC50 of PQR309 (μM)** |
| --- | --- |
| ***PIK3CA* mutant** |  |
| CNE1 | 0.6002 |
| HNE1 | 0.1102 |
| HONE-1 | 0.0120 |
| SUNE1 | 0.02271 |
| 6-10B | 0.05437 |
| 5-8F | 0.07331 |
| S18 | 0.02547 |
| S26 | 0.01105 |
| CNE2 | 0.01372 |
| CNE2-EBV | 0.03003 |
| TW03 | 0.008617 |
| TW03-EBV | 0.02051 |
| ***PIK3CA* wild-type** |  |
| HK-1 | 0.01342 |
| C666-1 | 0.01495 |

**Supplementary Table 5. The IC50 values of BYL719 or GSK2636771 treatment in NPC cells.**

| **Cell lines** | **IC50 of BYL719 (μM)** | **IC50 of GSK2636771 (μM)** |
| --- | --- | --- |
| ***PIK3CA* mutant** |  |  |
| CNE1 | 23.81 | 41.69 |
| HNE1 | 99.05 | 38.65 |
| HONE-1 | 33.12 | 15.54 |
| SUNE1 | 15.93 | 179.5 |
| 6-10B | 1.334 | 32.16 |
| 5-8F | 58.31 | 27.46 |
| S18 | 98.03 | 99.05 |
| S26 | 7.907 | 17.13 |
| CNE2 | 20.82 | 20.31 |
| TW03 | 7.255 | 25.15 |
| ***PIK3CA* wild-type** |  |  |
| HK-1 | 8.512 | 57.82 |
| C666-1 | 24.85 | 69.55 |

**Supplementary Table 6. Drug combination indices of gemcitabine and PQR309 or BYL719, GSK2636771 against CNE1 and HNE1 cells.**

|  | **PQR309** | | | **BYL719** | | | **GSK2636771** | | |
| --- | --- | --- | --- | --- | --- | --- | --- | --- | --- |
| CI | ED50 | ED75 | ED90 | ED50 | ED75 | ED90 | ED50 | ED75 | ED90 |
| CNE1 | 0.39771 | 0.51890 | 0.78244 | 0.37352 | 0.54984 | 0.82771 | 1.01264 | 1.02663 | 1.07417 |
| HNE1 | 0.55211 | 0.59394 | 0.83901 | 0.48800 | 0.29342 | 0.17670 | 1.05231 | 1.05752 | 1.06617 |

Abbreviations: CI, combination index. ED: effective dose.

**Supplementary Table 7. List of antibodies used for immunohistochemistry and western blotting analyses.**

| **Antibodies** | **Code** |
| --- | --- |
| **Immunohistochemistry** |  |
| PI3 Kinase p110 α | #ab135384 |
| PI3 Kinase p110 β | #ab151549 |
| Ki67 | #ab15580 |
| Phospho STAT3 | #ab76315 |
| Phospho GSK-3β | #9323 |
| HSP60 | #12165 |
| **Western blotting** |  |
| Caspase3 | #9662 |
| Cleaved Caspase3 | #9664 |
| Caspase9 | #9502 |
| Cleaved Caspase9 | #9505 |
| N-Cadherin | #13116 |
| E-Cadherin | #3195 |
| β-Catenin | #8480 |
| Vimentin | #5741 |
| STAT3 | #12640 |
| Phospho STAT3 | #9145 |
| HSP60 | #12165 |
| GSK-3α | #4818 |
| Phospho GSK-3α | #9316S |
| GSK-3β | #12456S |
| Phospho GSK-3β | #9323 |
| GAPDH | #5174 |
| β-Actin | #8457 |
| β-tubulin | #2128 |
| α-Tubulin | #2125 |
| PI3 Kinase p110 α | #4249S |
| PI3 Kinase p110 β | #3011S |
| PIK3CA | #ab40776 |
| PIK3CB | #ab151549 |
| Phospho mTOR | #2974T |
| mTOR | #2972 |
| Phospho AKT | #4060S |
| AKT | #9272 |
| Phospho S6 | #4858S |
| S6 | #2317 |
| Phospho 4E-BP1 | #2855 |
| 4E-BP1 | #9644 |
| Phospho S6K | #9234 |
| S6K | #34475 |
| Phospho FOXO1 | #9461 |
| FOXO1 | #2880 |
| Phospho FOXO3a | #9465 |
| FOXO3a | #12829 |
| Phospho FOXO4 | #abs146185 |
| FOXO4 | #9472 |

**Supplementary Table 8. siRNA of of PI3K p110α and p110β**

| **Gene** | **Scramble siRNA** | **Target siRNA** |
| --- | --- | --- |
| ***PI3K p110α*** | CCCTAAGATCCGATGGTAATAATTA | CCCAAGAATCCTAGTAGAATGTTTA |
| ***PI3K p110α*** | CGGCGTGACTGTGTGTAATTCAGAT | CGGCATGCCAGTGTGTGAATTTGAT |
| ***PI3K p110β*** | GGTAAACGGGATACGCTCTAT | GACTGCGCACTTGGAATAGTA |
| ***PI3K p110β*** | GACAATGCAACGGACTGATAT | GATGAAACACGAAGACTCTGT |

**
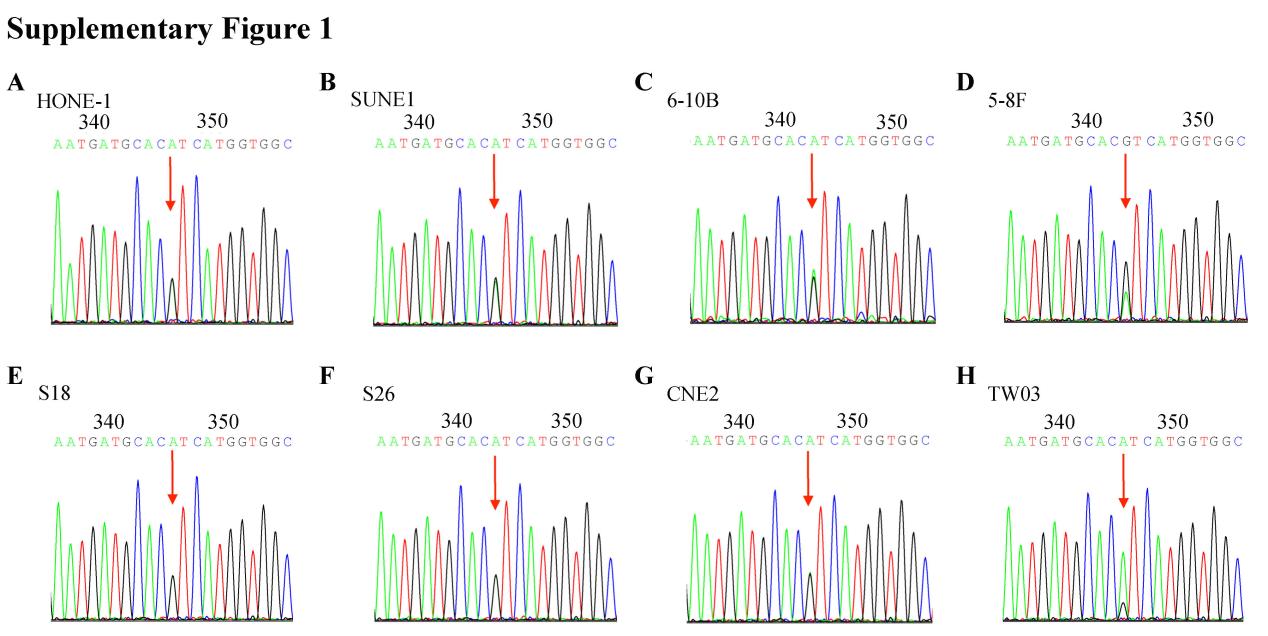
**

**Supplementary Figure 1.** Gene analysis of *PIK3CA* in NPC cells lines. (A-H) *PIK3CA* mutation frequency was high in most NPC cell lines (HONE-1, SUNE1, 6-10B, 5-8F, S18, S26, CNE2 and TW03). NPC, nasopharyngeal carcinoma; *PIK3CA*, phosphatidylinositol-4,5-bisphosphate 3-kinase catalytic subunit alpha gene;

**
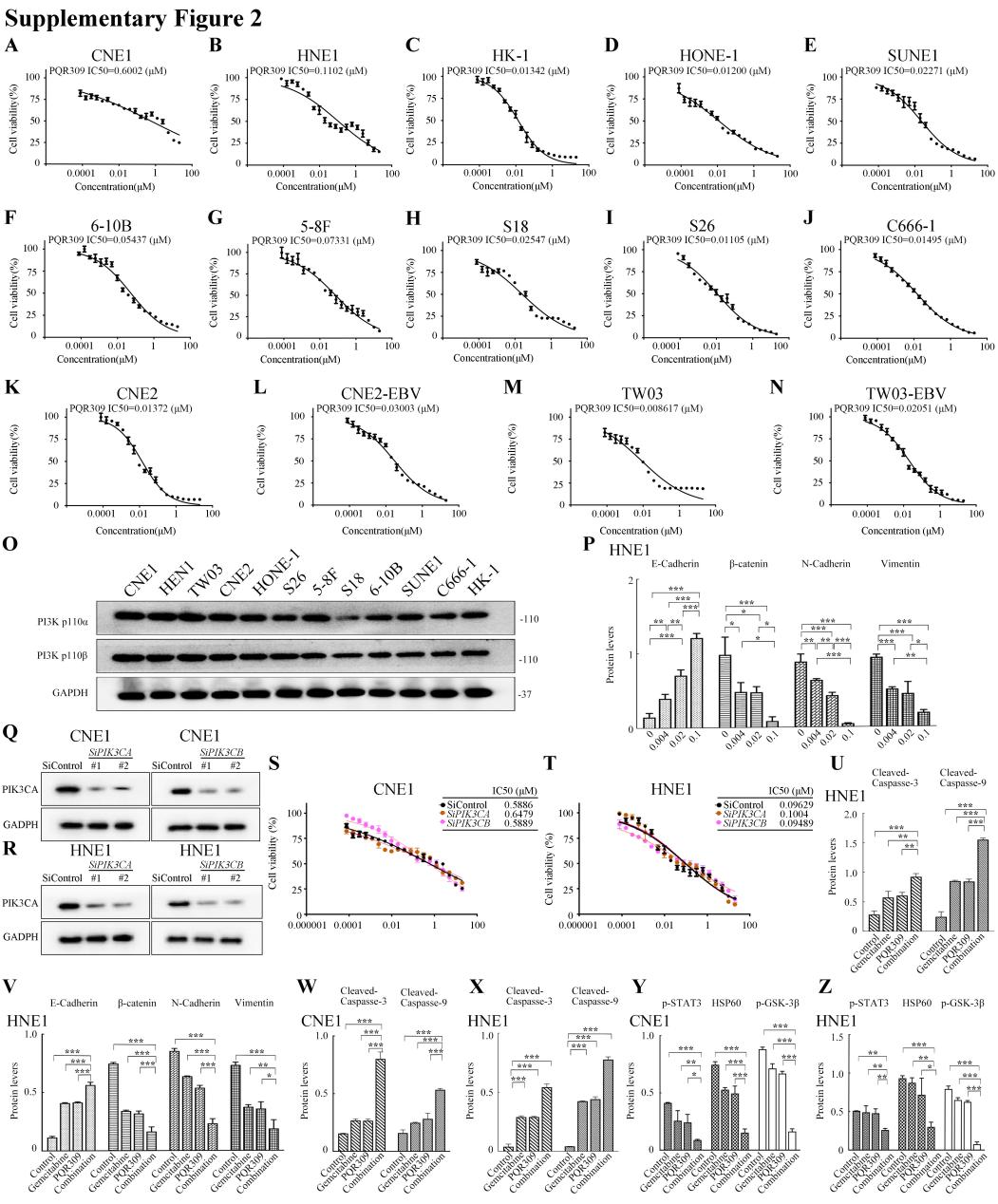
**

**Supplementary Figure 2.** PQR309 and gemcitabine can effectively inhibit NPC tumor activity *in vivo* and *in vitro*. (A-N) Various concentrations of PQR309 were used to treat 14 NPC cell lines (CNE1, HNE1, HK-1, HONE-1, SUNE1, 6-10B, 5-8F, S18, S26, C666-1, CNE2, CNE2-EBV, TW03, and TW03-EBV) for 72 h. Cell proliferation was determined using a CCK-8 assay in triplicate. The IC50 values of PQR309 in NPC cells were also analyzed. (O) Western blotting assessment of the expression of PI3K α and PI3K β in 12 NPC cell lines (CNE1, HNE1, TW03, CNE2, HONE-1, S26, 5-8F, S18, 6-10B, SUNE1, C666-1, HK-1). (P) HNE1 cells were exposed to different concentrations of PQR309 for 24–48 h. Western blotting was applied to examine the expression of EMT markers (E-cadherin, β-catenin, N-cadherin, and Vimentin). (Q-R) Western blotting to confirmed the downregulation of PI3K p110αor p110βin CNE1 and HNE1 cells with siRNA to silence these proteins. (S-T) the sensitivity to PQR309 remained unchanged despite siRNA targeting *PI3K p110α* or *p110β* by CCK8 assay*.* (U) Western blotting was applied to detect cleaved caspase-3 and cleaved caspase-9 levels as indicators of apoptotic cell death in HNE1 cells. (V) HNE1 cells were exposed to different treatments for 24–48 h, including PQR309, gemcitabine, or their combination. Western blotting was applied to examine the expression of EMT markers (E-cadherin, β-catenin, N-cadherin, Vimentin). (W-Z) Western blotting of cleaved caspase-3, cleaved caspase-9, STAT3, p-STAT3, HSP60, p-GSK-3β and GSK-3β from CNE1 and HNE1 xenograft tumors from the four different treatment groups. **P* < 0.05, ***P* < 0.01, ****P* < 0.001. p-STAT3, phosphorylated signal transducer and activator of transcription 3; HSP60, heat shock protein 60; p-GSK-3β, phosphorylated glycogen synthase kinase-3β; shRNA, short hairpin RNA.


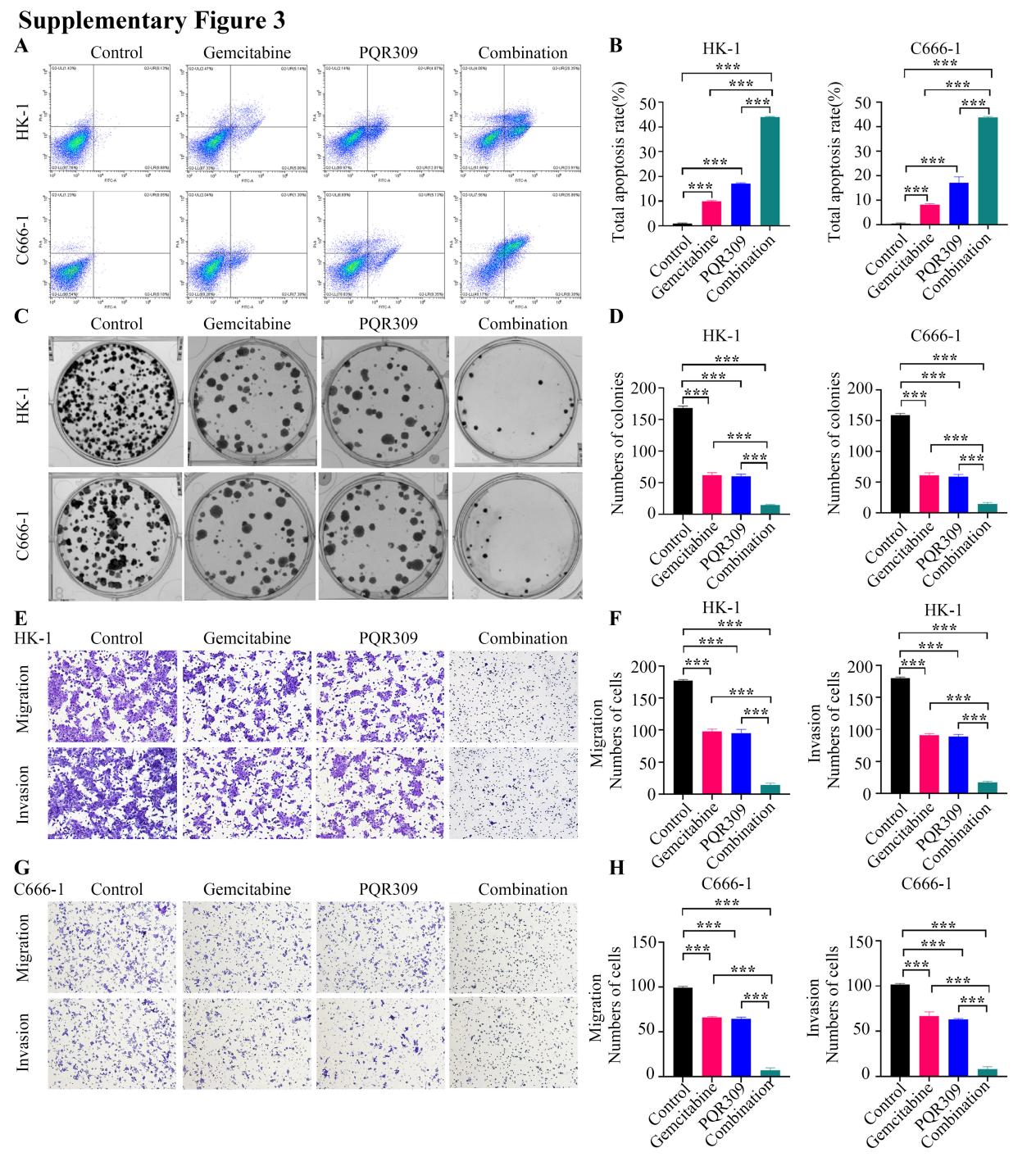


**Supplementary Figure 3。**(A-B) The apoptotic rate of HK-1 and C666-1 cells after different treatment, involving control, gemcitabine, PQR309, and their combination, were tested by the Annexin V/PI assay. (C-D) Typical images and enumeration of HK-1 and C666-1 cells treated with control, gemcitabine, PQR309, and their combinationon after crystal violet staining. (E-H) Invasion and migration assays of HK-1 and C666-1 cells in the four groups. All **P* < 0.05, ***P* < 0.01, ****P* < 0.001.

**
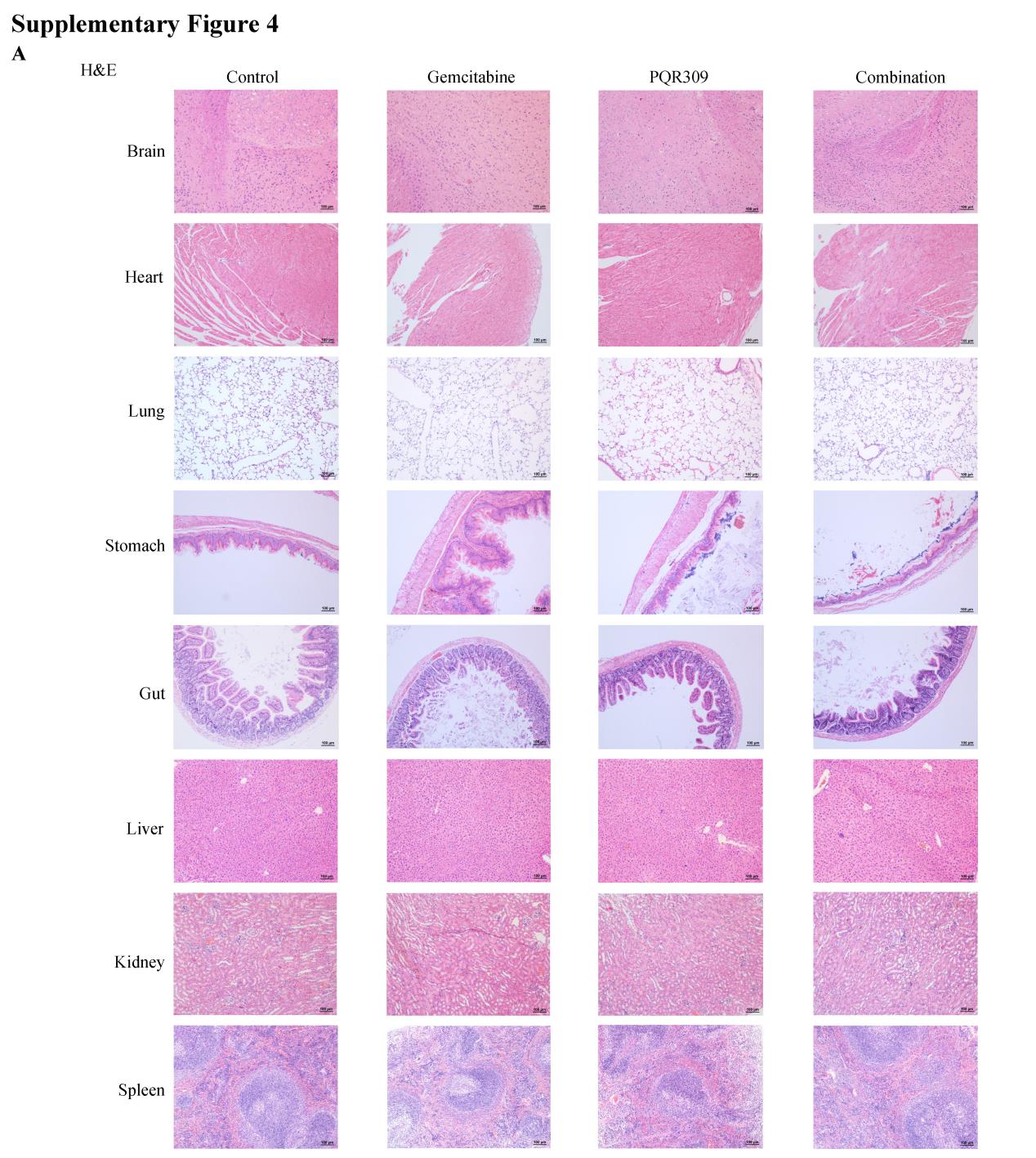
**

**Supplementary Figure 4.** HE staining of the internal organs of mice during the experiment. HE, hematoxylin and eosin.


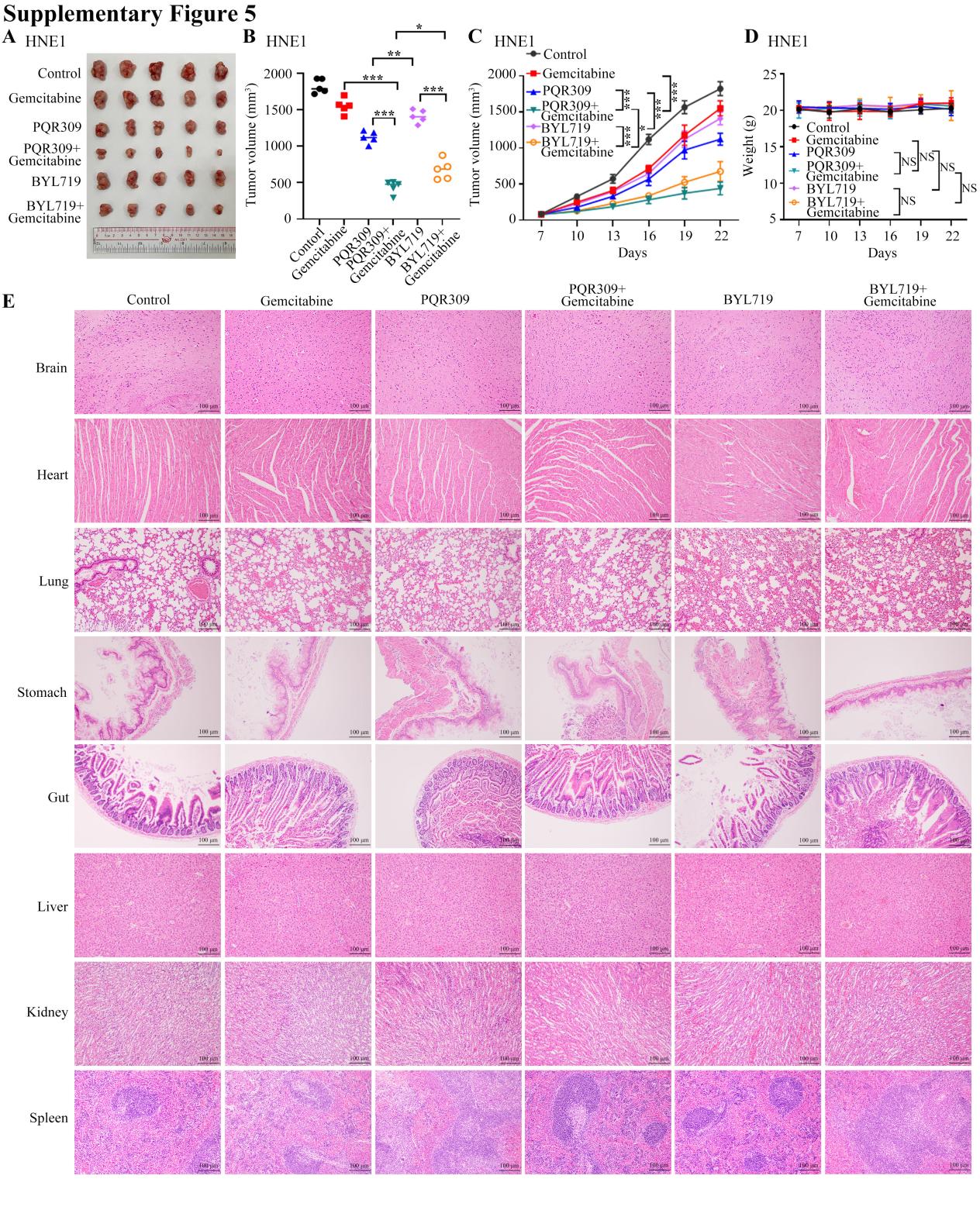


**Supplementary Figure 5.** (A-D) Images and volumes of tumors, and weights of nude mices with HNE1 xenograft tumors were harvested at the end of the experiment. (E) HE staining of the internal organs of mice exposed to different treatments. All **P* < 0.05, ***P* < 0.01, ****P* < 0.001.


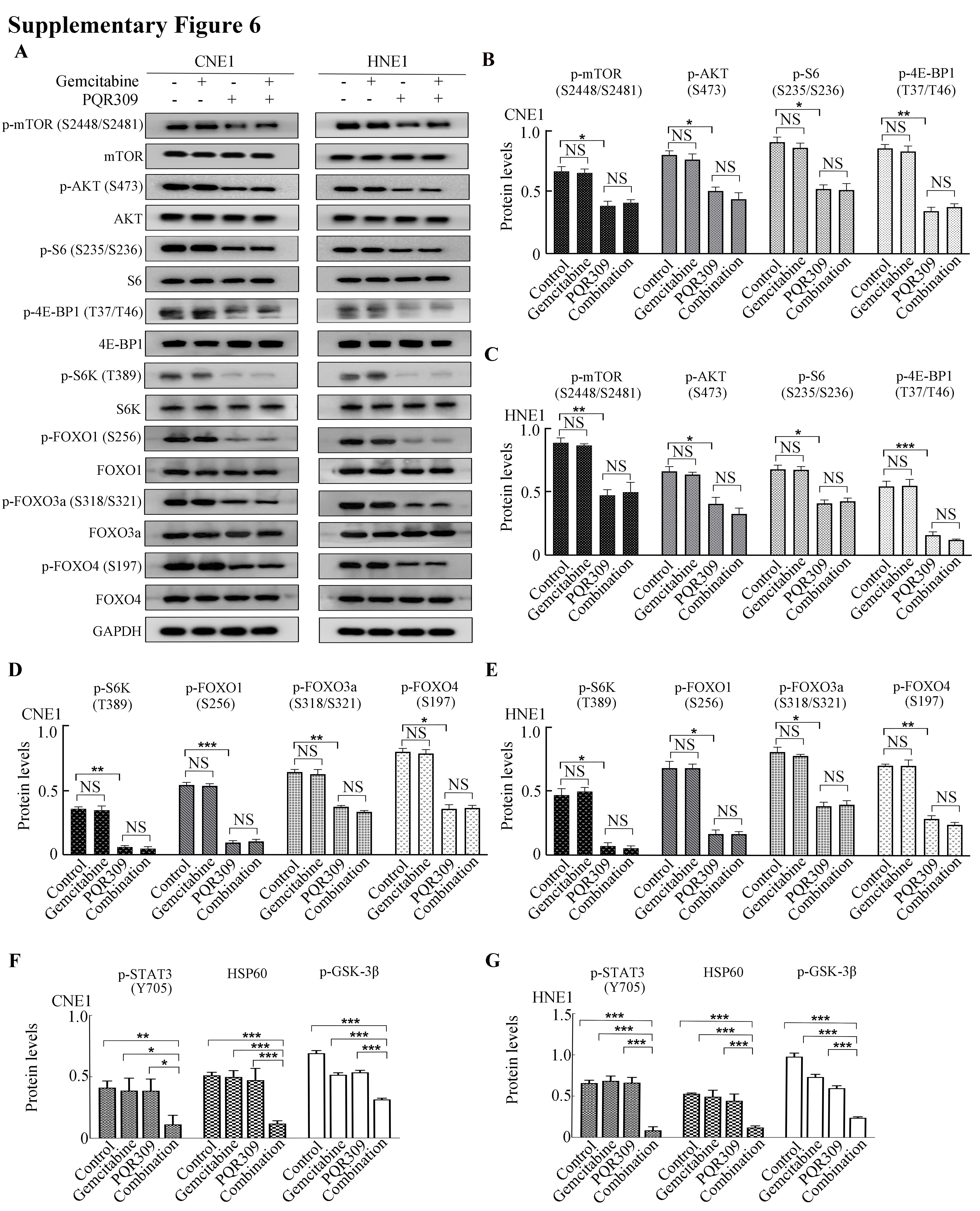


**Supplementary Figure 6.** (A-E) Western blot analysis of PI3K/mTOR pathway in CNE1 and HNE1 cells. (F-G) Western blotting of p-STAT3, HSP60, p-GSK-3β from CNE1 and HNE1 cells the four different treatment groups.


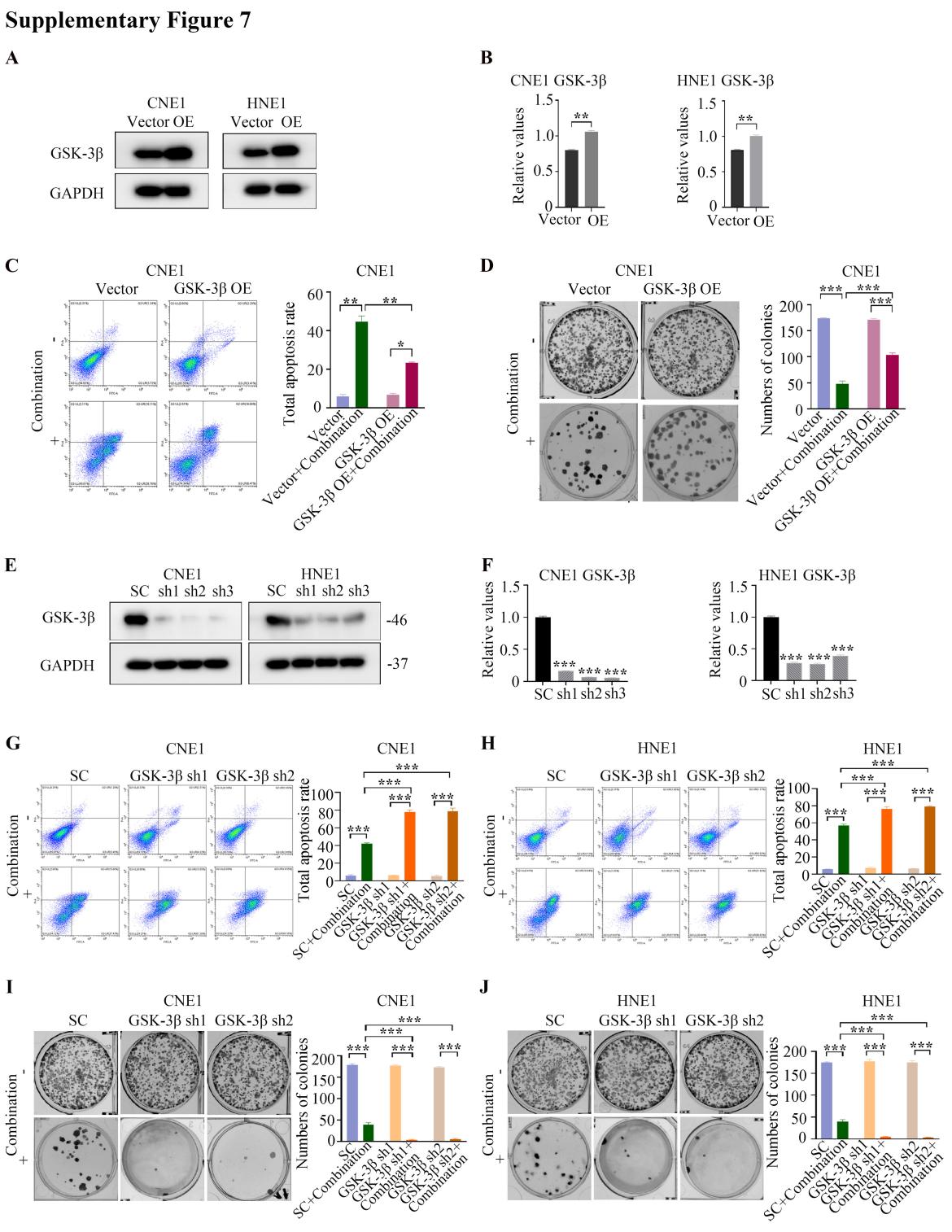


**Supplementary Figure 7.** (A-B) Western blotting analysis of GSK-3β in GSK-3β overexpressed NPC cells. (C) Annexin V/PI analysis of GSK-3β overexpressed CNE1 cells treated with PQR309 and gemcitabine. following 24 h of exposure to different treatments. (D) After pretreatment with PQR309 and gemcitabine in GSK-3β overexpressed CNE1 cells, number of colonies was examined. (E-F) After the transfection of CNE1 and HNE1 cells with the GSK-3β shRNAs, protein level of GSK-3β in the indicated cells was examined by western blotting. (G-J) After pretreatment with PQR309 and gemcitabine in GSK-3β knockdown CNE1 and HNE1 cells, percentage of cell apoptosis and number of colonies of the indicated cells were recorded. **P* < 0.05, ***P* < 0.01, ****P* < 0.001. GSK-3β, glycogen synthase kinase-3β; shRNA, short hairpin RNA.


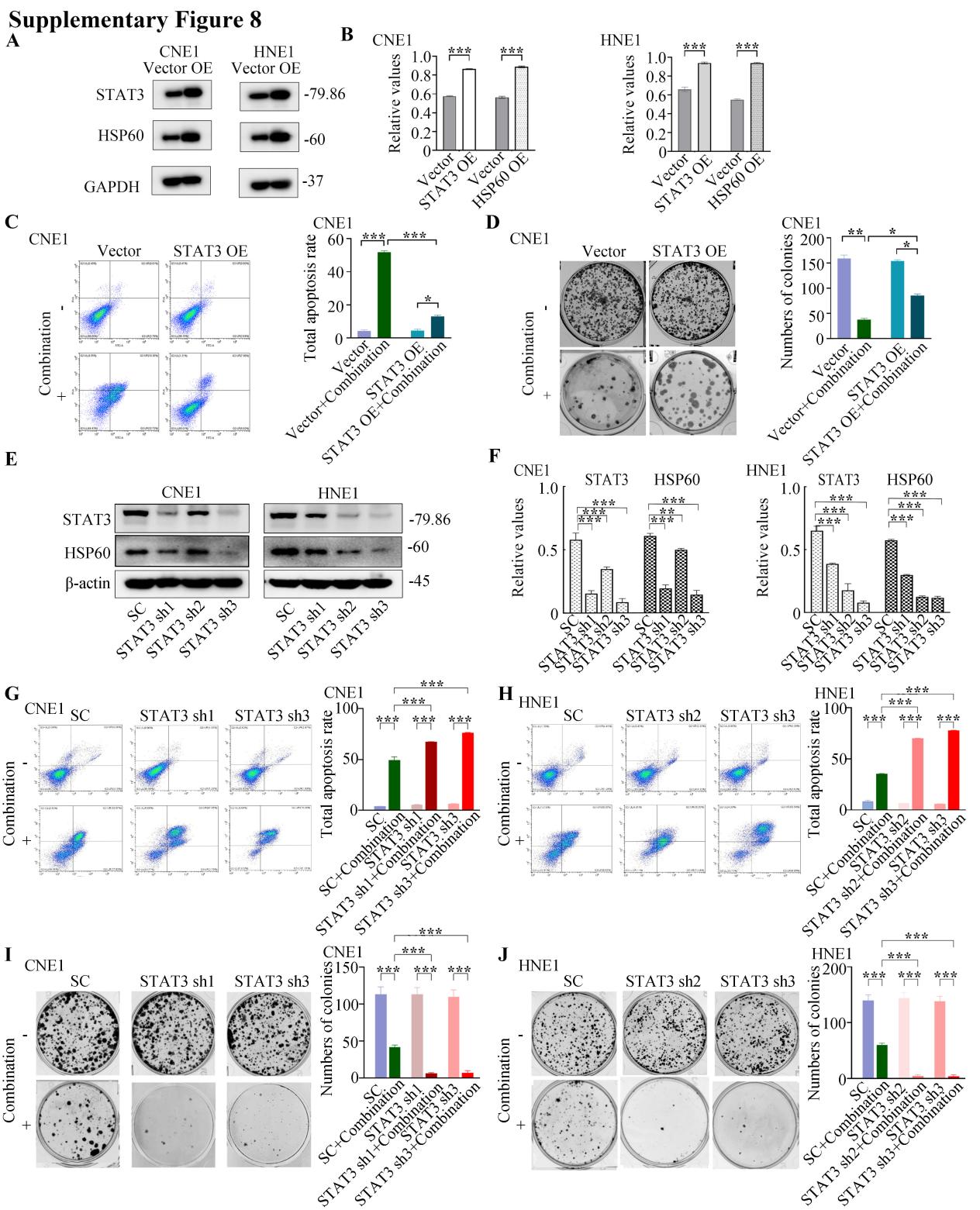


**Supplementary Figure 8.** (A-B) *STAT3*-overexpressing NPC cells were established, then protein level of *STAT3* and HSP60 in the indicated groups was detected with western blot. (C-D) The combination of PQR309 and gemcitabine were used to treat *STAT3*-overexpressing CNE1 cells, the number of colonies stained by crystal violet and the apoptotic rate was examined. (E-F) Protein expression of STAT3 and HSP60 were analyzed by Western blotting in *STAT3* knockdown NPC cells. (G-H) FACS analysis of annexin V/PI staining of *STAT3* knockdown CNE1 and HNE1 cells treated with PQR309 and gemcitabine. (I-J) Representative images and quantification of colony number of *STAT3* knockdown CNE1 and HNE1 cells with the indicated combined treatment. **P* < 0.05, ***P* < 0.01, ****P* < 0.001. STAT3, signal transducer and activator of transcription 3; HSP60, heat shock protein 60.


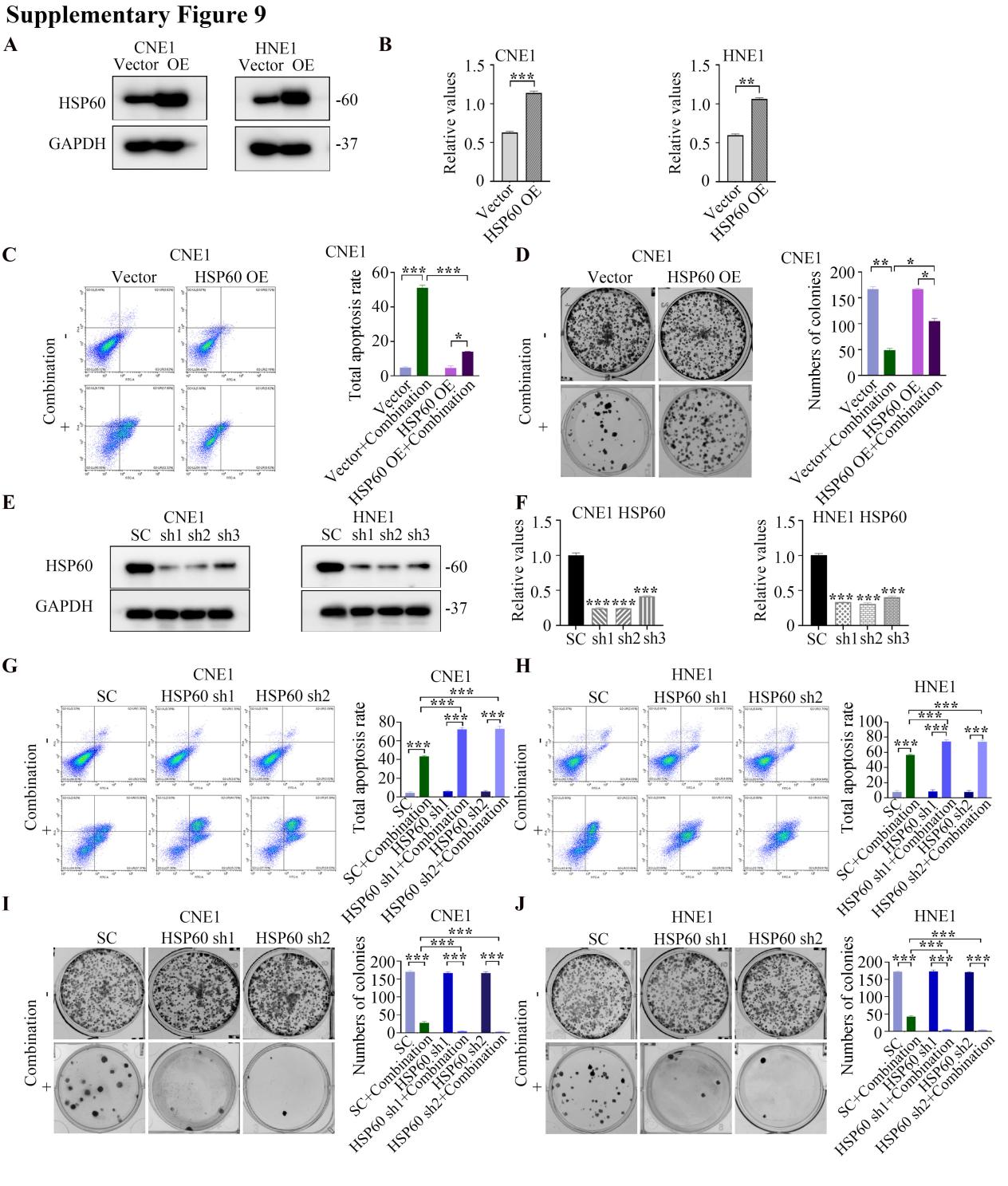


**Supplementary Figure 9.** (A-B) Western blot of HSP60 protein in *HSP60* overexpressed NPC cells. (C) *HSP60* overexpressed NPC cells undergo apoptosis upon the treatment of PQR309 and gemcitabine by flow cytometry. (D) Colonies were visualized by Giemsa staining in *HSP60* overexpressed CNE1 cells after the treatment of PQR309 and gemcitabine. (E-F) Western blot of HSP60 protein in *HSP60* knockdown NPC cells. (G-H) Flow cytometry was performed to assess apoptotic *HSP60* knockdown NPC cells after the treatment of PQR309 and gemcitabine. (I-J) The numeber of colonies was examined in *HSP60* knockdown NPC cells exposed to the combined treatment of PQR309 and gemcitabine. **P* < 0.05, ***P* < 0.01, ****P* < 0.001. HSP60, heat shock protein 60.
