# Supplementary material for: PQR309, a dual PI3K/mTOR inhibitor, synergizes with gemcitabine by impairing the GSK-3β and STAT3/HSP60 signaling pathways to treat nasopharyngeal carcinoma
Source: Cell Death Dis. 2024 Mar 30;15(3):237. doi: 10.1038/s41419-024-06615-8 (PMC10981756; doi:10.1038/s41419-024-06615-8)

Figure 2E

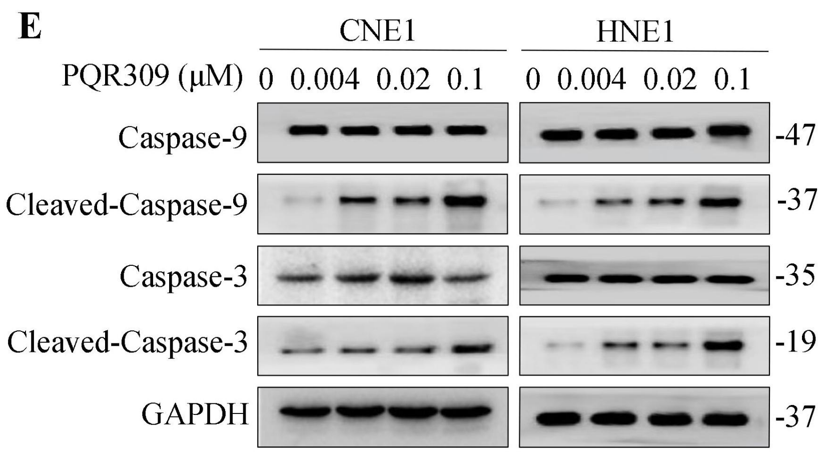
E
CNE1
HNE1
PQR309 (μM) 0 0.004 0.02 0.1 0 0.004 0.02 0.1
Caspase-9
Cleaved-Caspase-9
Caspase-3
Cleaved-Caspase-3
GAPDH
-47
-37
-35
-19
-37

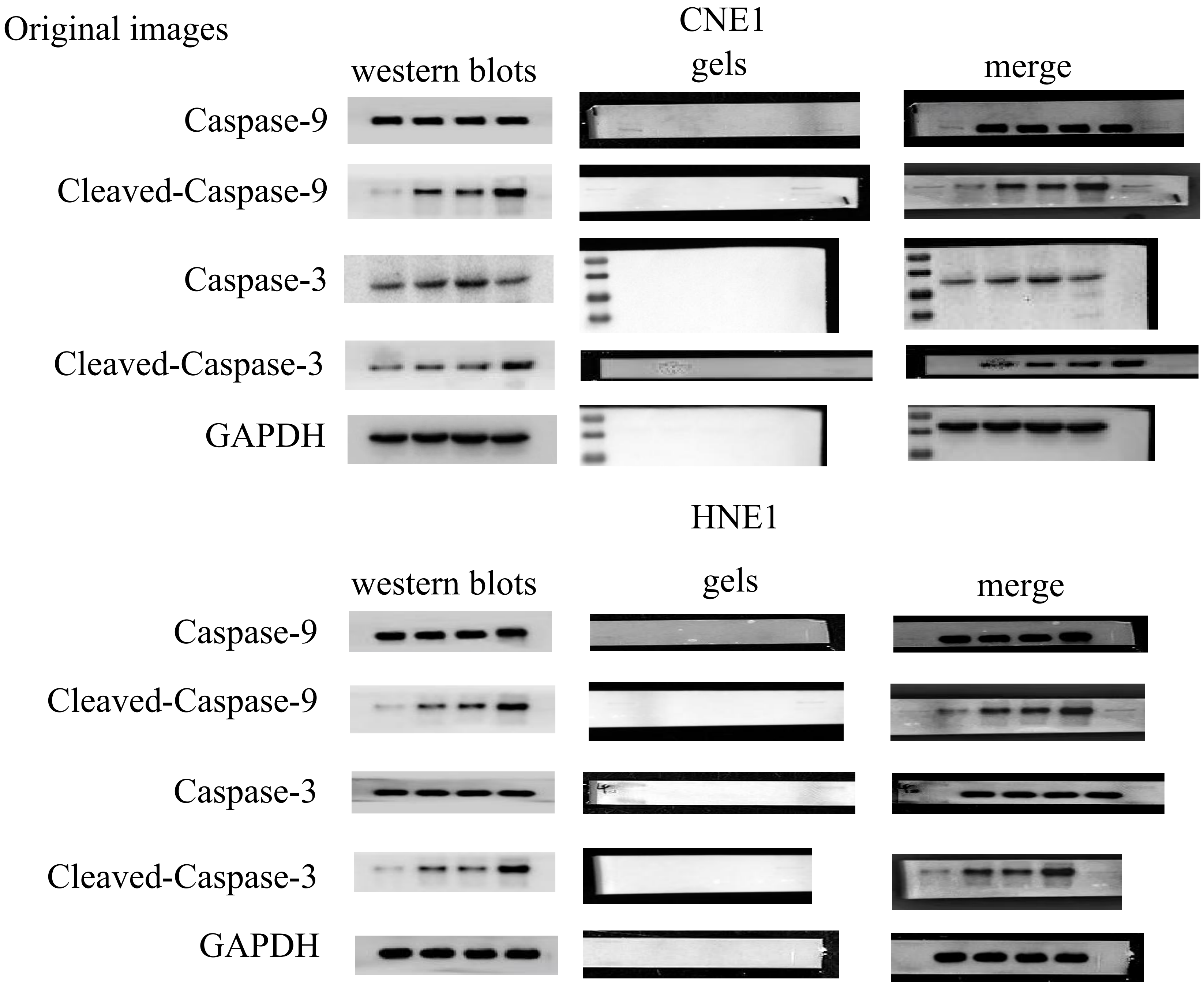
Original images
CNE1
western blots
gels
merge
Caspase-9
Cleaved-Caspase-9
Caspase-3
Cleaved-Caspase-3
GAPDH
HNE1
western blots
gels
merge
Caspase-9
Cleaved-Caspase-9
Caspase-3
Cleaved-Caspase-3
GAPDH

Figure 2K

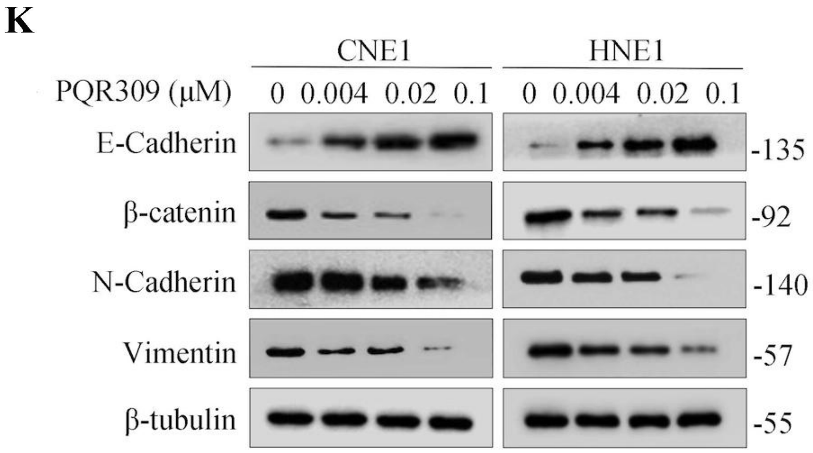
K
CNE1
HNE1
PQR309 (μM)
0 0.004 0.02 0.1
0 0.004 0.02 0.1
E-Cadherin
β-catenin
N-Cadherin
Vimentin
β-tubulin
-135
-92
-140
-57
-55

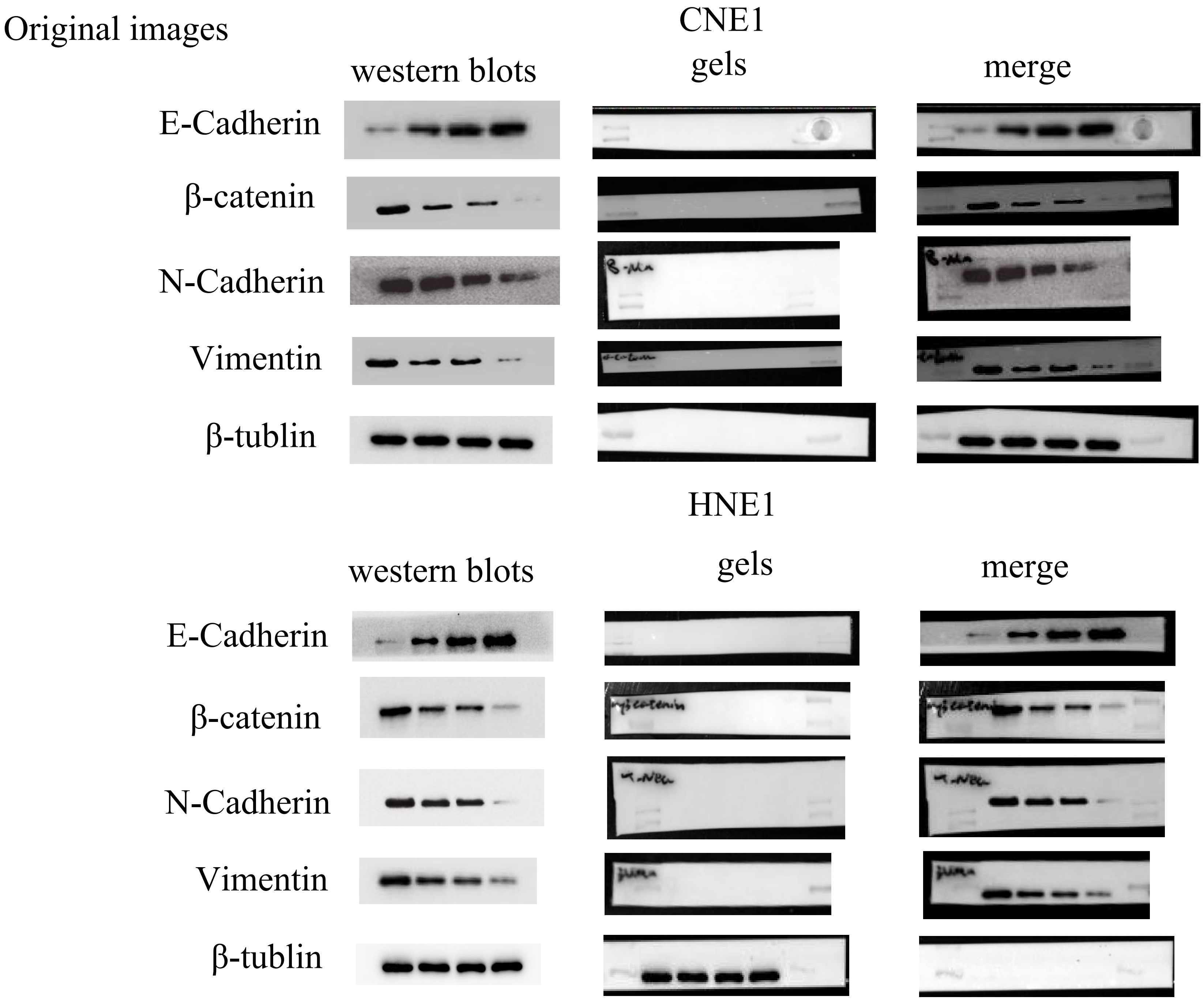
Original images
CNE1
western blots
gels
merge
E-Cadherin
β-catenin
N-Cadherin
Vimentin
β-tublin
HNE1
western blots
gels
merge
E-Cadherin
β-catenin
N-Cadherin
Vimentin
β-tublin

Figure 3F

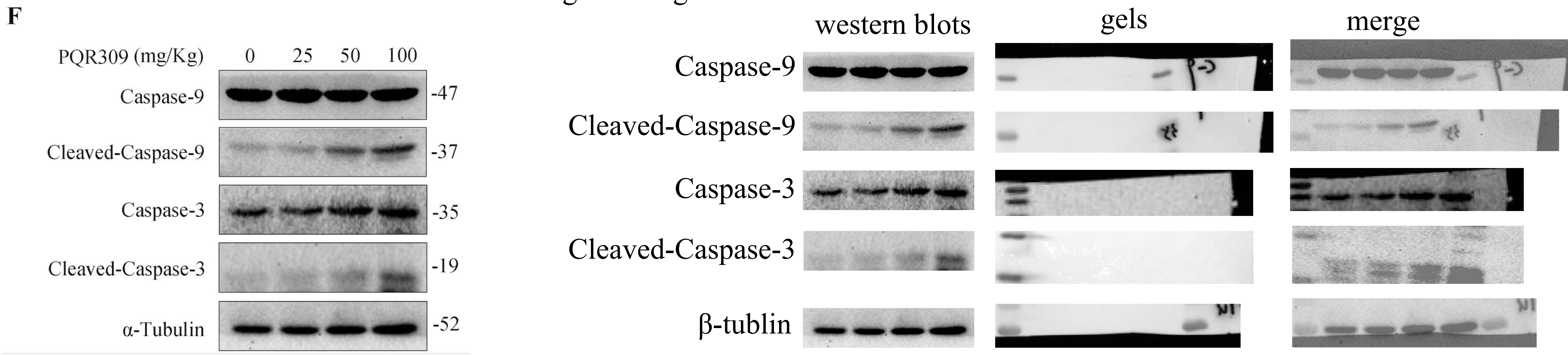
F
PQR309 (mg/Kg)
0
25
50
100
Caspase-9
-47
Cleaved-Caspase-9
-37
Caspase-3
-35
Cleaved-Caspase-3
-19
α-Tubulin
-52
Original images
western blots
gels
merge
Caspase-9
Cleaved-Caspase-9
Caspase-3
Cleaved-Caspase-3
β-tublin

Figure 4K

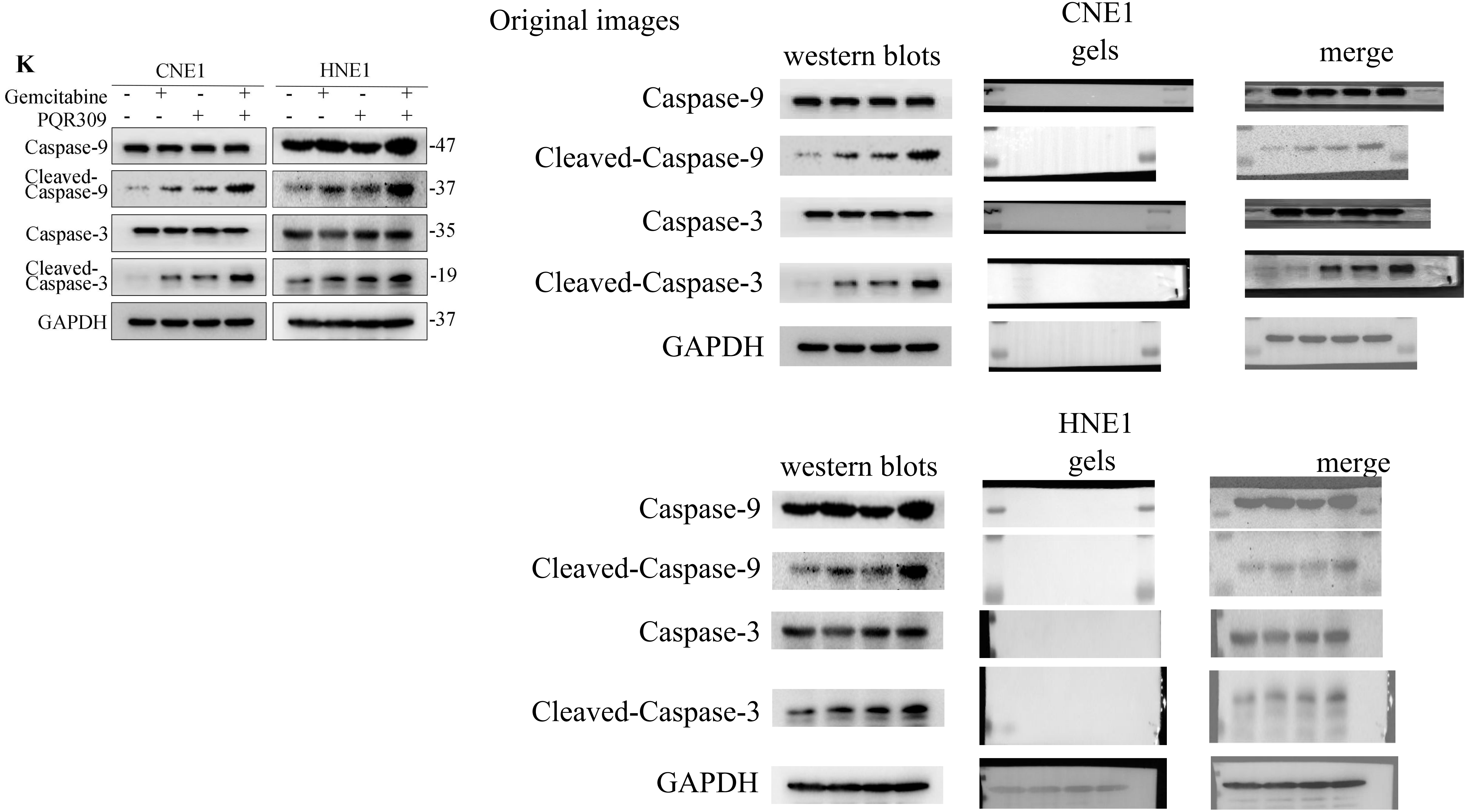
K
CNE1
HNE1
Gemcitabine - + - + - + - +
PQR309 - - + + - - + +
Caspase-9
Cleaved-Caspase-9
Caspase-3
Cleaved-Caspase-3
GAPDH
-47
-37
-35
-19
-37
Original images
CNE1
western blots
gels
merge
Caspase-9
Cleaved-Caspase-9
Caspase-3
Cleaved-Caspase-3
GAPDH
HNE1
western blots
gels
merge
Caspase-9
Cleaved-Caspase-9
Caspase-3
Cleaved-Caspase-3
GAPDH

Figure 4M

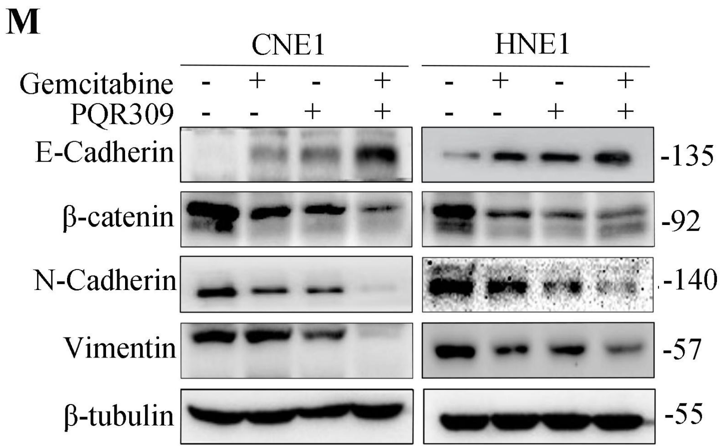

Original images

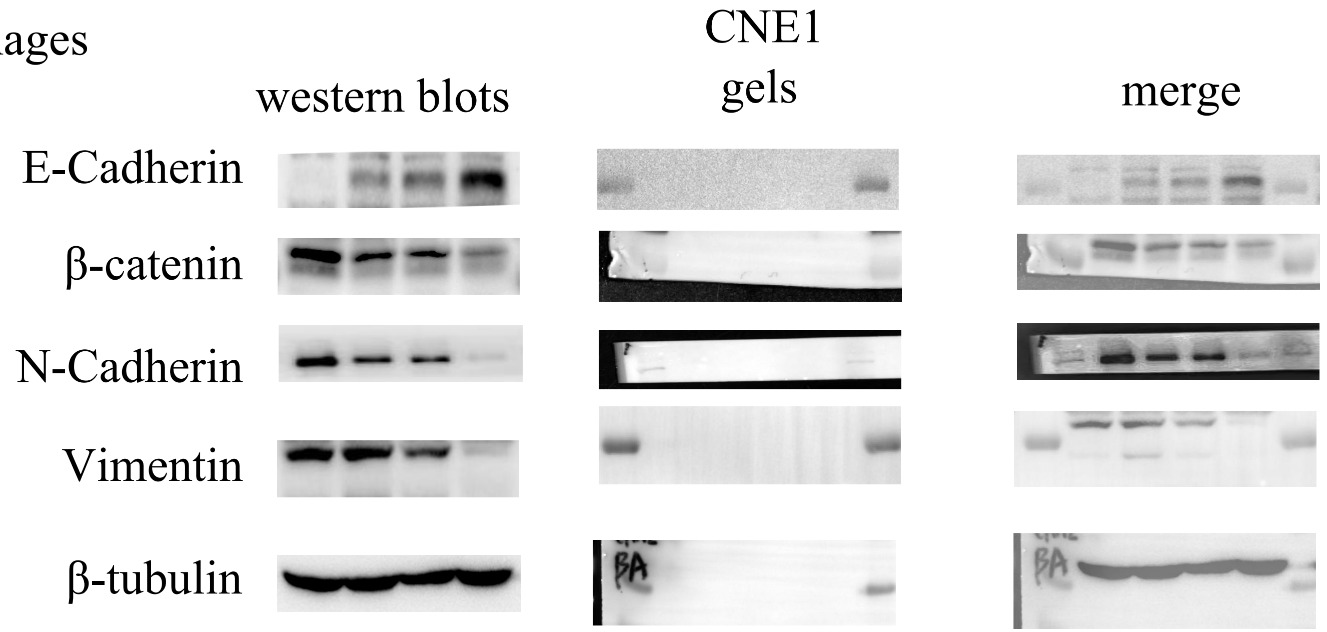

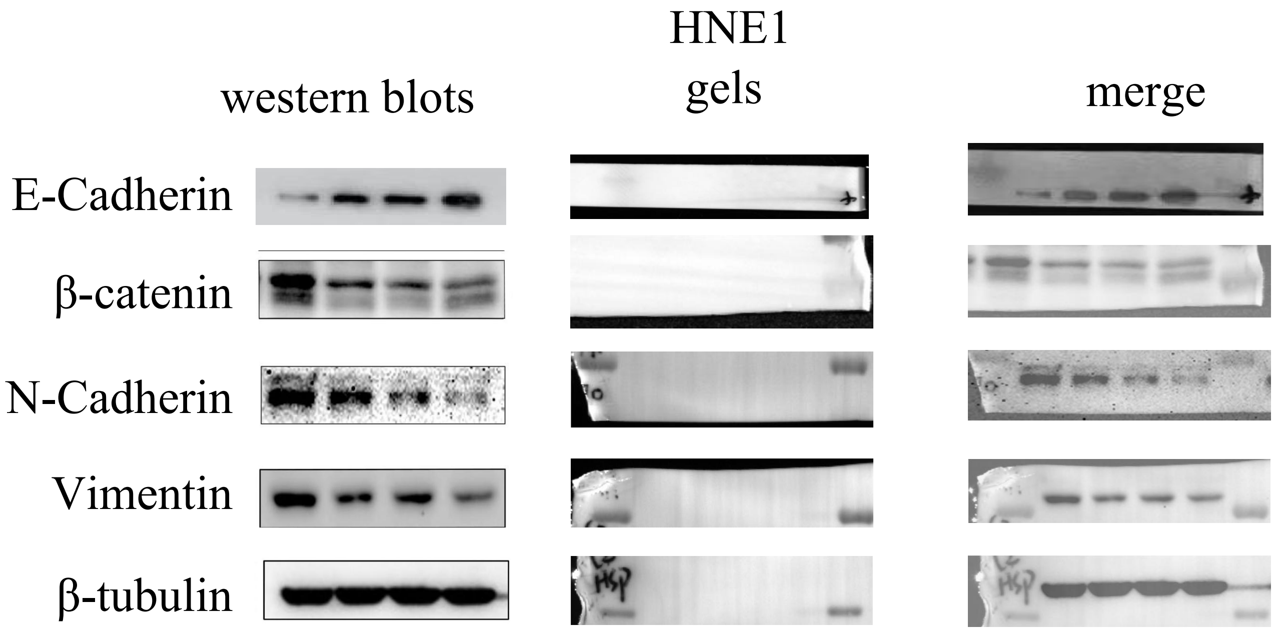

Figure 5K

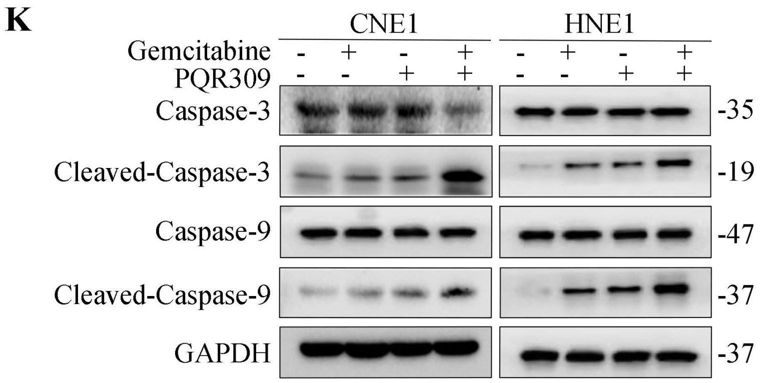
K
CNE1
HNE1
Gemcitabine - + - + - + - +
PQR309 - - + + - - + +
Caspase-3
-35
Cleaved-Caspase-3
-19
Caspase-9
-47
Cleaved-Caspase-9
-37
GAPDH
-37

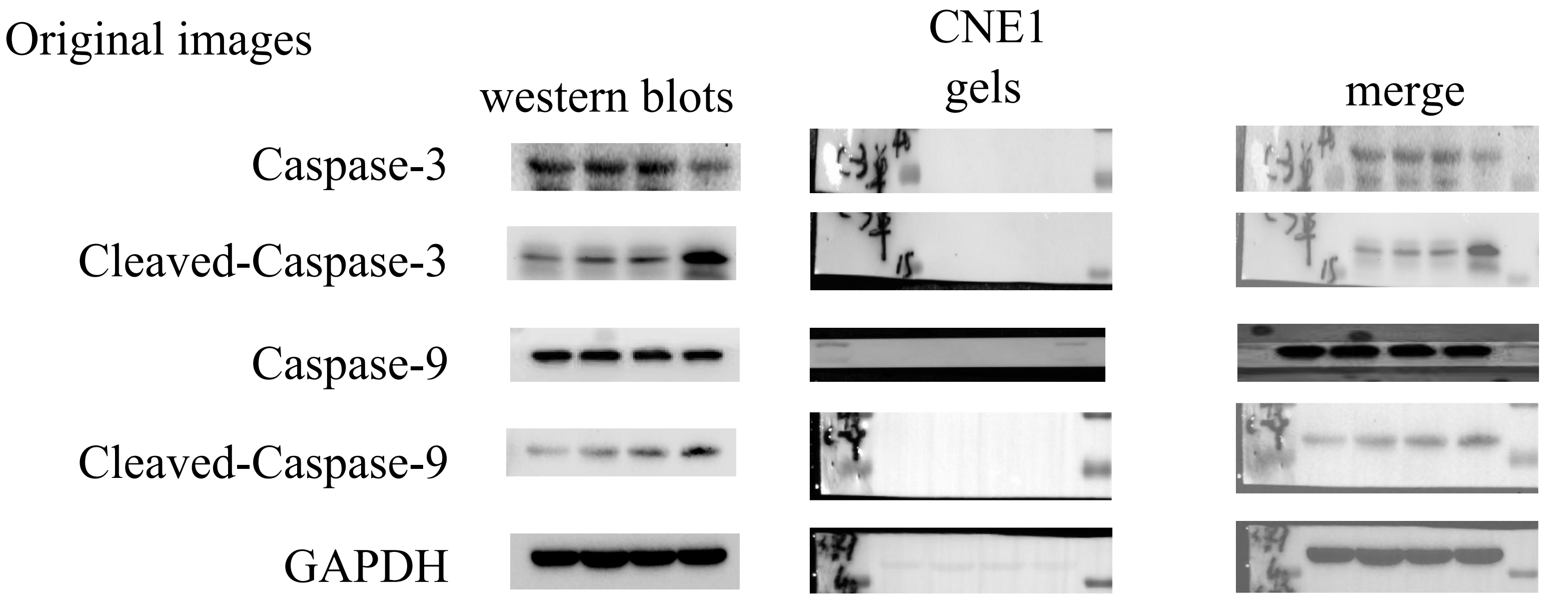
Original images
CNE1
western blots
gels
merge
Caspase-3
Cleaved-Caspase-3
Caspase-9
Cleaved-Caspase-9
GAPDH

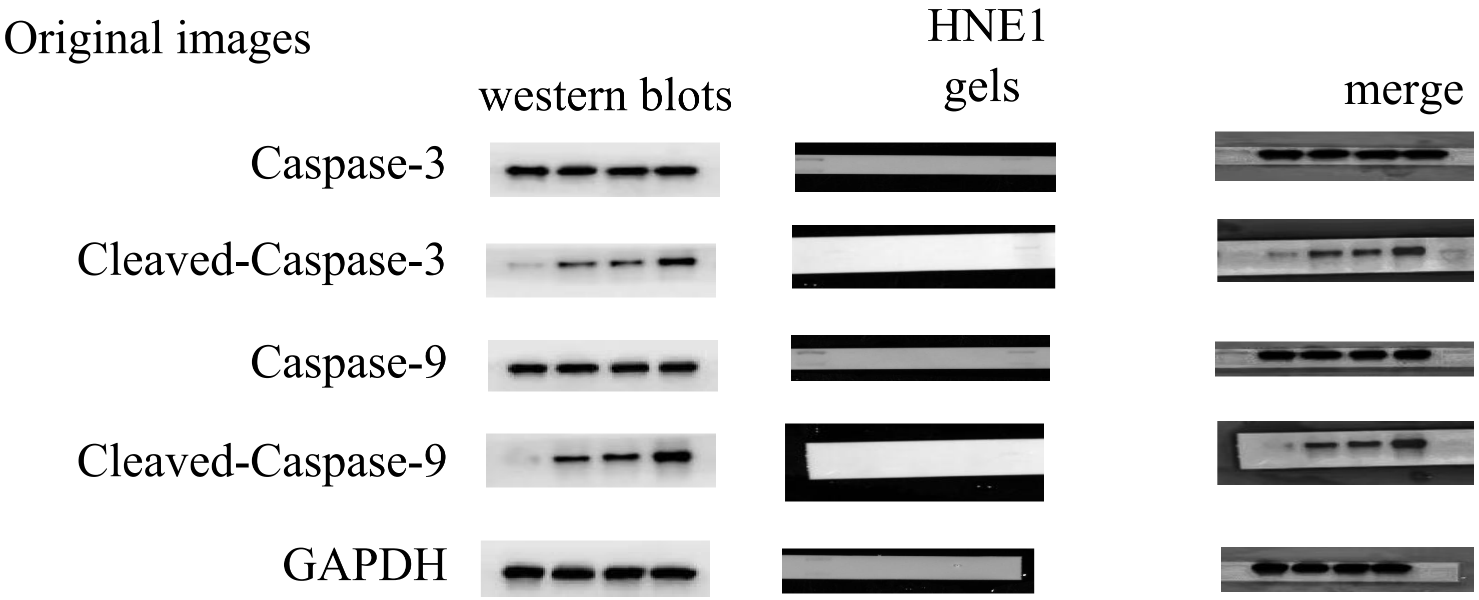
Original images
HNE1
western blots
gels
merge
Caspase-3
Cleaved-Caspase-3
Caspase-9
Cleaved-Caspase-9
GAPDH

Figure 5L

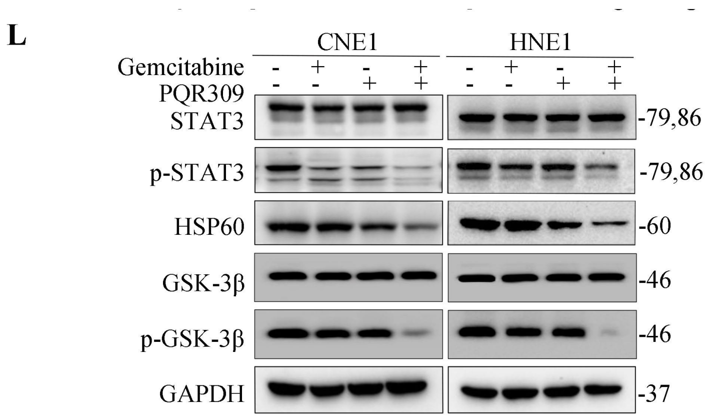

Original images

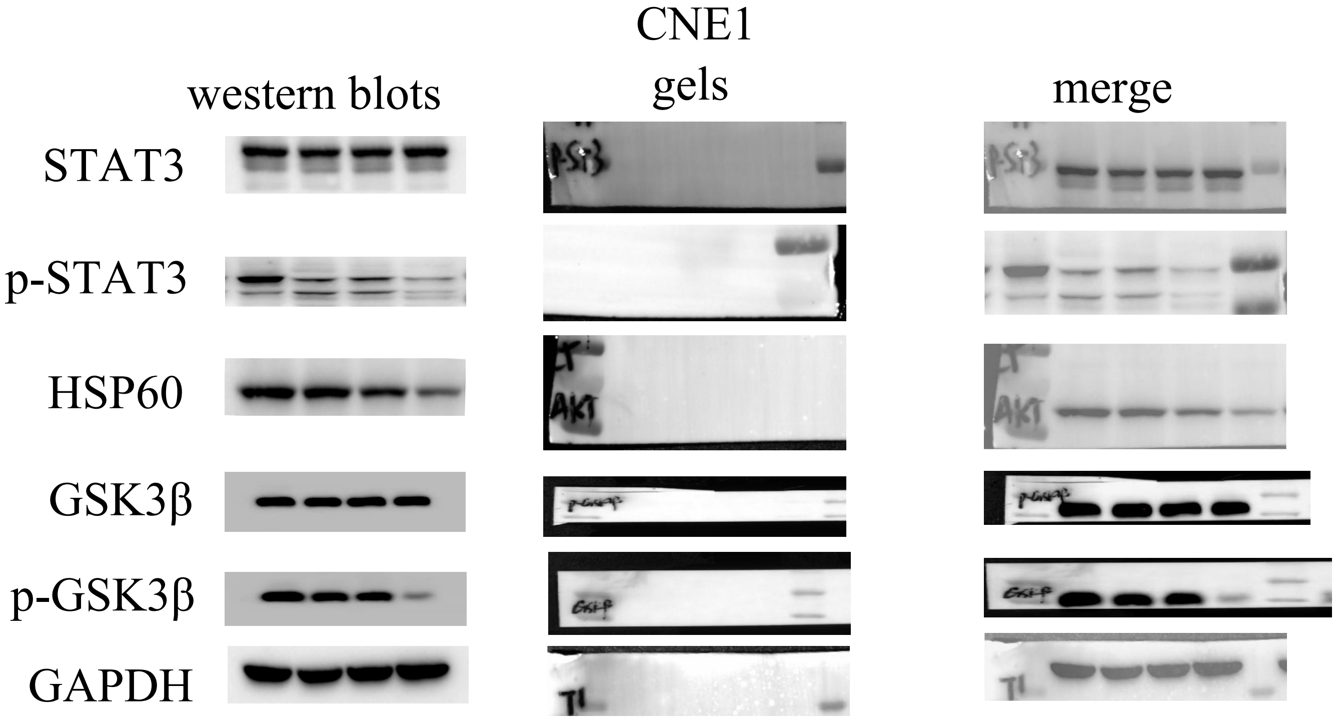

Figure 5L

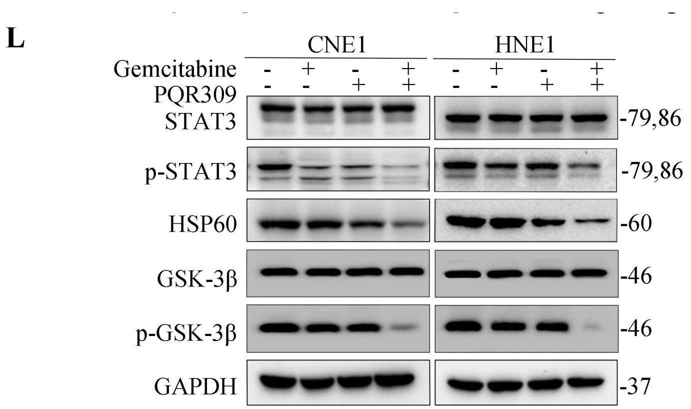
L
CNE1
HNE1
Gemcitabine
PQR309
STAT3
p-STAT3
HSP60
GSK-3β
p-GSK-3β
GAPDH
-79,86
-79,86
-60
-46
-46
-37

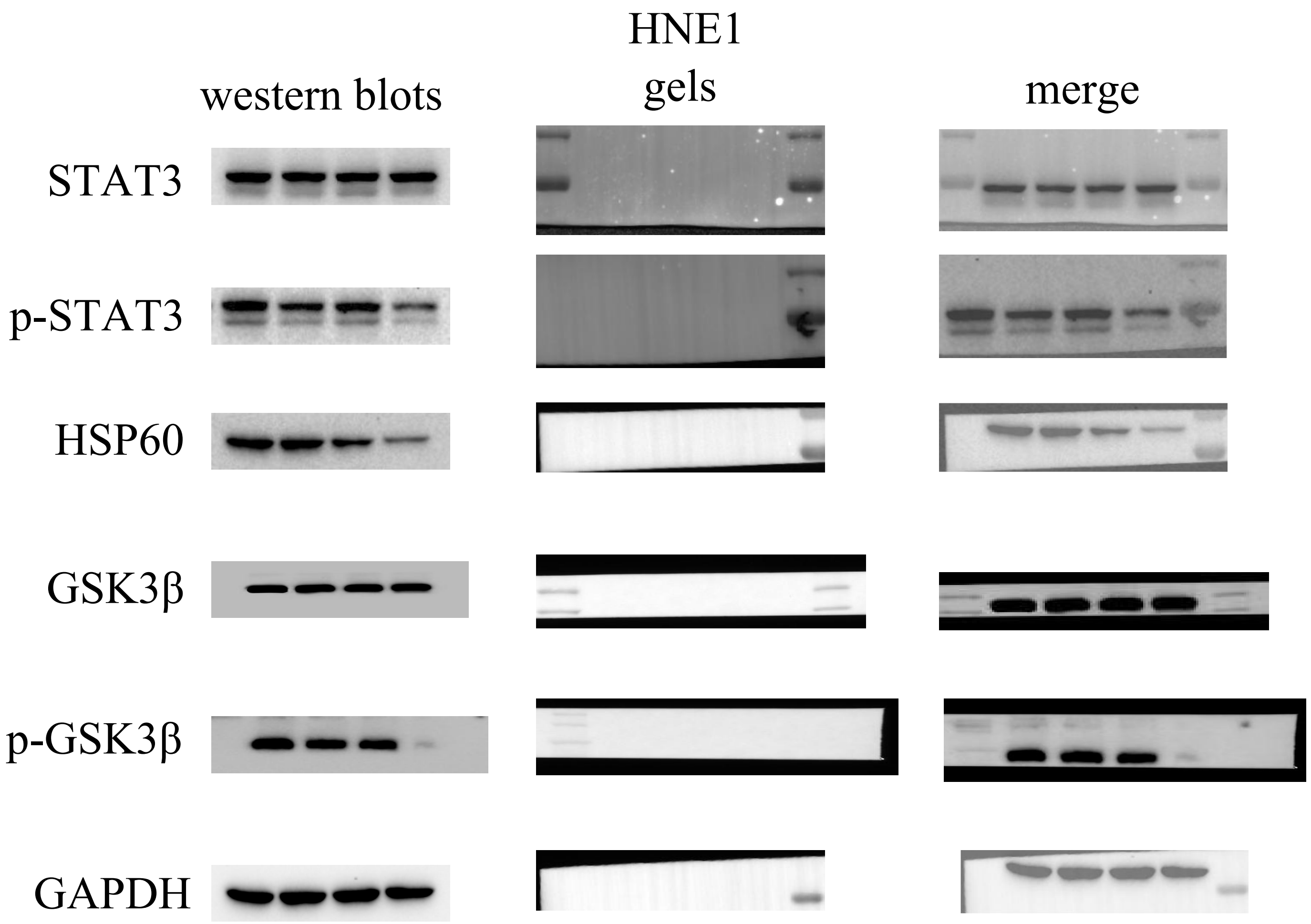
HNE1
western blots
gels
merge
STAT3
p-STAT3
HSP60
GSK3β
p-GSK3β
GAPDH

Figure 6C

Original images

CNE1

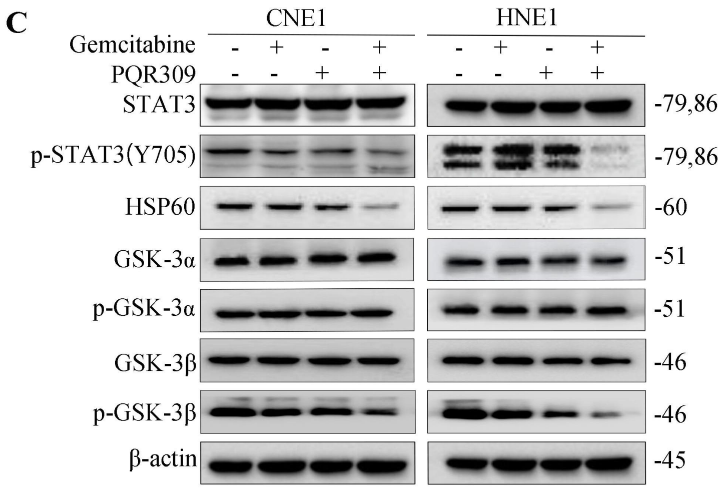

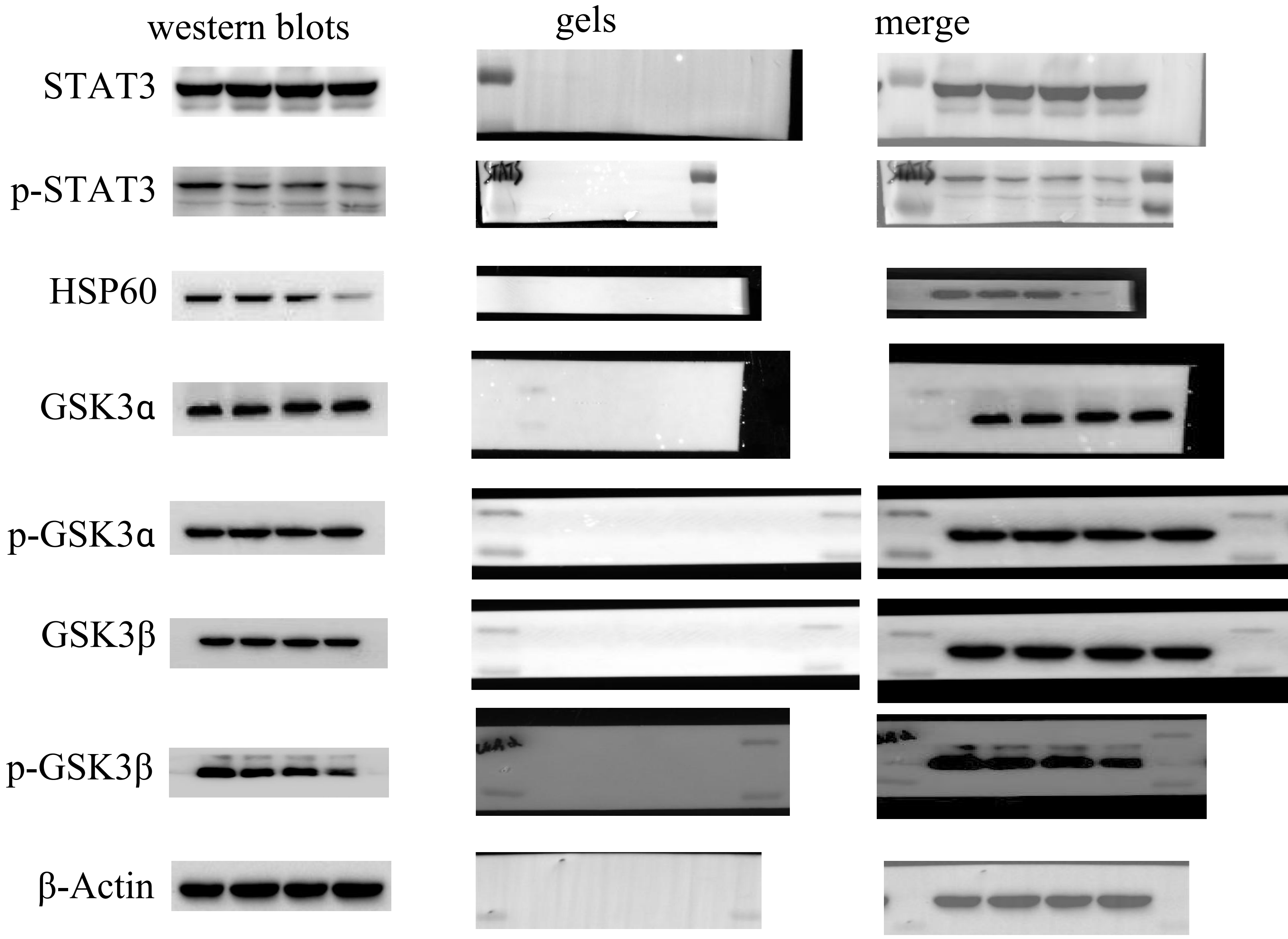

Figure 6C

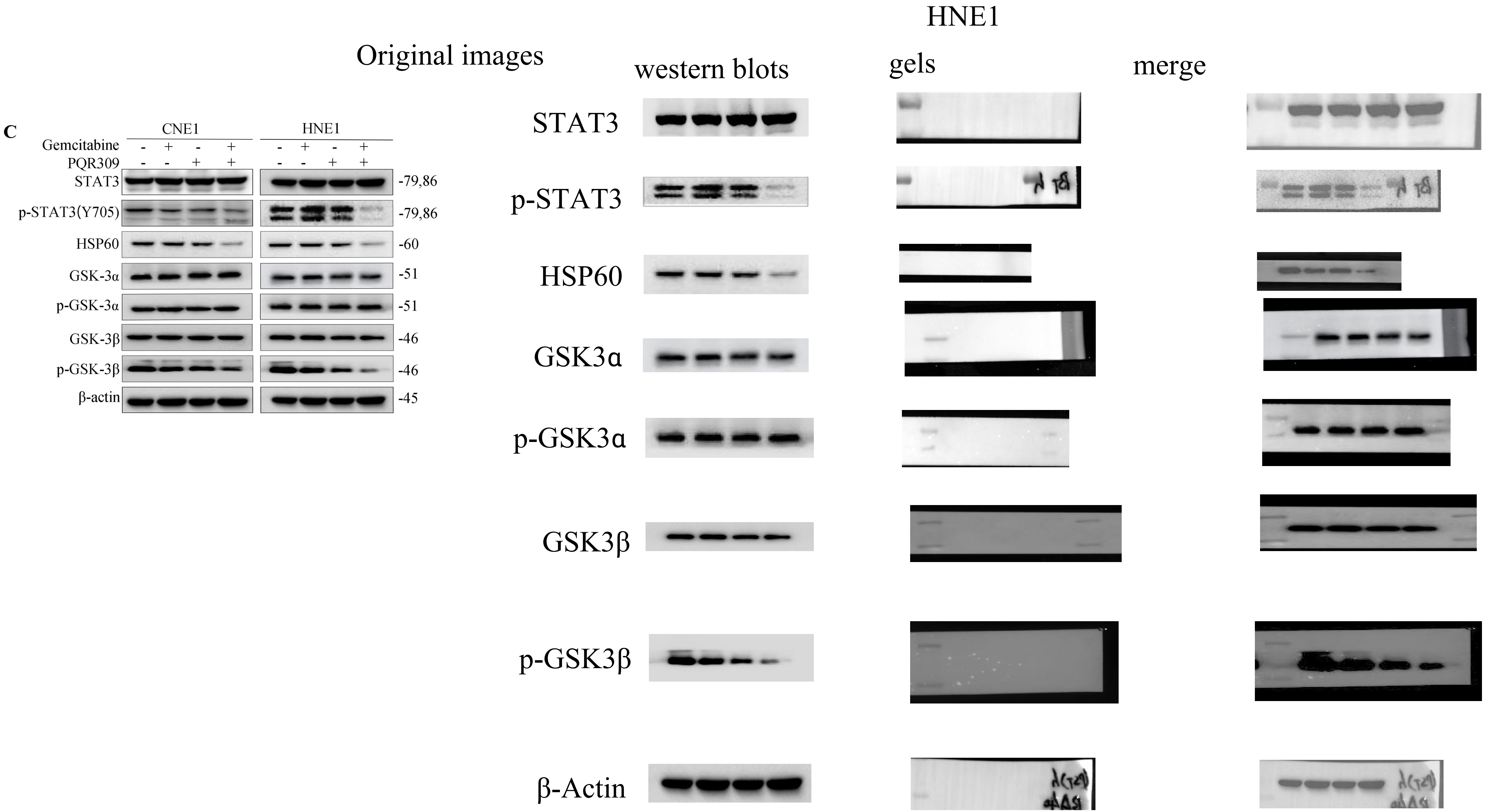
HNE1
Original images
western blots
gels
merge
C
CNE1
HNE1
Gemcitabine - + - + - + - +
PQR309 - - + + - - + +
STAT3 -79,86
p-STAT3(Y705) -79,86
HSP60 -60
GSK-3α -51
p-GSK-3α -51
GSK-3β -46
p-GSK-3β -46
β-actin -45
STAT3
p-STAT3
HSP60
GSK3α
p-GSK3α
GSK3β
p-GSK3β
β-Actin

Supplementary Figure 2O

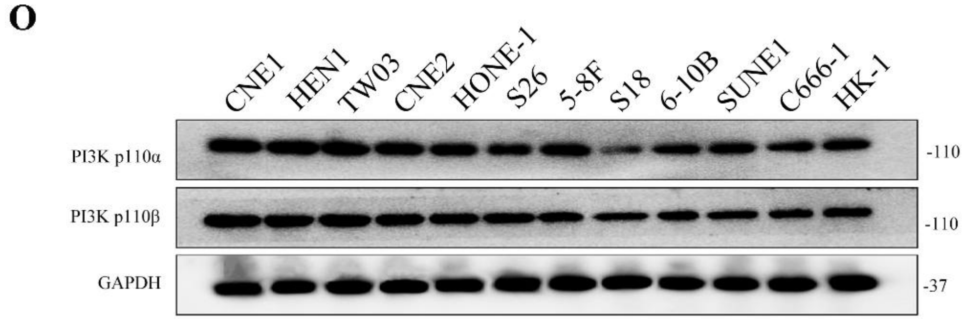

Original images

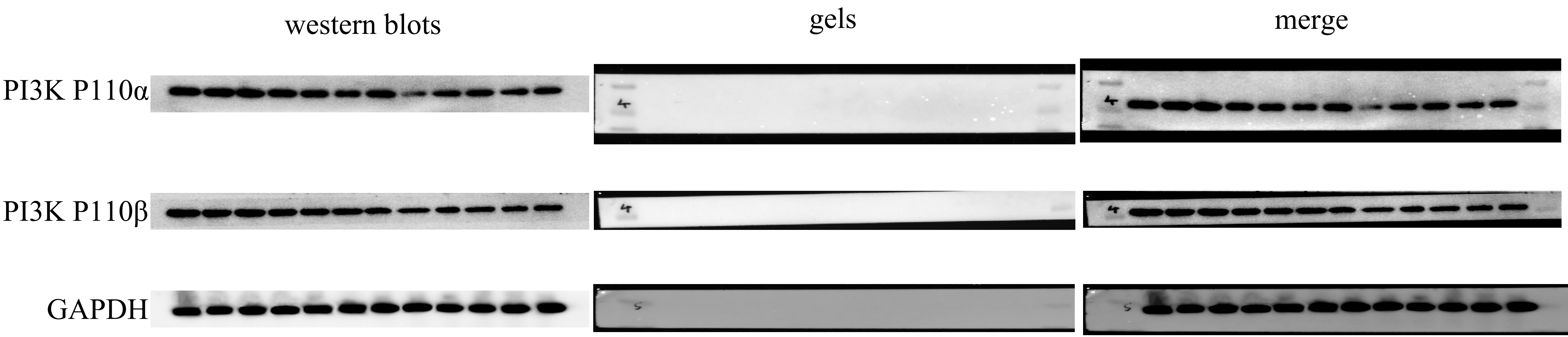

Supplementary Figure 2Q and 2R

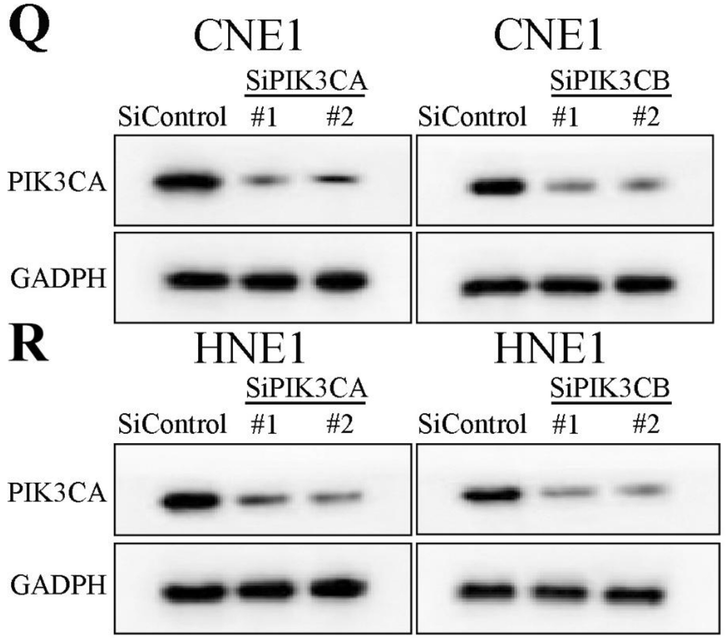

Original images

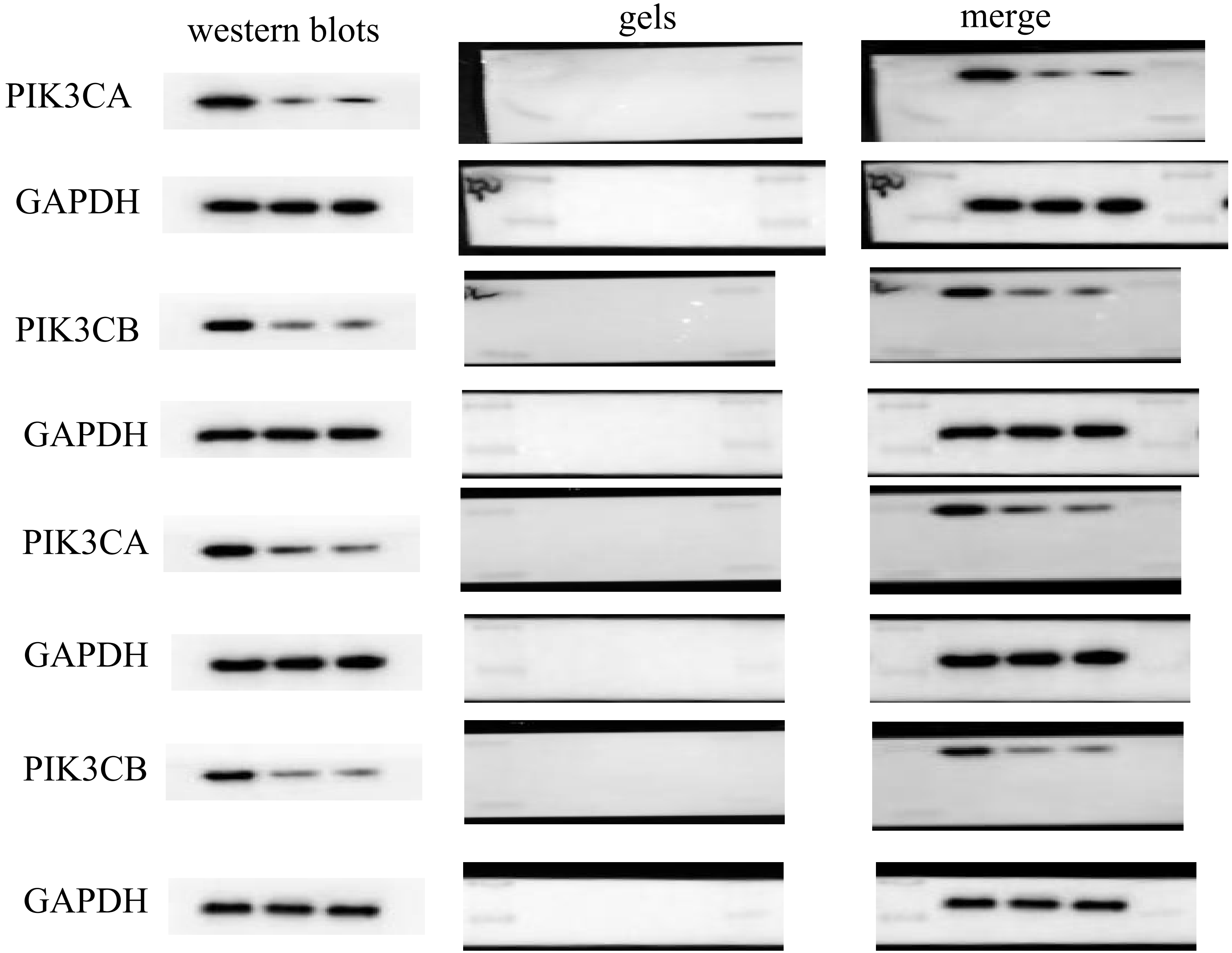

Supplementary Figure 6A

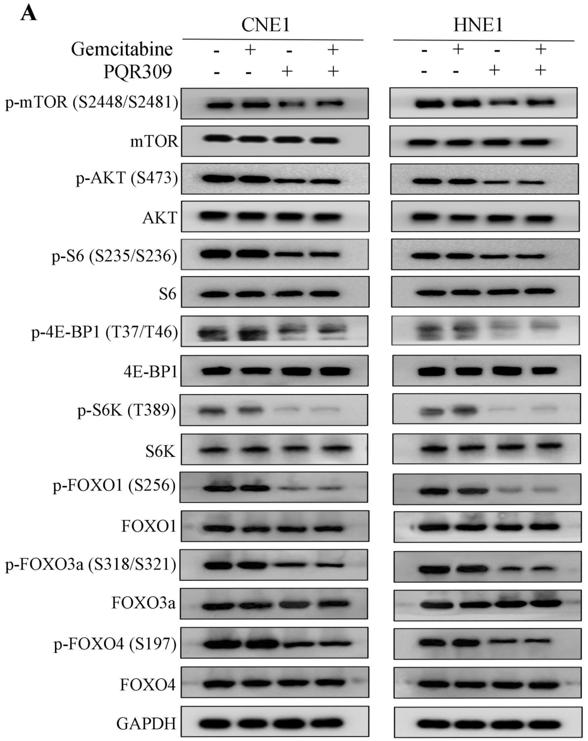

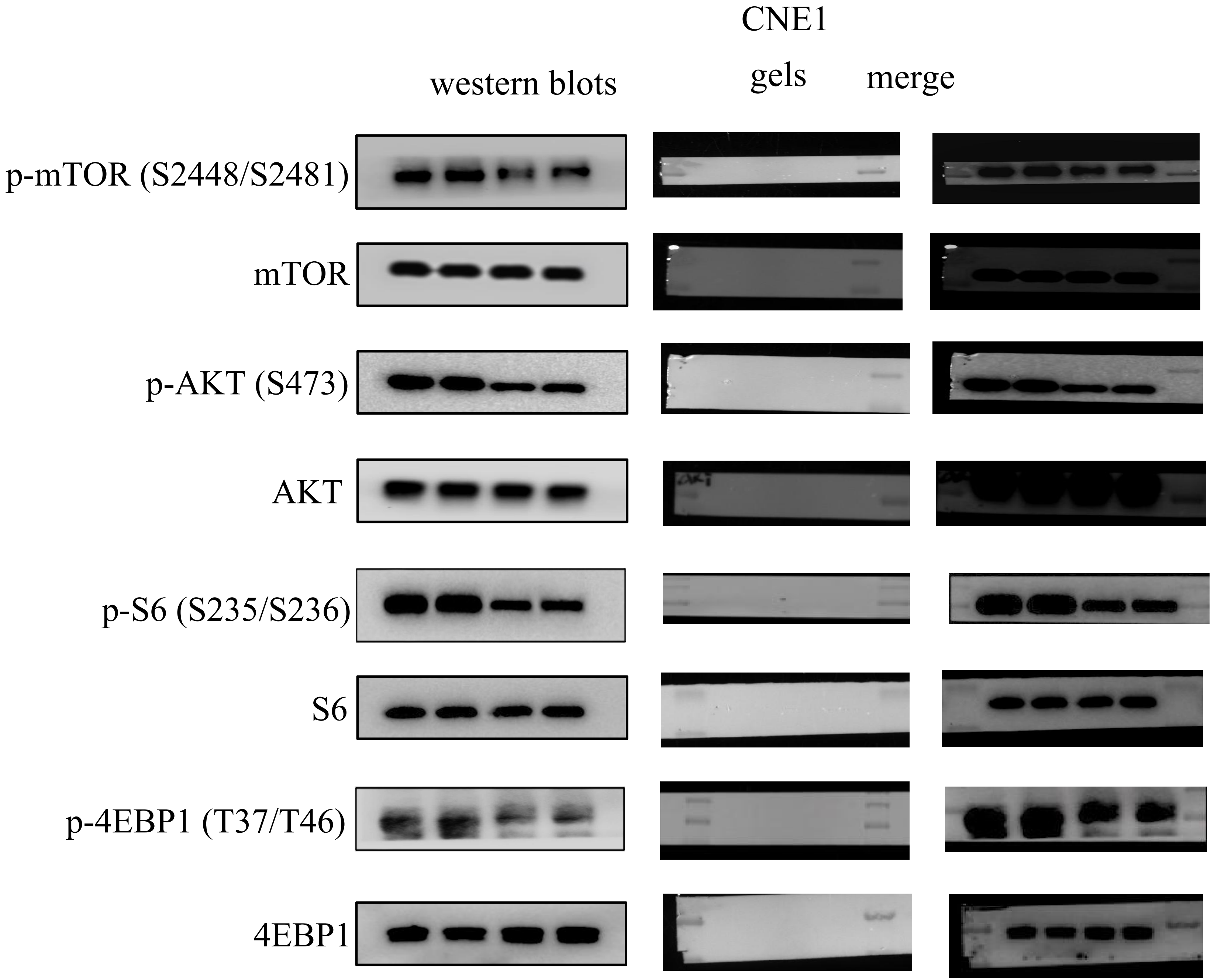

Supplementary Figure 6A

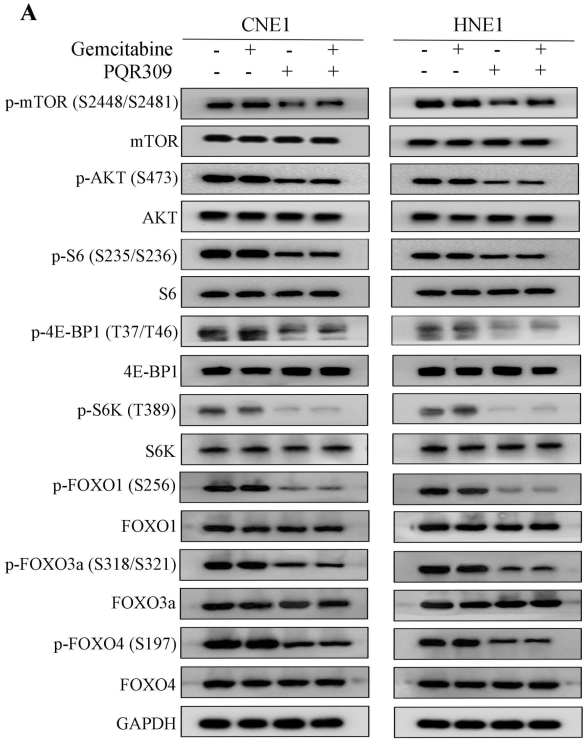

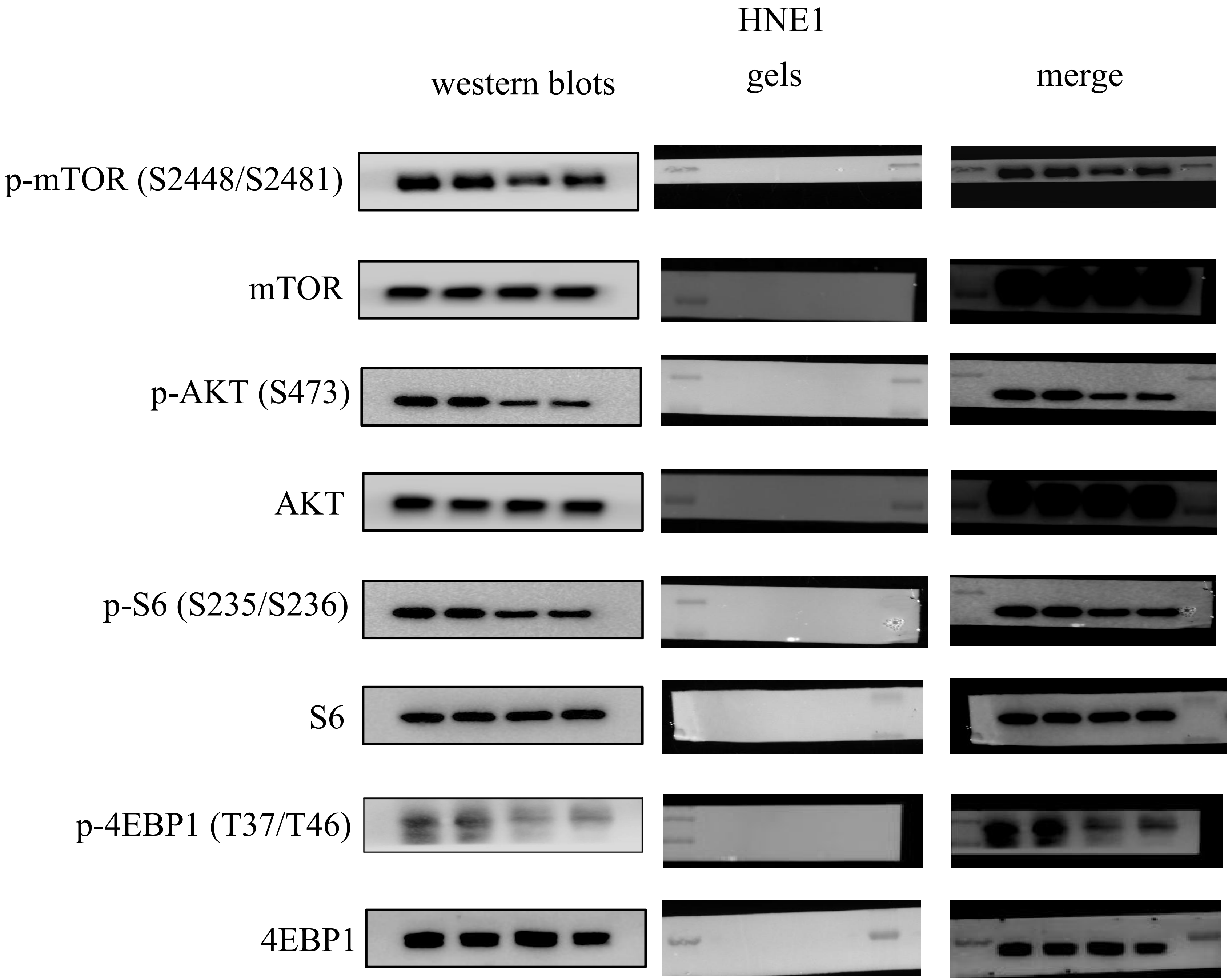

Supplementary Figure 6A

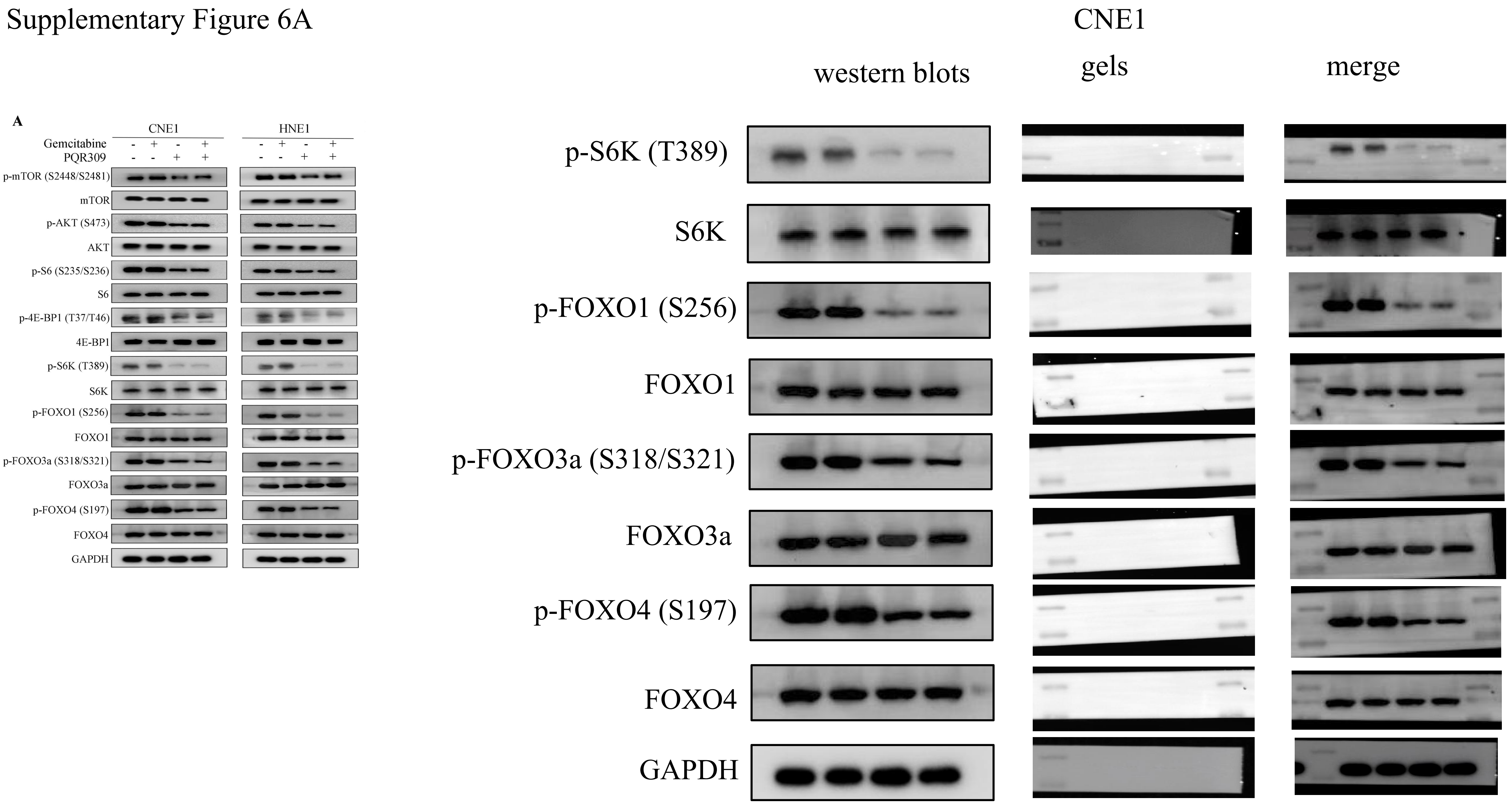

Supplementary Figure 6A

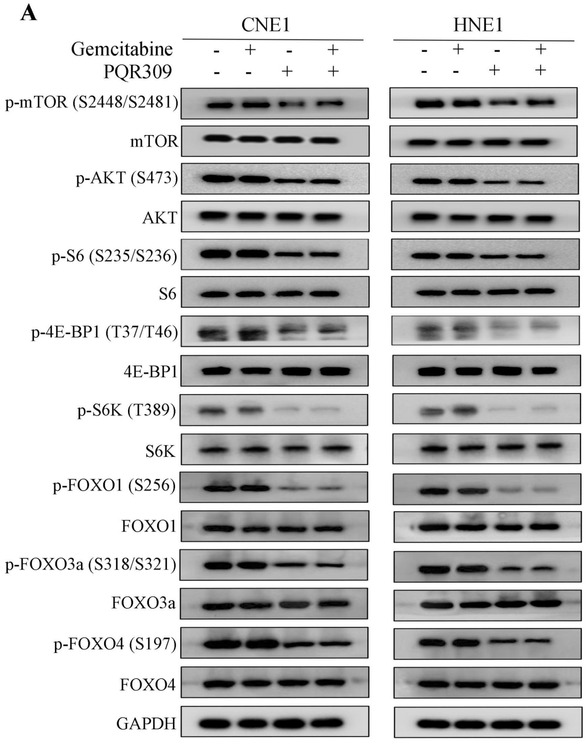

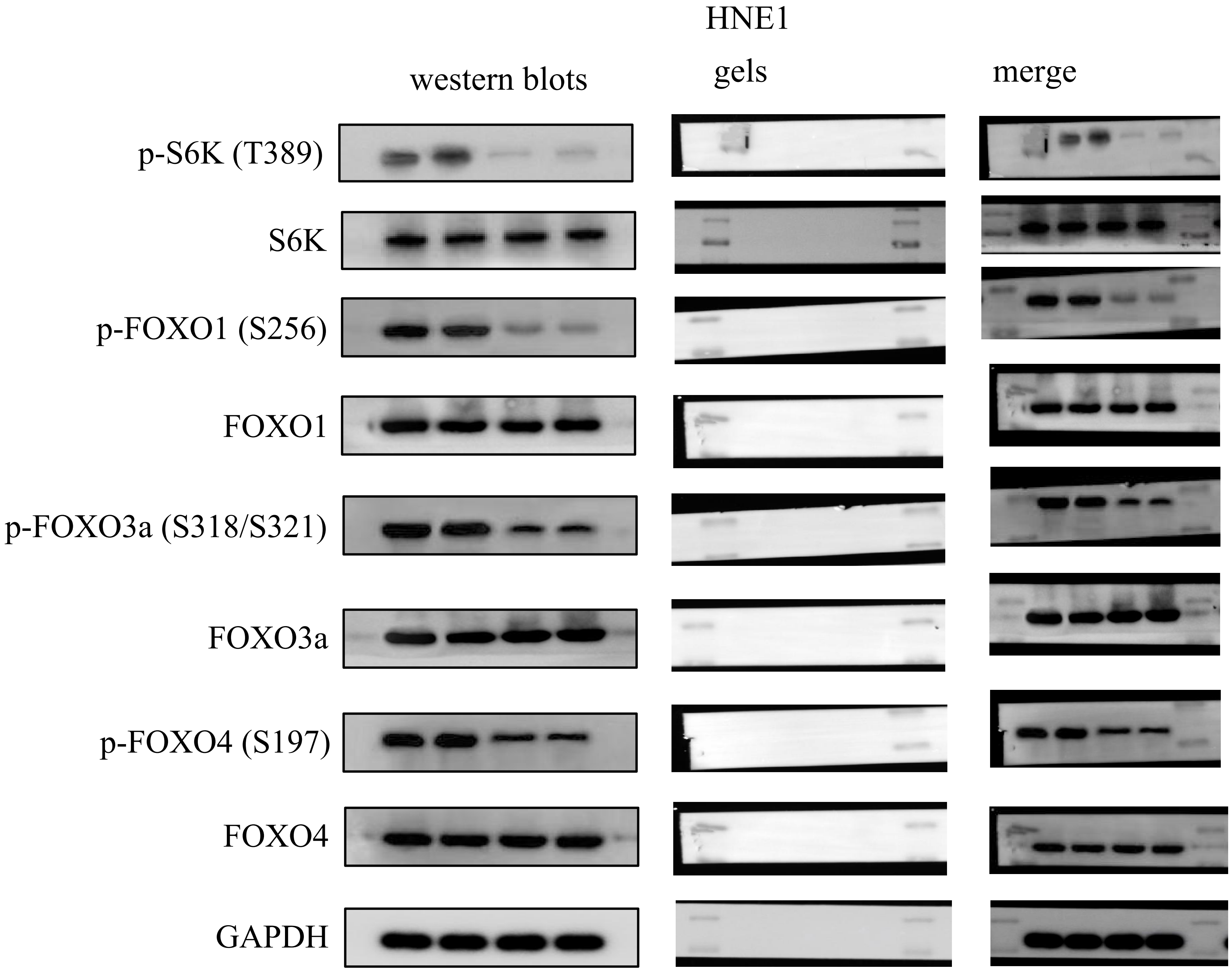

Supplementary Figure 7A

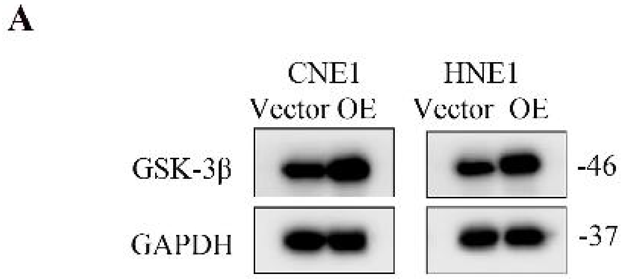

Original images

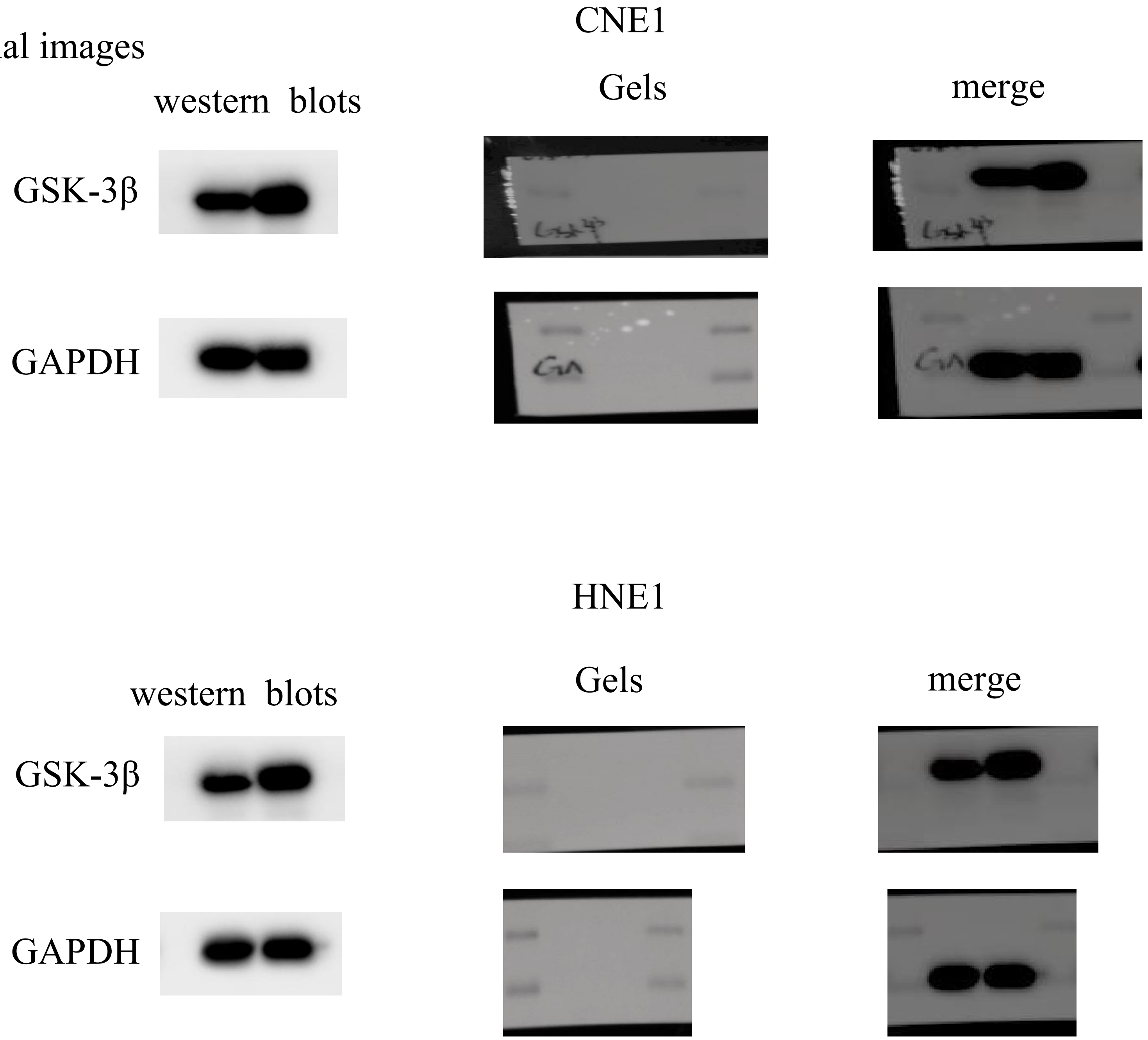

Supplementary Figure 7E

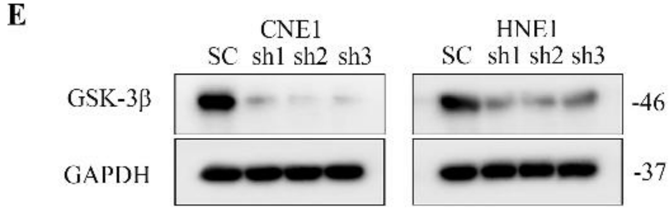

Original images

CNE1

western blots

GSK-3β

GAPDH

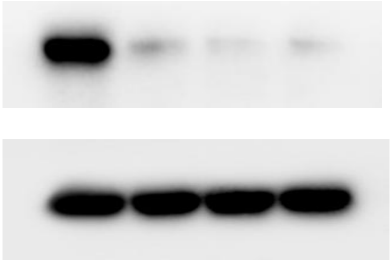

gels

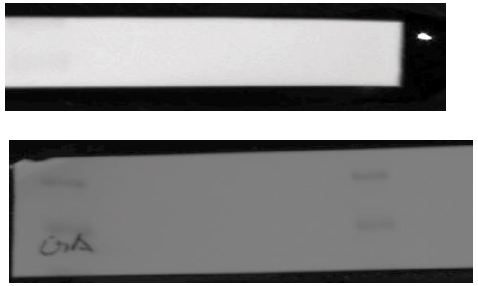

merge

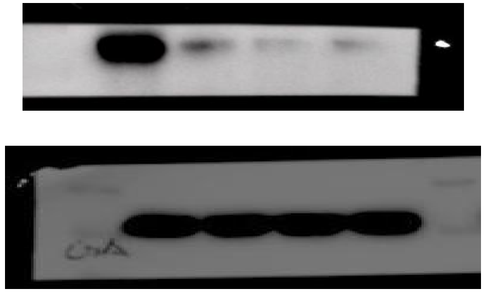

HNE1

western blots

GSK-3β

GAPDH

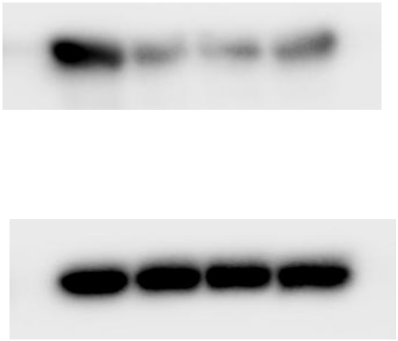

gels

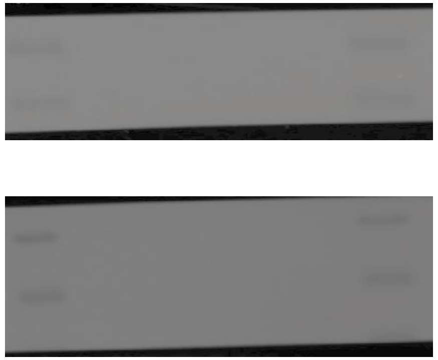

merge

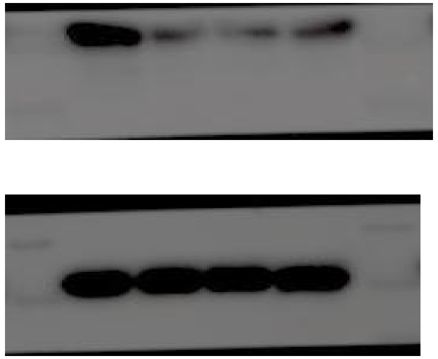

Supplementary Figure 8A

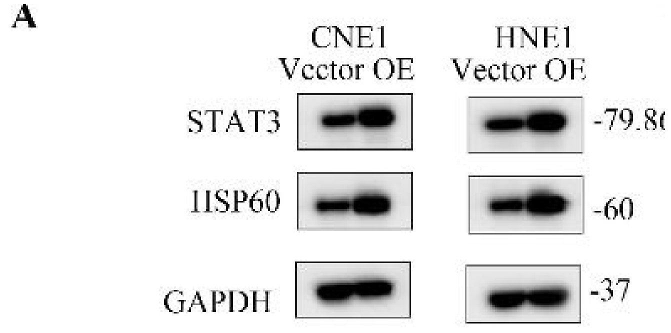

Original images

CNE1

western blots | gels | merge

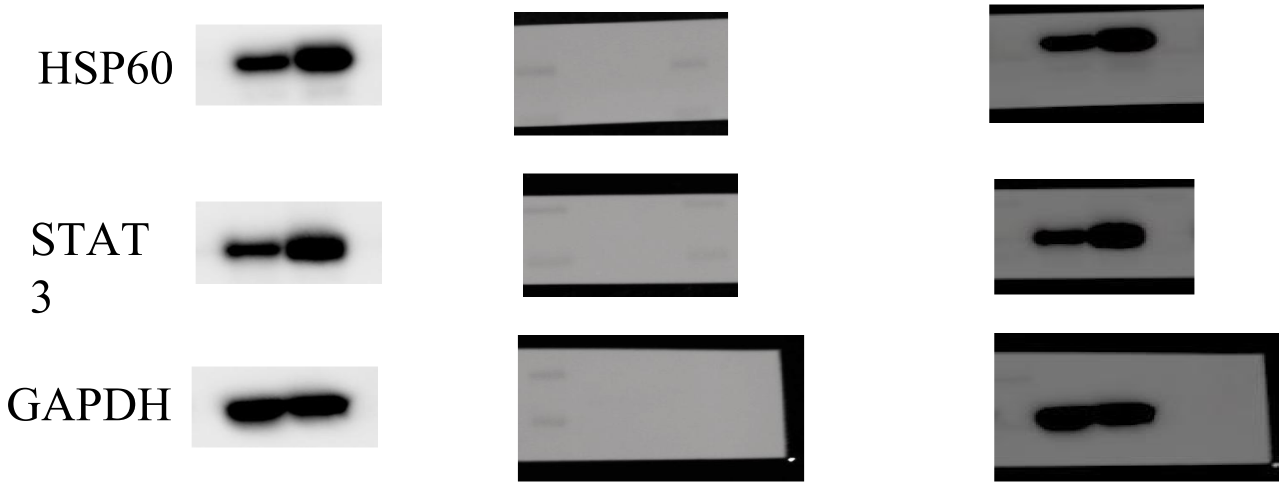

HNE1

western blots | gels | merge

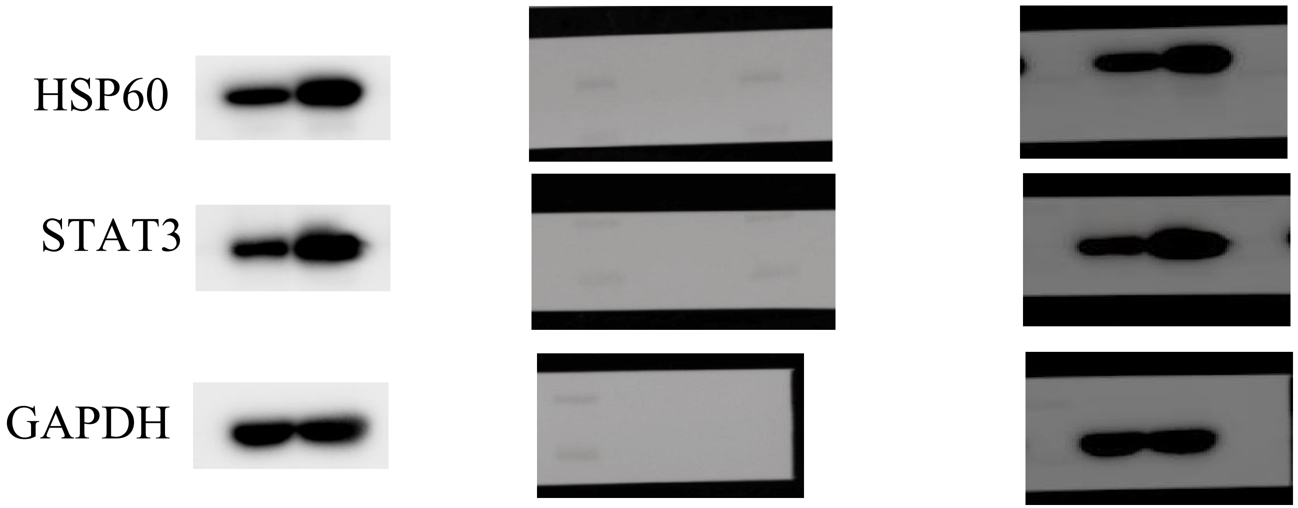

Supplementary Figure 8E

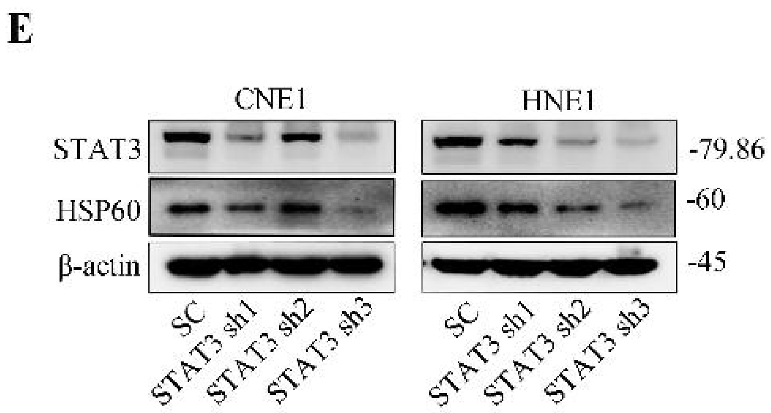

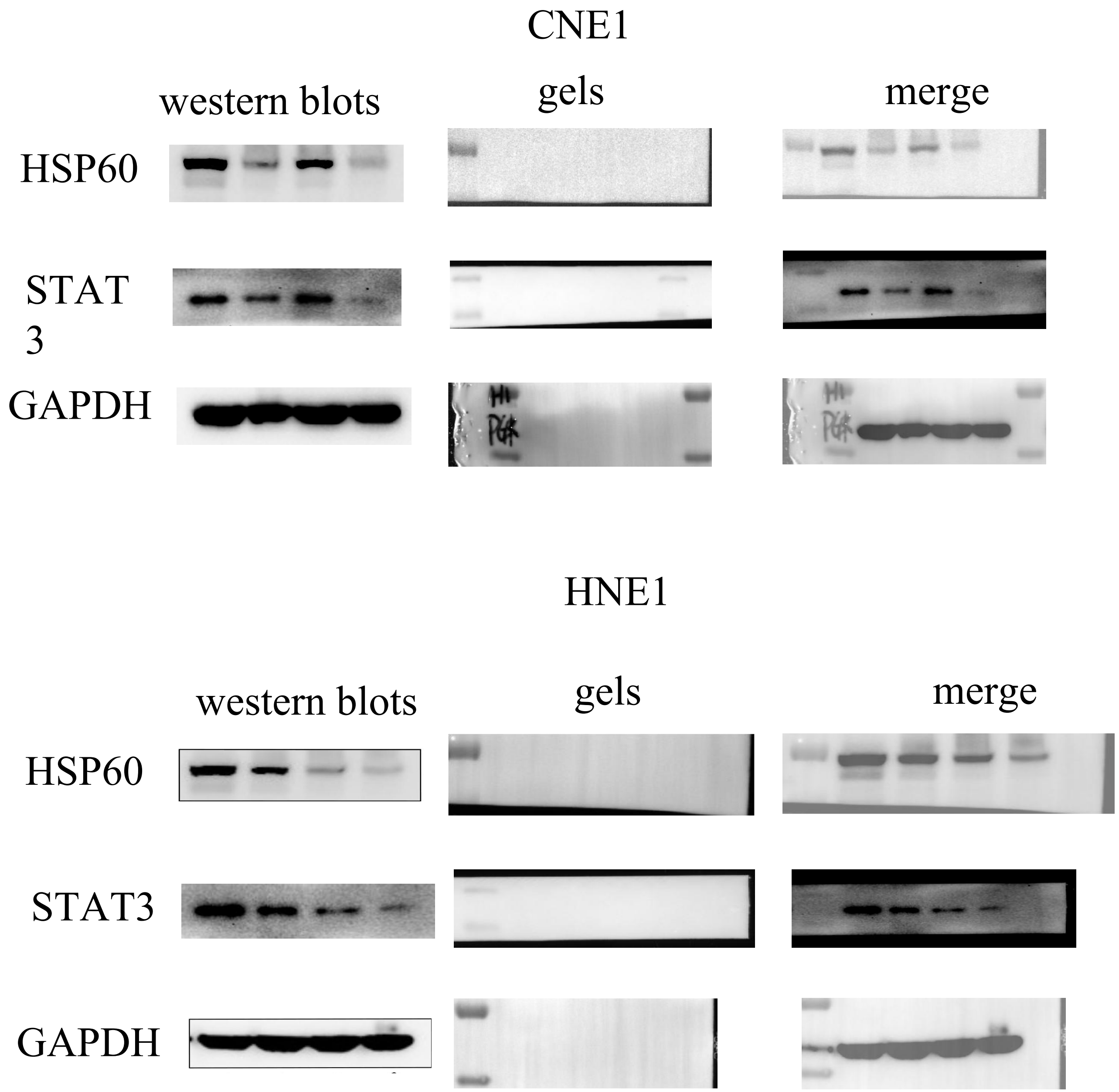

Supplementary Figure 9A

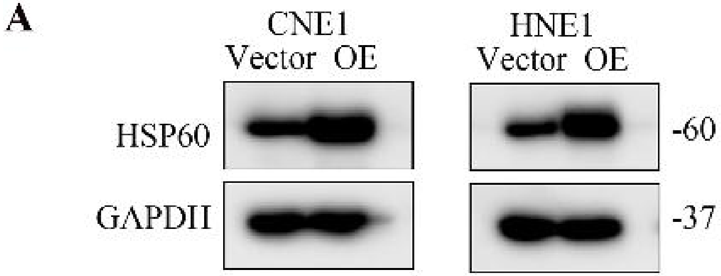

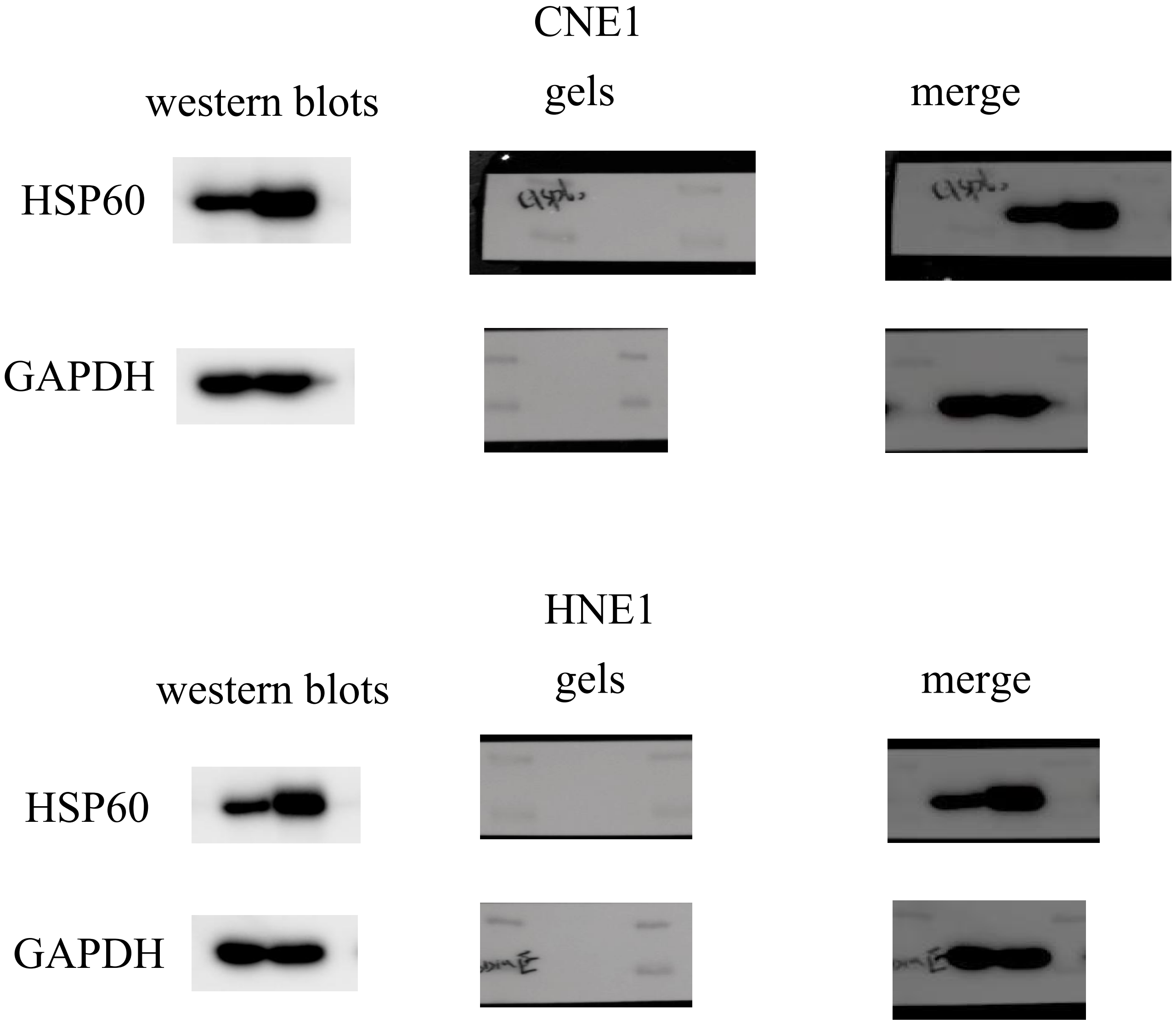

Supplementary Figure 9E

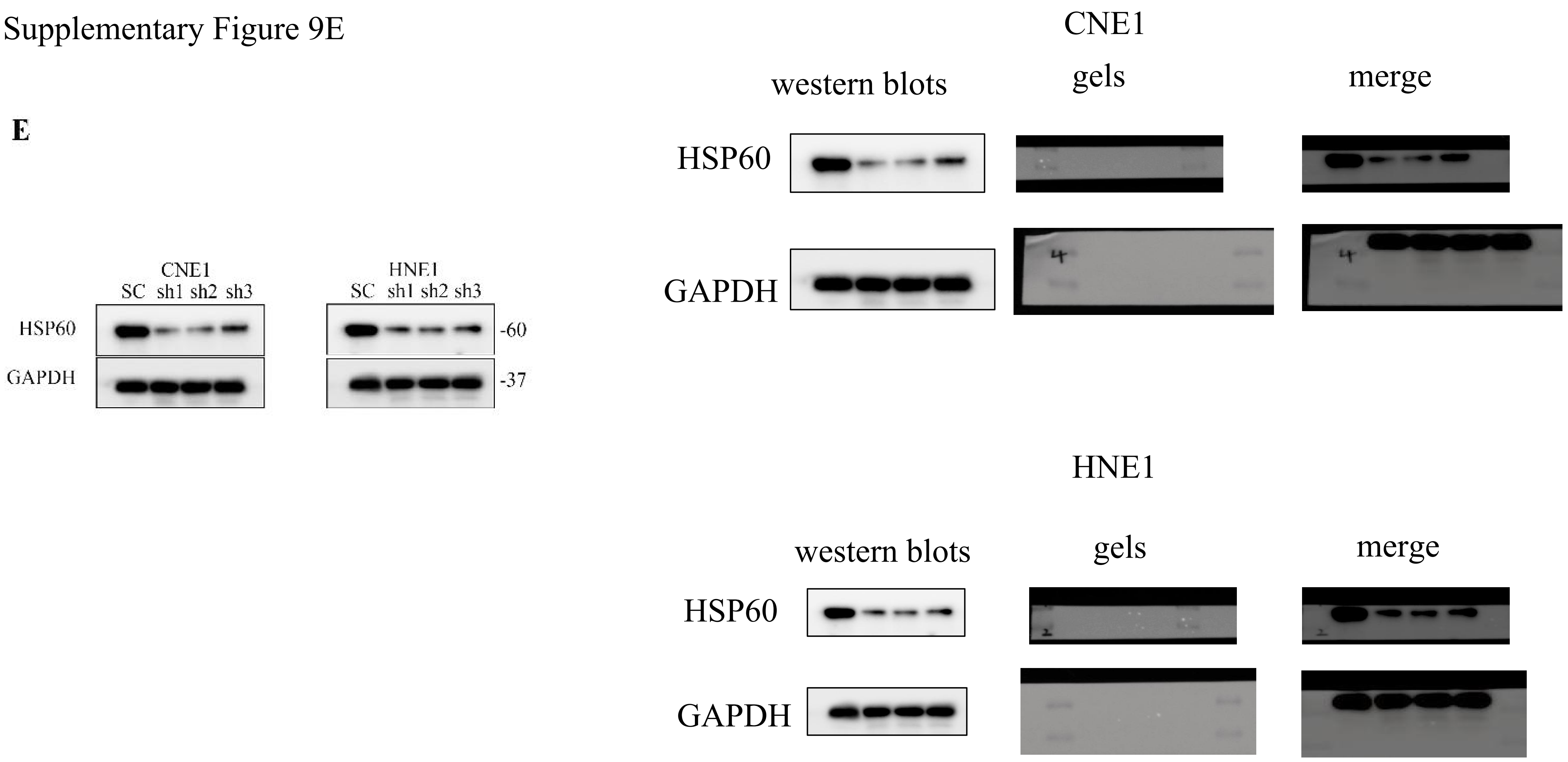

Supplement: Supplementary file 3 — Original Data File-western blots [file 41419_2024_6615_MOESM3_ESM.pdf]
